# Trends and projections of dermatitis burden (1990–2040): a 2021 global burden of disease analysis

**DOI:** 10.3389/fmed.2026.1696683

**Published:** 2026-01-28

**Authors:** Jing Fang, Guo Chen, Mengyu Wu, Yi Shi, Chen Yuan, Wenyuan Liu

**Affiliations:** 1 Department of Dermatology, Changxing County Traditional Chinese Medicine Hospital, Huzhou, China; 2 Department of Pediatrics, The First Affiliated Hospital of Huzhou University, Huzhou, China

**Keywords:** DALYs, dermatitis, epidemiology, Global Burden of Disease 2021, incidence

## Abstract

**Background:**

Dermatitis is a major global public health challenge. This study evaluates its burden from 1990 to 2021 and projects future burden over the next 20 years.

**Methods:**

Data from the Global Burden of Disease Study 2021 (GBD 2021) included prevalence, incidence, disability-adjusted life years (DALYs), and age-standardized rates (ASRs), stratified by year, region, age, and Sociodemographic Index (SDI). Analyses used estimated annual percentage change (EAPC), inequality indices, decomposition, and Bayesian age-period-cohort (BAPC) modeling for forecasting.

**Results:**

In 2021, global dermatitis cases reached 241 million (95% UI: 223.3–262.5 million), a 38.8% increase since 1990, but the age-standardized prevalence rate (ASPR) declined to 3,075.1 per 100,000 (EAPC = −0.14). Incident cases were 405 million (95% UI: 356–467 million), with the age-standardized incidence rates (ASIR) slightly increased to 4,945 per 100,000 (EAPC = 0.04). DALYs totaled 8.18 million (95% UI: 4.85–13.0 million), with the age-standardized DALY rate (ASDR) declining to 106.2 per 100,000 (EAPC = −0.18). ASPR and ASDR were highest in high-SDI regions and lowest in low-SDI regions; ASIR negatively correlated with SDI. Incidence peaked at ages 5–9 years. The burden was higher in males. A sharp DALYs rise occurred in those ≥65 years, especially in low/low-middle SDI regions. Projections indicate ASIR will slightly increase to 4,966 per 100,000 by 2040, while ASDR declines to 103 per 100,000.

**Conclusion:**

Despite rising absolute cases globally (driven by population growth), declining ASRs suggest diagnostic/therapeutic improvements. Marked disparities persist across SDI levels, age groups, and sexes. Strengthened prevention and resource allocation in low- and middle-income countries are needed to reduce health disparities.

## Introduction

Dermatitis—including atopic dermatitis (AD), contact dermatitis (CD), and seborrheic dermatitis (SD)—represents one of the most burdensome categories among skin diseases globally (1). According to the latest Global Burden of Disease (GBD) analysis, AD is the leading contributor to the global burden of skin conditions, with a global prevalence of approximately 2.6%, affecting an estimated 204 million people. In 2021, the number of AD cases reached 129 million, marking a 20% increase from 1990 (1). Other types of dermatitis are also highly prevalent: in 2021, there were approximately 253 million new cases of CD, the most common form of dermatitis, and 135.7 million new cases of SD (2, 3). A recent meta-analysis reported a global prevalence of SD at around 4.38% (4).

Although dermatitis is rarely fatal, its chronic symptoms—such as itching, pain, and visible lesions—can substantially impair quality of life and result in hundreds of thousands of disability-adjusted life years (DALYs) annually (5). The disease burden is particularly pronounced among children and working-age adults: early-onset AD disrupts sleep and development, while occupational CD leads to absenteeism and significant economic losses (6–8). Environmental changes (e.g., increasing pollution, climate variability) and lifestyle transitions (e.g., urbanization, altered microbial exposure) are considered key drivers of the recent rise in dermatitis incidence in both high- and low-income countries (9–13).

Given the considerable public health impact of dermatitis and its evolving epidemiological patterns, a nuanced understanding of its global distribution and underlying drivers is essential for informing prevention strategies, optimizing disease management, and mitigating its socioeconomic burden. Although several recent GBD 2021–based studies have examined the global burden and trends of specific dermatitis subtypes—such as atopic dermatitis, seborrheic dermatitis, or broader allergic skin diseases—most existing analyses remain subtype-focused. Consequently, a systematic evaluation of dermatitis as an aggregated cause within the GBD hierarchy, together with its inequality patterns and structural drivers of change, remains limited. Leveraging data from the Global Burden of Disease 2021 study, which provides a standardized and comparable framework for quantifying disease burden across countries and over time, this study aims to (1) characterize long-term trends in the global, regional, and national burden of dermatitis from 1990 to 2021, (2) assess socioeconomic inequalities associated with dermatitis burden, (3) decompose changes in burden into contributions from population growth, population aging, and epidemiological change, and (4) project future burden through 2040.

## Methods

### Case definition and data extraction

Dermatitis burden data were obtained from the Global Burden of Disease (GBD) 2021 study using the Global Health Data Exchange (GHDx) query tool. In this study, we adopted the standardized GBD cause hierarchy to ensure full transparency and reproducibility. Specifically, all analyses were based on the Level 3 cause “Dermatitis” (Cause ID = 654), which belongs to the parent category Skin and subcutaneous diseases (Cause ID = 653). No customized aggregation, reclassification, or modification of GBD causes was performed. According to the GBD hierarchy, Level 3 Dermatitis comprises the following Level 4 sub-causes: Atopic dermatitis (Cause ID = 977). Contact dermatitis (Cause ID = 978). Seborrhoeic dermatitis (Cause ID = 979). These three Level 4 conditions represent the clinically recognized subtypes of dermatitis. However, GBD uses Level 3 causes as the primary analytic unit in global and regional comparative assessments because they provide more stable estimates and facilitate cross-country comparability. Therefore, all epidemiological indicators in this study—including incidence, prevalence and disability-adjusted life years (DALYs)—refer exclusively to Level 3 Dermatitis (Cause ID 654).

Data were extracted for all countries and territories from 1990 to 2021 using the standard GBD definitions, modeling approaches, and uncertainty intervals (UIs). GBD categorizes the world into seven super-regions, which are further subdivided into 21 GBD regions (14). In addition, the Sociodemographic Index (SDI) is used as a composite indicator to assess a country or regions socioeconomic development level (10). Based on SDI scores, countries and regions are classified into five groups: high SDI, high-middle SDI, middle SDI, low-middle SDI, and low SDI, facilitating comparisons of health disparities across development levels.

### Data processing and burden estimation

The burden of dermatitis was quantified using prevalence, incidence, and DALYs. Data were extracted for 20 age groups (in 5-year intervals), age-standardized rates (ASRs), all-age data, and corresponding 95% uncertainty intervals (UIs). Assuming a linear relationship between the natural logarithm of ASR and calendar year, we used the estimated annual percentage change (EAPC)—a widely applied method—to quantify temporal trends in ASRs over the study period.

### Health inequality analysis

Health inequality refers to measurable differences in health outcomes between population subgroups defined by socioeconomic, geographic, or demographic characteristics (15, 16). We employed the slope index of inequality (SII) and the concentration index (CIX) to assess absolute and relative inequalities, respectively, in dermatitis-related prevalence, incidence and DALYs (17). SII was calculated using weighted regression to quantify differences across the cumulative SDI distribution, while CIX was derived from the area under the Lorenz concentration curve based on SDI rankings (18).

### Decomposition analysis

Decomposition analysis is a valuable statistical method in epidemiology and public health that helps identify and quantify the contributions of various drivers to changes in disease burden over time (19). To identify key factors influencing DALY changes from 1990 to 2021, we applied decomposition analysis to disentangle the effects of population growth, population aging, and epidemiological changes.

### Correlation and projection analysis

We employed the Bayesian age-period-cohort (BAPC) model to predict the future burden of dermatitis up to 2050. The BAPC model demonstrates significant advantages in handling the complex, high-dimensional, and sparse data commonly encountered in large-scale epidemiological studies such as the Global Burden of Disease Study 2021 (GBD 2021). Built upon the traditional generalized linear model framework within a Bayesian statistical context, the model dynamically integrates age, period, and cohort effects. These effects are assumed to evolve continuously over time and are smoothed using a second-order random walk, thereby yielding more accurate posterior probability predictions. The Integrated Nested Laplace Approximation (INLA) framework was used in conjunction with the BAPC model to approximate marginal posterior distributions, effectively circumventing the mixing and convergence issues associated with traditional Bayesian methods that rely on Markov chain Monte Carlo (MCMC) sampling techniques. The model’s flexibility and robustness in processing time-series data make it particularly suitable for long-term disease burden prediction. Therefore, we adopted the BAPC model implemented within the INLA framework due to its computational efficiency and lower error rates compared to conventional approaches. This method enables comprehensive consideration of the intricate interactions among age, period, and cohort effects, thereby facilitating nuanced predictions of future disease burden (17).

All data processing and visualization were conducted using the World Health Organization (WHO) Health Equity Assessment Toolkit and R statistical software (version 4.3.3).

## Results

**Figure 1:**
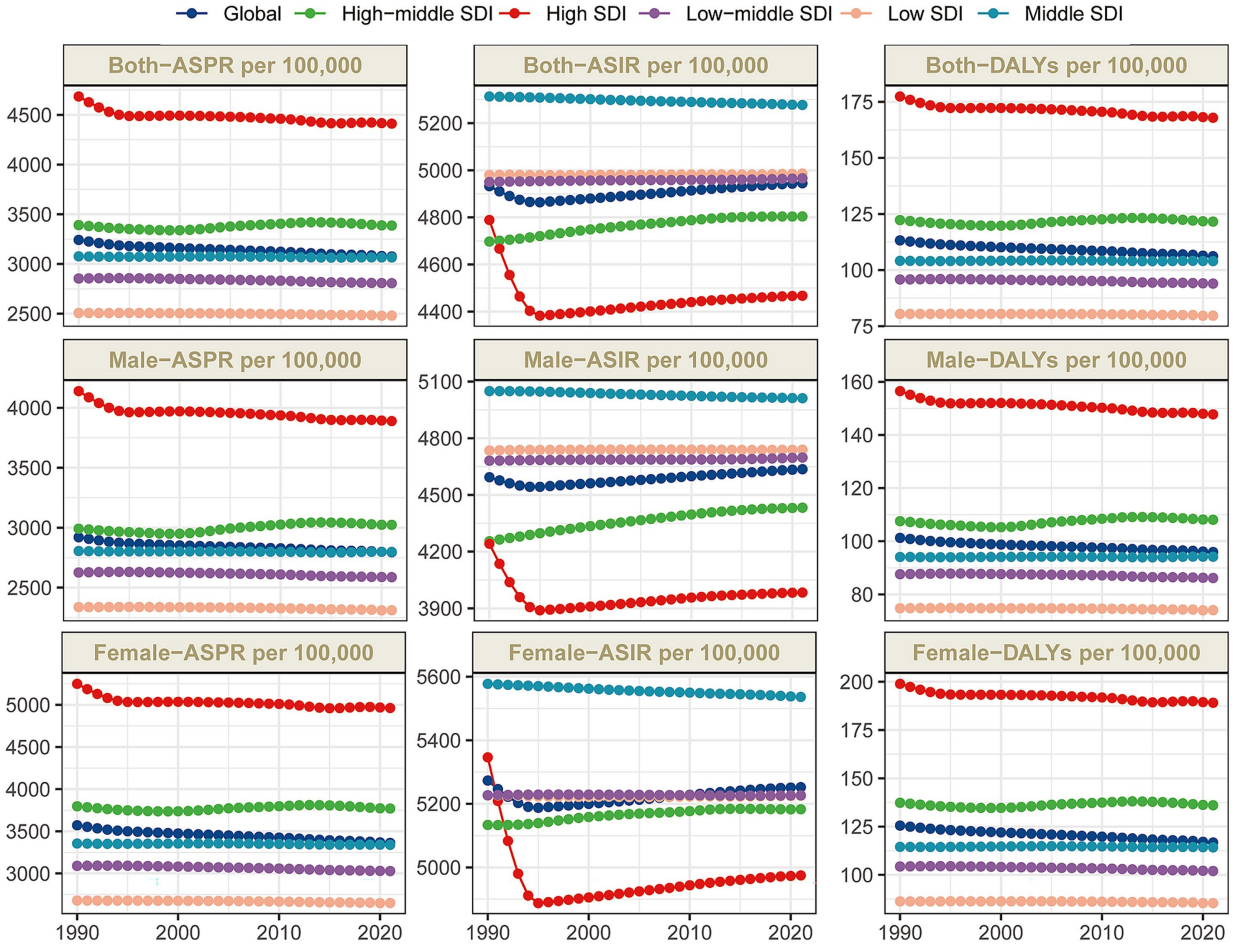
Trends in dermatitis prevalence, incidence, and disability-adjusted life-years from 1990 to 2021.

The global burden of dermatitis remained substantial in 2021. The estimated number of prevalent cases of dermatitis reached 240,887,382.8 (95% uncertainty interval [UI]: 223,263,286.3–262,498,101.3) in 2021, representing a significant increase compared with 173,562,301.5 cases in 1990 (95% UI: 162,554,326.2–187,284,319.6). Despite the increase in absolute case numbers, the age-standardized prevalence rate (ASPR) declined from 3,242.3 per 100,000 population in 1990 (95% UI: 3,028.3–3,505.5) to 3,075.1 per 100,000 in 2021 (95% UI: 2,863.3–3,340.7). Over the study period, the estimated annual percentage change (EAPC) in ASPR was −0.14339 (95% confidence interval [CI]: −0.15274 to −0.13404), indicating a slow but consistent downward trend in prevalence (Table 1; Figure 1). Regarding incidence, the number of new cases of dermatitis globally was estimated at 405,021,857.7 (95% UI: 355,572,501.6–467,256,268.8) in 2021, up markedly from 248,056,086.1 cases in 1990 (95% UI: 218,593,920.0–284,715,695.8). Over the same period, the age-standardized incidence rate (ASIR) increased slightly from 4,932.4 per 100,000 population in 1990 (95% UI: 4,334.7–5,691.6) to 4,945.0 per 100,000 in 2021 (95% UI: 4,354.9–5,690.3). The EAPC for ASIR was 0.04407 (95% CI: 0.03169 to 0.05645), indicating a mild upward trend in global dermatitis incidence (Table 1; Figure 1). As for disability-adjusted life years (DALYs), the total number attributable to dermatitis in 2021 was 8,177,101.6 (95% UI: 4,847,630.1–12,991,190.8). The age-standardized DALY rate (ASDR) decreased from 113.3 per 100,000 population in 1990 (95% UI: 66.3–181.3) to 106.2 per 100,000 in 2021 (95% UI: 62.5–169.5). The corresponding EAPC was −0.18385 (95% CI: −0.19122 to −0.17649), reflecting a sustained downward trend in DALY burden due to dermatitis (Table 1; Supplementary Table S1).

**Table 1:** Global and regional trends in dermatitis burden: prevalence (1990–2021).

Location	Number 1990	ASR 1990	Number 2021	ASR 2021	EAPC_95%CI
Prevalence					
Andean Latin America	1215459.2 (1133978.5–1314699.4)	3181.9 (2945.8–3481.9)	2077847.2 (1916236.6–2280665.6)	3177.2 (2941.6–3477.7)	0.00032 (−0.00539 to 0.00603)
Australasia	565935.5 (540190.3–596190.9)	3058.8 (2914.5–3223.2)	812503.9 (775807.5–853531.3)	3061.4 (2914.2–3228.8)	0.00681 (−0.00275 to 0.01638)
Caribbean	1147289.2 (1070210.9–1241030.3)	3240.5 (3014.5–3513.3)	1511669.8 (1402746.9–1650582.4)	3238.6 (3012.7–3512.2)	−0.00246 (−0.00266 to −0.00226)
Central Asia	4402039.9 (4161257.3–4658872.9)	5768.1 (5451.7–6140.9)	5491025.5 (5193320.6–5849017.9)	5750.7 (5434.4–6120.4)	−0.00941 (−0.01018 to −0.00864)
Central Europe	3875067.1 (3526389.1–4263388.4)	3191.5 (2945.9–3494.7)	3512344.6 (3145349.2–3974456.2)	3206.8 (2960.8–3513.1)	0.01336 (0.01024 to 0.01648)
Central Latin America	5287777.1 (4943815.1–5680725.4)	3158.7 (2929–3429.7)	7806700.8 (7206407.5–8531787.4)	3143.9 (2912.6–3412.4)	−0.01818 (−0.02262 to −0.01374)
Central Sub-Saharan Africa	1219239.3 (1134728.3–1315748.3)	2229.4 (2049.4–2454.4)	3039785.1 (2825993.3–3287924.8)	2228.9 (2048.4–2453.1)	−0.00215 (−0.00312 to −0.00118)
East Asia	34249122.2 (31003252.7–38,083,719)	2,905 (2637.1–3215.3)	44063170.5 (39232257.2–50084648.9)	2893.6 (2634.7–3200.1)	0.00204 (−0.00827 to 0.01236)
Eastern Europe	8826664.3 (8323504.8–9441629.6)	4155.8 (3944.9–4408.3)	7606803.5 (7143026.3–8208092.8)	4275.9 (4073.2–4,526)	0.1287 (0.10037 to 0.15704)
Eastern Sub-Saharan Africa	4288548.4 (4014990.4–4,599,976)	2,309 (2124.8–2539.6)	9650361.7 (9003468.7–10418921.8)	2323.9 (2143.8–2551.3)	0.02465 (0.02104 to 0.02825)
Global	173562301.5 (162554326.2–187284319.6)	3242.3 (3028.3–3505.5)	240887382.8 (223263286.3–262498101.3)	3075.1 (2863.3–3340.7)	−0.14339 (−0.15274 to −0.13404)
High SDI	38802700.9 (36684857.7–41163598.1)	4,685 (4451.5–4,951)	43620765.8 (41356836.1–46421891.8)	4411.7 (4200.5–4,653)	−0.12129 (−0.14741 to −0.09517)
High-income Asia Pacific	7801260.5 (7505236.1–8149594.7)	5144.4 (4942.8–5371.5)	7122780.3 (6872345.6–7430985.5)	5130.1 (4928.4–5,365)	−0.02261 (−0.04073 to −0.00447)
High-income North America	14939081.9 (13865869.4–16142492.9)	5365.6 (5005.8–5783.2)	17355573.7 (16370811.6–18607759.9)	4767.7 (4516.1–5069.4)	−0.17562 (−0.24725 to −0.10394)
High-middle SDI	35,212,778 (32912656.5–38048560.2)	3391.3 (3183.3–3,656)	41810463.6 (38228041.2–45,912,245)	3386.5 (3171.3–3665.8)	0.05827 (0.036 to 0.08055)
Low SDI	12765863.3 (11971475.1–13653764.8)	2,507 (2314.3–2740.8)	27709728.1 (25910054.4–29872927.6)	2480.4 (2288.6–2713.9)	−0.03574 (−0.03991 to −0.03157)
Low-middle SDI	34167077.4 (32059328.9–36731760.2)	2,854 (2640.3–3124.5)	53,303,208 (49337038.2–58172286.3)	2806.6 (2595–3073.3)	−0.06412 (−0.06918 to −0.05906)
Middle SDI	52,455,773 (48520200.7–57425429.2)	3076.7 (2832.5–3,375)	74250557.7 (67463856.7–82,551,232)	3065.4 (2824.2–3,361)	−0.01185 (−0.01491 to −0.00879)
North Africa and Middle East	9410291.8 (8808459.2–10133348.3)	2654.9 (2463.8–2911.4)	16004733.7 (14782735–17580716.7)	2603.4 (2414.1–2,851)	−0.07132 (−0.07559 to −0.06705)
Oceania	209991.8 (195645.6–227962.2)	3105.4 (2870.9–3393.8)	438476.9 (407848.1–478299.3)	3105.6 (2870.9–3394.1)	−0.00071 (−0.00103 to −0.00038)
South Asia	31585050.3 (29542690.8–34010815.9)	2,788 (2582–3057.2)	50329486.7 (46505547–55312434.1)	2790.9 (2585.2–3060.8)	−0.00758 (−0.01407 to −0.00109)
Southeast Asia	15360433.4 (14160967.9–16862265.3)	3306.6 (3030.1–3674.2)	22859464.6 (20690463.1–25,678,905)	3304.7 (3017.4–3,664)	−0.00097 (−0.00205 to 0.00011)
Southern Latin America	1886546.5 (1796846.1–1989851.4)	3,731 (3555.9–3,934)	2278257.3 (2176210.1–2400435.4)	3727.2 (3552.6–3929.9)	−0.00255 (−0.00284 to −0.00225)
Southern Sub-Saharan Africa	1221326.6 (1136650.9–1325291.2)	2376.2 (2186.1–2624.8)	1865305.3 (1717455.7–2,062,063)	2378.4 (2188.5–2625.6)	0.00291 (0.00141 to 0.00441)
Tropical Latin America	5804833.5 (5448977–6242297.2)	3,791 (3540.9–4088.1)	8431369.4 (7770874.5–9210255.3)	3794.2 (3543.6–4091.4)	−0.00017 (−0.00279 to 0.00246)
Western Europe	15668140.7 (14977309.9–16480486.6)	4648.5 (4433.2–4873.1)	16975348.6 (16228752.6–17873278.1)	4630.1 (4414.5–4855.7)	−0.02055 (−0.03131 to −0.0098)
Western Sub-Saharan Africa	4598202.3 (4301953.4–4,931,548)	2426.2 (2233.1–2664.5)	11654373.9 (10893384.8–12542562.1)	2427.3 (2235.7–2667.7)	0.00492 (0.00267 to 0.00718)

### Regional level

Marked regional disparities in the global burden of dermatitis were observed, closely associated with SDI levels. In 2021, the ASPR was highest in high-SDI regions, reaching 4,411.7 per 100,000 (95% UI: 4,200.5–4,653.0), and lowest in low-SDI regions, at 2,480.4 per 100,000 (95% UI: 2,288.6–2,713.9) (Table 1; Figure 1). Temporal trends in ASPR across SDI quintiles reflected differences in the stage of epidemiological transition. For instance, in low-middle SDI regions, despite a decreasing ASPR (EAPC = −0.06412; 95% CI: −0.06918 to −0.05906), the number of prevalent cases increased from 34.2 million in 1990 (95% UI: 32.1–36.7 million) to 53.3 million in 2021 (95% UI: 49.3–58.2 million), potentially reflecting improved diagnosis due to strengthened healthcare access and reporting (Table 1; Supplementary Table S1; Figure 1).

In contrast, high-middle SDI regions experienced the most notable increase in ASPR, with an EAPC of 0.05827 (95% CI: 0.03600–0.08055), although the ASPR slightly decreased from 3,391.3 per 100,000 in 1990 (95% UI: 3,183.3–3,656.0) to 3,386.5 per 100,000 in 2021 (95% UI: 3,171.3–3,665.8), indicating a sustained rising burden in this SDI tier. Similarly, ASDR varied significantly by SDI level. High-SDI regions exhibited the highest burden (ASDR = 167.9 per 100,000; 95% UI: 94.4–269.8), while low-SDI regions had the lowest (ASDR = 79.7 per 100,000; 95% UI: 48.2–124.9). Across GBD regions, substantial variability was also noted. For ASPR, Central Asia and High-income Asia Pacific recorded the highest rates, at 5,750.7 per 100,000 (95% UI: 5,434.4–6,120.4) and 5,130.1 per 100,000 (95% UI: 4,928.4–5,365.0), respectively—both exceeding 5,000 cases per 100,000 population. In contrast, Central Sub-Saharan Africa had the lowest ASPR at 2,228.9 per 100,000 (95% UI: 2,048.4–2,453.1). From 1990 to 2021, Eastern Europe showed the greatest increase in ASPR (EAPC = 0.1287; 95% CI: 0.10037–0.15704), while High-income North America experienced the steepest decline (EAPC = −0.17562; 95% CI: −0.24725 to −0.10394) (Table 1; Supplementary Table S1; Supplementary Figure S1).

**Figure 2:**
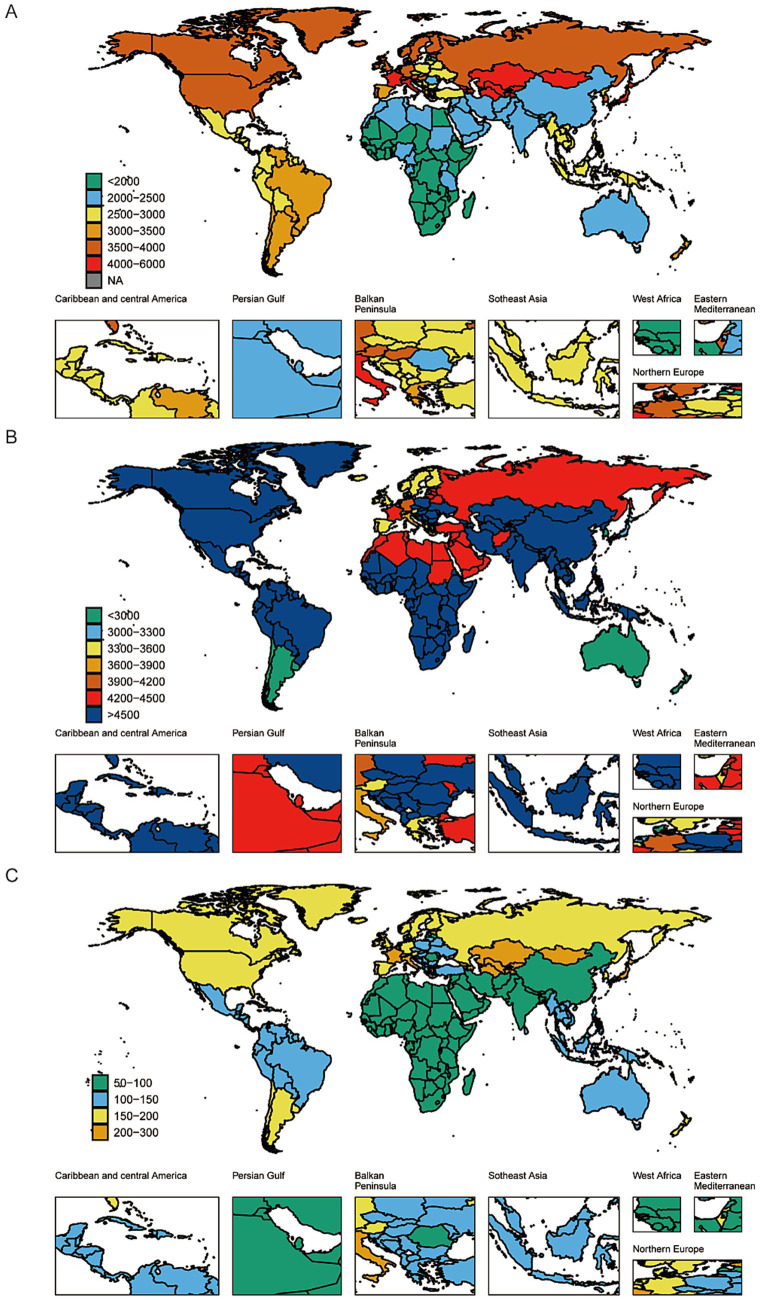
The global disease burden of dermatitis for both sexes in 204 countries and territories: **(A)** prevalence rate, **(B)** incidence rate, **(C)** DALYs rate.

Regarding ASIR, Southeast Asia and High-income North America had the highest rates, at 5,723.2 per 100,000 (95% UI: 4,955.4–6,723.0) and 5,662.1 per 100,000 (95% UI: 5,015.1–6,424.4), respectively. The lowest ASIR was recorded in Australasia at 2,793.7 per 100,000 (95% UI: 2,580.4–3,018.0). Between 1990 and 2021, Southern Sub-Saharan Africa was the only region showing a significantly increasing ASIR (EAPC = 0.0058; 95% CI: 0.00371–0.00790), whereas High-income North America had the most substantial decrease (EAPC = −0.28593; 95% CI: −0.43724 to −0.13439) (Table 1; Supplementary Figure S1). In terms of ASDR, Central Asia and High-income Asia Pacific bore the highest burden, reporting 225.6 (95% UI: 125.5–366.8) and 212.9 per 100,000 (95% UI: 112.2–349.7), respectively. By contrast, Central, Eastern, and Southern Sub-Saharan Africa had the lowest ASDRs, with rates of 69.6 (95% UI: 42.2–107.2), 73.1 (95% UI: 44.6–113.5), and 74.3 per 100,000 (95% UI: 45.4–114.6), respectively (Table 1; Supplementary Table S1; Figure 2).

Between 1990 and 2021, Eastern Europe exhibited the most notable increase in ASDR (EAPC = 0.16799; 95% CI: 0.13325–0.20275), while High-income North America experienced the most pronounced decline (EAPC = −0.14536; 95% CI: −0.18903 to −0.10167) (Table 1; Supplementary Table S1; Supplementary Figure S1).

**Figure 3:**
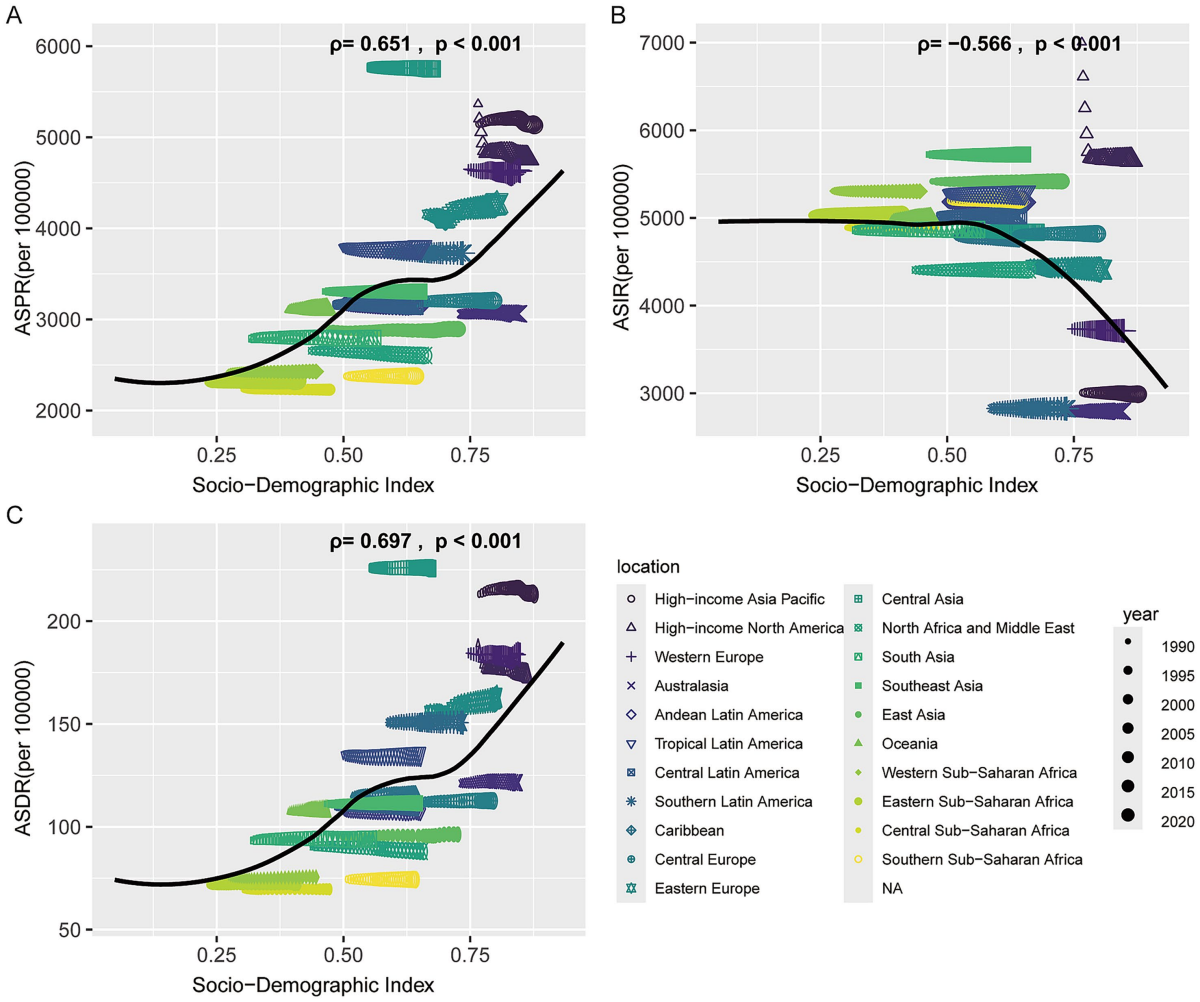
Association between the age-standardized rates of dermatitis and Socio-demographic Index. **(A)** Age-standardized prevalence rate (ASPR). **(B)** Age-standardized incidence rate (ASIR). **(C)** Age-standardized disability-adjusted life years rate (ASDR).

Correlation analysis revealed that both the ASPR and ASDR were positively correlated with the SDI (*ρ* = 0.651, *p* < 0.001; ρ = 0.697, *p* < 0.001, respectively), whereas the ASIR was negatively correlated with SDI (ρ = −0.566, *p* < 0.001). These findings suggest that regions with higher SDI levels, equipped with more robust healthcare infrastructure, systematic screening programs, and early diagnostic capabilities, are better positioned to detect dermatitis cases comprehensively and at earlier stages, thereby exhibiting relatively higher prevalence rates. In contrast, underreporting or underestimation of cases may occur in low-SDI regions due to limited healthcare resources (Figure 3; Supplementary Table S2).

In 2021, the burden of dermatitis varied markedly across sex, age groups, and SDI regions. Overall, the majority of cases were concentrated among children and adolescents, particularly in the <5 to 14-year age groups, with the number of cases gradually declining with increasing age. Across all age groups, males consistently exhibited a higher number of cases than females, with the most pronounced sex difference observed in the 15–44-year age group. A similar pattern was observed in the ASIR, which was generally higher in males than in females, especially between the ages of 20 and 44 years.

**Figure 4:**
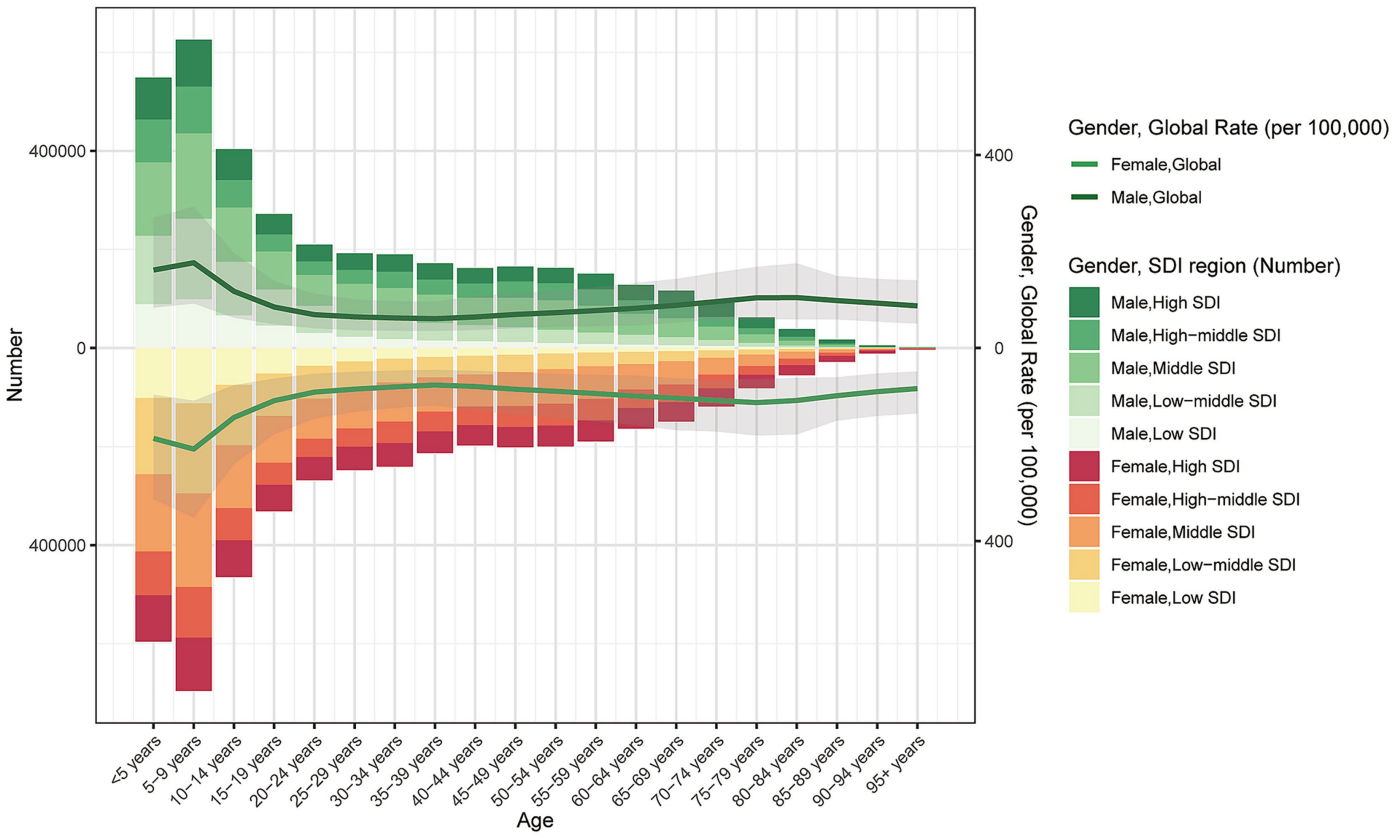
The age-specific numbers and ASDRs of dermatitis by SDI regions in 2021.

In terms of SDI stratification, males in high-SDI regions consistently accounted for the majority of cases across all age groups. However, in middle- and low-SDI regions, the number of female cases increased rapidly after the age of 40, indicating a relatively higher dermatitis burden among middle-aged and older women in developing regions. Moreover, ASIR across age showed a distinct “U-shaped” pattern in both sexes—high in early childhood, relatively stable during adulthood, and rising slightly in older age (Figure 4; Supplementary Figure S2). These findings highlight the substantial heterogeneity in the global distribution of dermatitis burden across demographic groups and underscore the need for tailored prevention and intervention strategies that consider differences in sex, age, and regional development level.

### National level

At the national level, significant differences in the prevalence and incidence of dermatitis were observed. France reported the highest global ASPR and ASDR in 2021, with an ASPR of 5,973.4 per 100,000 population (95% UI: 5,596.6–6,393.6) and an ASDR of 234.9 (95% UI: 128.5–384.7). Kazakhstan ranked second (ASPR: 5,767.4 [95% UI: 5,448.0–6,136.8]; ASDR: 226.3 [95% UI: 125.1–367.6]). The lowest ASPR and ASDR were observed in Rwanda and Congo (ASPR: 1,940.1 [95% UI: 1,762.2–2,153.9] and 2,036.5 [95% UI: 1,862.2–2,263.8], respectively; ASDR: 57.1 [95% UI: 35.8–85.2] and 61.2 [95% UI: 37.4–92.0]). Among 204 countries/territories, Taiwan (Province of China) showed the most significant increase in ASPR (EAPC: 0.11309; 95% CI: 0.07966 to 0.14653), while the United States had the most substantial decrease (EAPC: −0.19141; 95% CI: −0.26961 to −0.11315). Kenya experienced the greatest increase in ASDR (EAPC: 0.15752; 95% CI: 0.13512 to 0.17992), whereas the greatest decline was also in the United States (EAPC: −0.15908; 95% CI: −0.20684 to −0.1113), suggesting considerable differences in risk factor exposure, healthcare infrastructure, and prevention strategies.

In terms of ASIR in 2021, the highest was reported in the Philippines (5,968.8; 95% UI: 5,129.7–7,047.9), followed by Indonesia (5,965.7; 95% UI: 5,127.9–7,039.2), and the United States (5,763.2; 95% UI: 5,105.5–6,543.6) (Figure 2D; Supplementary Table S3). Regarding ASDR trends, Poland (EAPC: -5.25; 95% CI: −5.61 to −4.90), Hungary (EAPC: −4.64; 95% CI: −4.84 to −4.45), and China (EAPC: −4.71; 95% CI: −4.94 to −4.49) showed the most notable declines, while Saint Kitts and Nevis (EAPC: 3.42; 95% CI: 1.54–5.33), Turkmenistan (EAPC: 2.23; 95% CI: 1.72–2.74), and Zimbabwe (EAPC: 1.89; 95% CI: 1.38–2.39) exhibited increasing trends. For ASDR changes, the most substantial declines were seen in the United States (EAPC: -0.30811; 95% CI: −0.47174 to −0.14422), the Netherlands (EAPC: −0.09163; 95% CI: −0.13269 to −0.05055), and Turkmenistan (EAPC: −0.03393; 95% CI: −0.0343 to −0.03356), while Kuwait (EAPC: 0.02233; 95% CI: 0.0199 to 0.02477), Nauru (EAPC: 0.01871; 95% CI: 0.01723 to 0.02019), and Libya (EAPC: 0.01859; 95% CI: 0.0154 to 0.02178) showed increasing ASDR trends (Figure 2D; Supplementary Table S3; Supplementary Figure S1).

**Figure 5:**
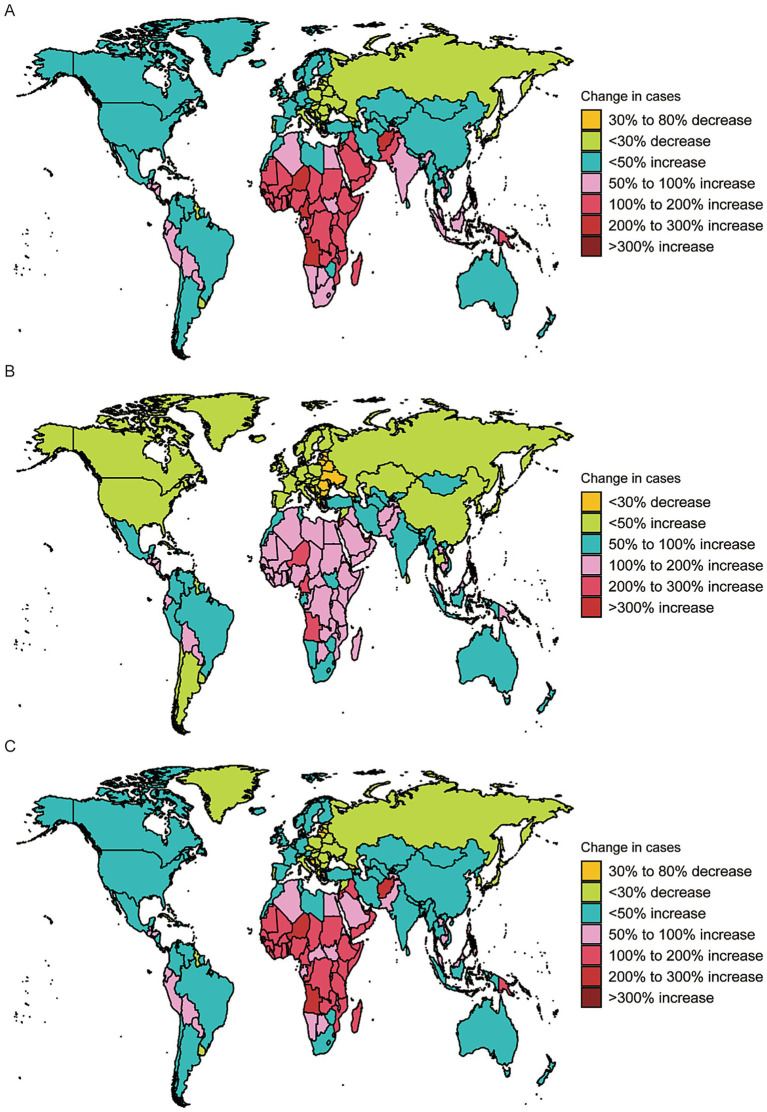
Change cases of dermatitis for both sexes in 204 countries and territories: **(A)** change prevalence cases; **(B)** change incidence cases; **(C)** change DALYs.

In addition, Qatar experienced the largest increase in the number of prevalent cases (520%), while the Russian Federation saw a 110% decrease. For incident cases, Qatar increased by 644%, whereas Georgia decreased by 29%. Regarding DALYs, Qatar had a 484% increase, and Georgia experienced a 40% decline (Figure 5).

The burden of dermatitis demonstrated distinct correlation patterns with SDI. ASIR was significantly negatively correlated with SDI (*ρ* = −0.588, *p* < 0.004), indicating that regions with higher socioeconomic development tend to have lower incidence rates, potentially due to better public health infrastructure and healthier living conditions. In contrast, ASPR was positively correlated with SDI (ρ = 0.646, *p* < 0.001), suggesting that improved disease management and extended survival may contribute to an accumulation of chronic cases in high-SDI regions. Similarly, ASDR showed a strong positive correlation with SDI (ρ = 0.686, *p* < 0.001), reflecting ongoing health loss despite advances in disease control (Supplementary Figure S3).

### Age- and sex-specific patterns

**Figure 6:**
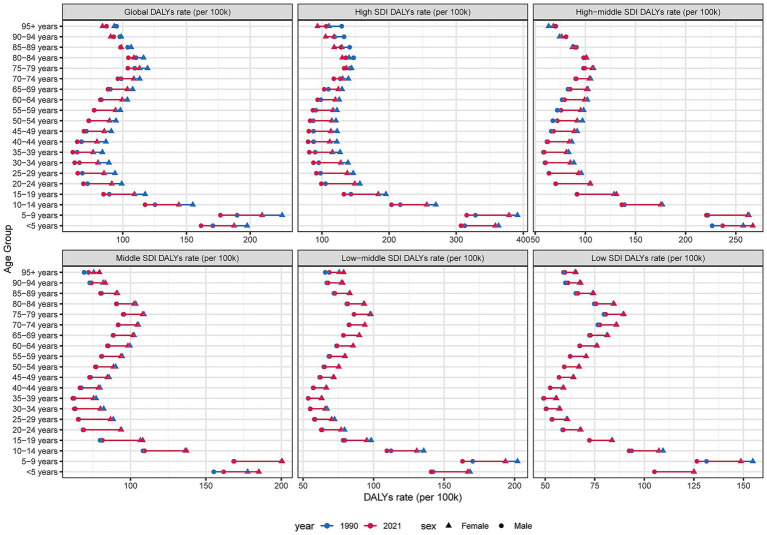
ASDR of dermatitis by sex, age group, and socio-demographic index, 1990 and 2021.

In 2021, the DALYs rate due to dermatitis showed marked disparities across SDI regions and age groups. Overall, the DALYs rate increased with age, peaking in individuals aged 85 years and older, indicating a greater health burden among the elderly. Substantial differences were observed across SDI regions: high-SDI areas maintained consistently low DALYs rates across all ages, reflecting stronger capabilities in dermatitis prevention and treatment. In contrast, middle-high and middle-SDI regions exhibited moderate DALYs rates, while low-middle and low-SDI regions had generally higher rates, especially among individuals aged over 60 years. Notably, DALYs rates among children aged 0–4 years were relatively similar across SDI categories, suggesting a globally shared risk of early childhood dermatitis. However, age-related divergence widened with increasing age. ASIR remained relatively stable, predominantly affecting individuals aged 15–64 years, with higher burden observed in low and low-middle SDI regions, and minimal sex differences. In contrast, ASPR increased across all age groups, particularly in middle and low-middle SDI countries, likely due to longer survival and improved disease management leading to cumulative prevalence. ASDR also rose with age, with the most severe health loss seen in those aged ≥65 years, especially in middle and low-middle SDI regions. Although high-SDI countries have maintained better control over all burden indicators, the overall trend highlights a continued global increase in dermatitis-related burden—particularly in low- and middle-income countries—underscoring the urgent need for early intervention and enhanced health resource allocation. Collectively, the burden of dermatitis tends to escalate with advancing age and decreasing SDI level, underscoring global disparities in dermatological care and resource distribution (Figure 6; Supplementary Figure S4).

### Inequality in health burden

**Figure 7:**
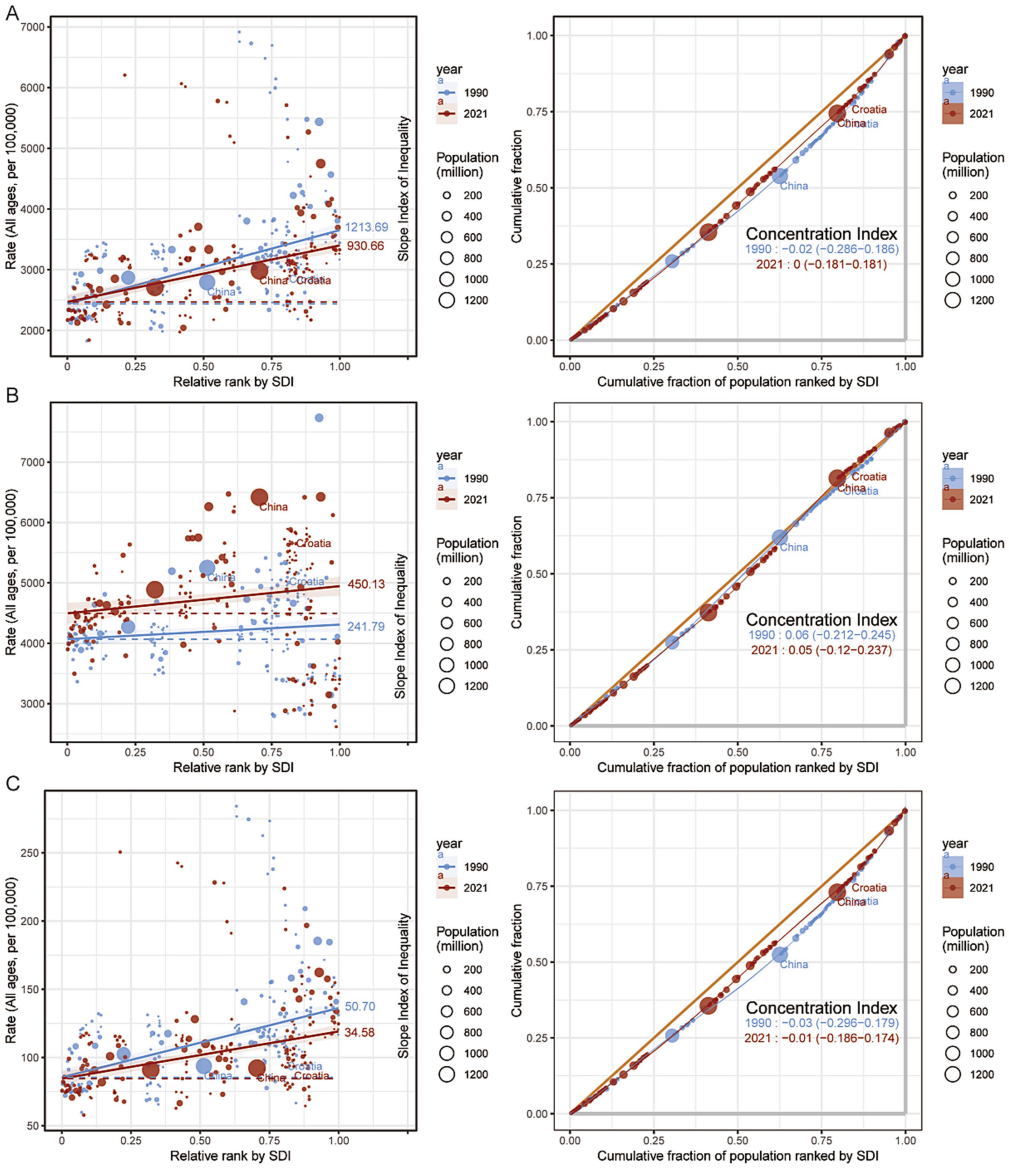
Inequality analysis of the burden of dermatitis, 1990–2021. **(A)** Prevalence rate. **(B)** Incidence rate. **(C)** Disability-adjusted life years (DALYs) attributable to dermatitis.

From 1990 to 2021, health inequalities related to dermatitis remained generally stable or showed slight improvement. Overall, ASPR was relatively higher in high-SDI countries, with a declining inequality trend; the SII decreased from 1213.98 in 1990 to 930.66 in 2021, and the CIX increased from −0.02 to 0, indicating a more balanced distribution of prevalence burden. Although the global ASIR remained relatively low, it showed a slight shift toward high-SDI countries at both time points (SII increased from 241.79 to 450.13; CIX remained at 0.05), reflecting relatively minor inequality. In contrast, ASDR exhibited moderate improvement over time, with the SII decreasing from 50.70 to 34.58 and the CIX improving from −0.03 to −0.01, suggesting a more equitable global distribution of health loss (Figure 7; Supplementary Table S4).

### Decomposition analysis

**Figure 8:**
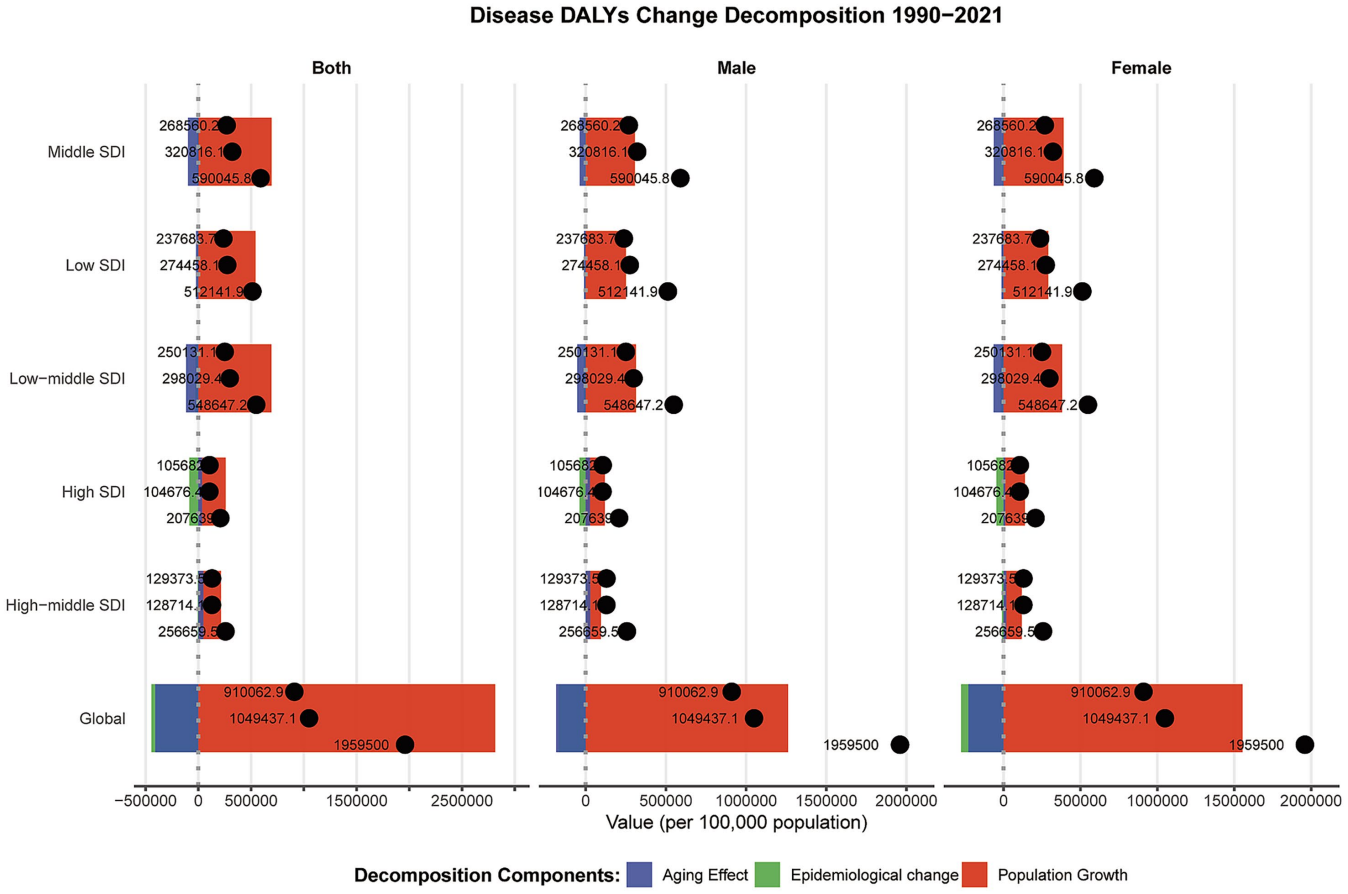
Decomposition analysis of DALYs change in dermatitis according to population-level determinants change from 1990 to 2021 at the global level and by SDI.

From 1990 to 2021, the global number of dermatitis-related DALYs increased substantially. This change can be decomposed into three major contributing factors: population aging, population growth, and epidemiological changes. Among these, population growth was the dominant driver, contributing to a 143.6% increase in DALYs, while epidemiological changes had a mitigating effect, accounting for a 22.8% reduction in the burden. Significant disparities were observed across SDI levels. In high-SDI countries, the total increase in DALYs was relatively small (207,639). Although population aging contributed positively (16.8%), improvements in epidemiological profiles led to a 41.5% reduction—the most substantial decline among all SDI groups—highlighting notable progress in dermatitis prevention and control in these settings. In middle-SDI countries, DALYs showed a marked increase, primarily driven by population growth (117.6%), while epidemiological changes accounted for a modest decrease (−13.5%), suggesting that population dynamics continue to outweigh public health improvements. In low-middle SDI countries, DALYs increased by 548,647.2, with population growth contributing 126.2%. Although both aging (−21.1%) and epidemiological change (−12.6%) had negative contributions, they failed to offset the impact of demographic expansion. For low-SDI countries, DALYs rose by 512,141.9, overwhelmingly driven by population growth (+541,211.06, +105.7%), while the effects of aging (−4.3%) and epidemiological change (−1.4%) were relatively minor. In terms of sex differences, both males and females exhibited similar trends globally. Male DALYs increased by 910,062.9 and female DALYs by 1,049,437.1, both largely driven by population growth. However, females experienced a greater benefit from epidemiological changes (−26.3% vs. –18.5%), indicating that intervention strategies targeting dermatitis among females may have been more effective in certain countries (Figure 8).

### Future projections of the global burden of dermatitis

**Figure 9:**
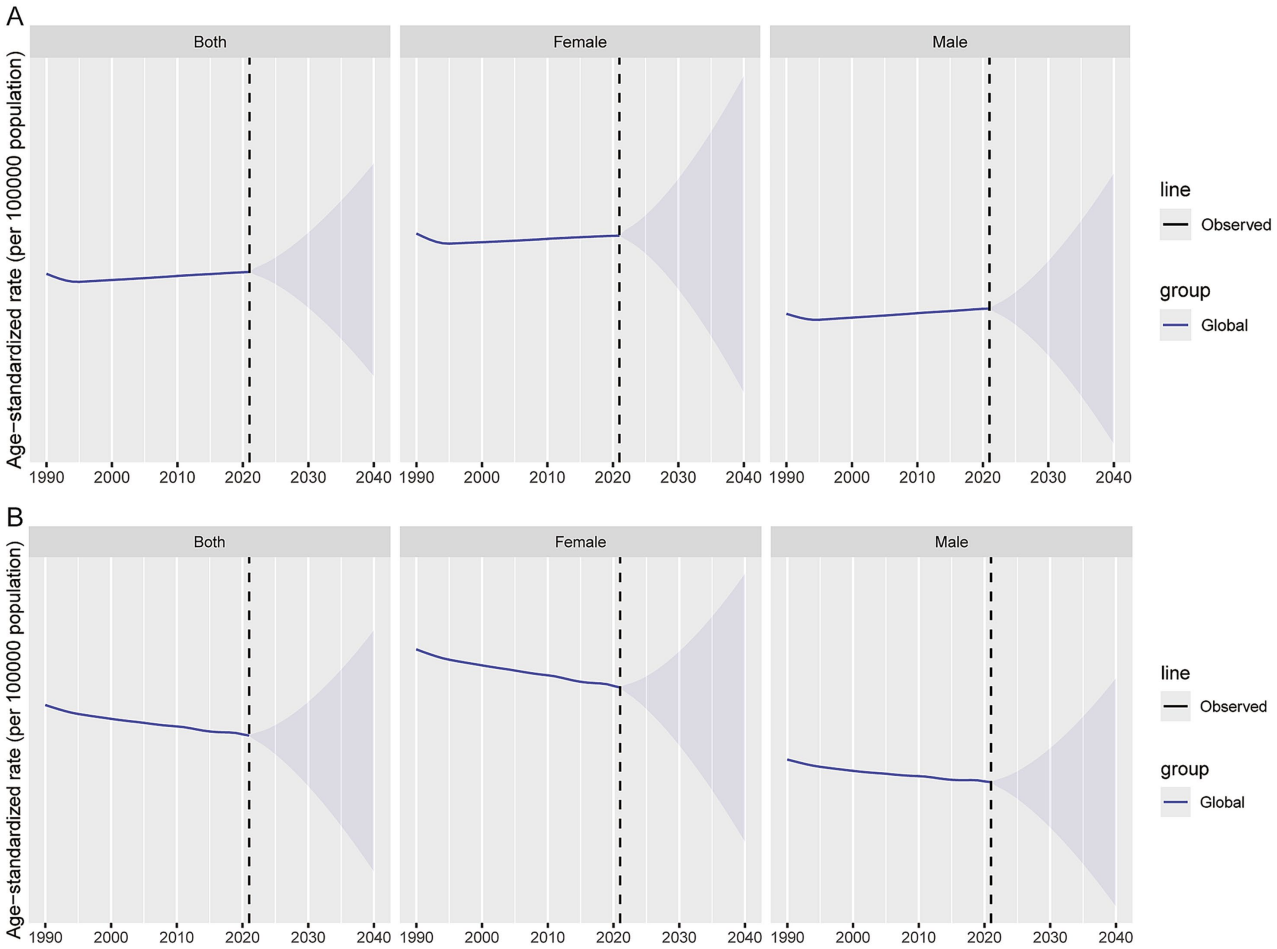
Future forecasts of global burden of dermatitis. **(A)** ASIR; **(B)** ASDR.

Exploratory projections based on the BAPC model suggest that the global burden of dermatitis may show a relatively slow overall change from 2021 to 2040. It should be emphasized that these projections are extrapolated from historical trends observed between 1990 and 2021 and are intended primarily to indicate the direction of future trends rather than provide precise numerical forecasts. In terms of incidence, the ASIR is expected to remain essentially stable. The combined ASIR for both sexes shows only a slight increase during the projection period; changes among males are minimal, whereas females may exhibit a modest upward trend. In contrast, predicted declines in mortality and disability burden are more apparent. Globally, the ASDR is projected to move downward, with similar trends in both males and females, and a relatively more pronounced decrease among females (Figure 9; Supplementary Table S5).

## Discussion

This study presents a comprehensive and systematic analysis of the global burden of dermatitis from 1990 to 2021, highlighting evolving trends in key indicators such as prevalence, ASIR, and DALYs, while also exploring disparities across regions, SDI levels, and specific populations. The findings depict a complex, multi-dimensional landscape.

Globally, the number of dermatitis cases increased substantially, from approximately 174 million in 1990 (95% UI: 163–187 million) to 241 million in 2021 (95% UI: 223–262 million). Despite the rise in absolute cases, ASPR showed a gradual decline. This pattern—rising case numbers alongside decreasing ASPR—is consistent with previous AD-focused analyses (20). ASIR showed a slight increase, suggesting modest growth in new diagnoses, whereas ASDR continued to decline, indicating a reduction in overall population-level health loss. Although GBD estimates cannot directly attribute these changes to specific clinical advances, accumulating evidence from clinical research shows that biologics targeting IL-4/IL-13 pathways and JAK inhibitors improve symptoms, quality of life, and hospitalization outcomes in patients with dermatitis (21–24). These therapeutic developments may help contextualize the observed divergence between the increasing number of patients and the decreasing age-standardized burden, although causal attribution cannot be inferred from the GBD data alone.

The burden of dermatitis varied substantially across regions and SDI levels. High-SDI countries generally reported higher ASPR but slower growth or even declining trends, potentially reflecting differences in diagnostic capabilities, healthcare accessibility, and population aging patterns (25). Longer life expectancy may also increase the number of individuals living with chronic dermatitis. High-SDI regions exhibited pronounced declines in age-standardized DALY rates, a pattern likely influenced by broader availability of healthcare resources and timely management; however, this remains an interpretation rather than a causal conclusion. In contrast, low-SDI regions reported lower ASPR but often higher and increasing ASIR and ASDR, which may reflect a combination of underdiagnosis, limited access to care, and true unmet needs in these settings. Positive correlations between SDI and both prevalence and DALYs rates support the interpretation that socioeconomic development is associated with higher reported burden—possibly due to improved case detection and longer survival with chronic disease rather than inherently higher disease risk (26). Overall, well-resourced health systems in high-SDI countries appear to mitigate disability burden, while resource constraints in low-SDI countries remain important challenges.

Environmental, occupational, and lifestyle factors have been increasingly discussed in recent literature regarding their potential influence on dermatitis burden (27). Industrialization and urbanization have elevated exposure to chemicals and pollutants. Air pollutants such as PM₂.₅ and NO₂ have been associated with AD through mechanisms described in experimental studies—including oxidative stress, AhR pathway activation, and impaired skin barrier function (28, 29). Climate change and extreme weather events may also impact dermatitis patterns: wildfire smoke and industrial emissions increase particulate matter levels, and floods can exacerbate exposure to chemicals and microbes, which have been implicated in contact dermatitis (30–32). Hot and dry climates may weaken the epidermal barrier, increasing susceptibility to irritants and allergens (33, 34). Lifestyle shifts associated with urbanization, including dietary monotony and increased exposure to aeroallergens, have also been linked to dermatitis risk (35). Occupational factors remain critical; “wet work” in construction, healthcare, and cleaning industries is a well-established risk factor for irritant and allergic contact dermatitis (36). Contact dermatitis involves a type IV delayed hypersensitivity reaction, in which exogenous haptens bind to endogenous proteins and activate antigen-specific T cell responses, amplifying inflammation with keratinocyte participation (37). Additionally, obesity has been associated with dermatitis through alterations in skin microbiota and systemic inflammation, as inflammatory adipokines may promote Th2-skewed immune responses and impair barrier integrity (38). These mechanistic explanations are supported by experimental and clinical evidence but should be interpreted as contextual biological insights rather than direct causal drivers of GBD-level epidemiological changes.

This study also provides novel insights beyond existing GBD-based dermatitis analyses. Earlier investigations often focused on specific subtypes—primarily atopic dermatitis—or were limited to selected regions. In contrast, by integrating all major dermatitis subtypes, our study reveals broader and more nuanced epidemiological patterns. The increase in global case numbers alongside relatively stable ASIR and slightly declining ASPR suggests that population growth is a primary contributor to rising case counts. DALYs rates increased markedly with age, peaking in individuals aged ≥85 years, and were highest in low and low-middle SDI regions. These findings differ from previous AD-centered research, which emphasized disease burden in young children in high-SDI countries (e.g., AD prevalence of 2–3% among children aged 0–4 years). Our study underscores the substantial burden among older adults and populations in lower-SDI settings, thereby expanding the current understanding of global dermatitis distribution.

Dermatitis prevalence varies by age and sex. Incidence and case numbers are highest in early childhood (especially ages 0–5) and adolescence, reflecting known developmental differences in skin barrier maturation and immune regulation. Dermatitis-related DALYs increase substantially with age and peak in individuals aged ≥85 years, likely due to age-related barrier decline and comorbidities (39). These age–sex patterns are heterogeneous because the aggregated GBD category “dermatitis” encompasses multiple subtypes with distinct epidemiology. Atopic dermatitis (AD) predominates in childhood and frequently shows a female bias in adults in many populations, whereas contact dermatitis and seborrheic dermatitis exhibit different age distributions and often contribute to male-dominant patterns in global estimates. Consequently, when these subtypes are aggregated across age bands, the composite age–sex curves reflect the weighted sum of individual subtype patterns rather than any single disease entity. This explains why the combined dermatitis burden appears male-dominant overall, even though adult AD alone is often female-dominant. Overall, skin disease burden is particularly high among children, adolescents, and the elderly. Decomposition analysis suggests that population growth is the main driver of rising global DALYs, especially in low-SDI countries where rapid demographic expansion amplifies this effect. In high-SDI regions, improvements in healthcare access, early diagnosis, and preventive interventions appear to partially offset burden increases, although this remains an interpretation rather than causal inference.

Forecasting analysis suggests that global dermatitis incidence and case numbers may continue to rise in the coming decades, driven largely by population aging and demographic patterns. By contrast, age-standardized DALY rates are projected to decline modestly, consistent with the potential influence of improved therapies and increased healthcare investment. Previous research has projected that AD prevalence and DALYs among older adults may continue rising through 2050, while ASDR may gradually decrease (40, 41). The BAPC model used in this study similarly indicates relative stability in incidence trends and a potential decline in future age-standardized DALYs. However, because BAPC forecasts rely on historical trends and do not account for structural changes—such as widespread adoption of biologics, shifts in diagnostic practices, or post-COVID healthcare dynamics—these projections should be interpreted as exploratory, scenario-based extensions rather than precise predictions.

## Conclusion

Although substantial progress has been made in reducing the health losses caused by dermatitis, the rising number of cases and increasing incidence remain pressing global challenges. Significant disparities persist across regions, sexes, age groups, and SDI levels. The underlying environmental, lifestyle, biological, and healthcare-related factors driving these inequalities call for enhanced international and governmental cooperation. There is an urgent need to develop more targeted and equitable prevention and control strategies, with special attention to under-resourced settings and vulnerable populations, to effectively reduce the global burden of dermatitis and promote health equity. Future research should further investigate the specific drivers of increasing incidence, as well as the unique health risks faced by women and the elderly in low- and middle-SDI countries, in order to inform the development of more effective, evidence-based interventions.

### Limitations

This study has several limitations that should be acknowledged. First, GBD estimates rely on heterogeneous primary data sources across countries, including claims databases, hospital records, surveys, and sentinel systems, where variations in case definitions, diagnostic practices, and reporting quality may introduce inconsistencies. Second, in regions with sparse or low-quality data, GBD employs modeling approaches and statistical imputation, which may introduce uncertainty and reduce the direct interpretability of estimates. Third, the severity distributions and disability weights used to calculate DALYs are derived from standardized global surveys and may not fully capture cultural or regional differences in how dermatitis affects quality of life. Fourth, time-varying shifts in coding practices, health-seeking behavior, and case detection—particularly relevant to dermatitis, which is often managed in primary care and seldom systematically recorded—may bias temporal trends. Finally, this is an ecological analysis based on aggregated national-level estimates; individual-level causal inference cannot be drawn, and ecological fallacy remains a possibility. These limitations underscore that the GBD results should be interpreted as modeled estimates reflecting the best available data rather than direct observations.

## Data Availability

The original contributions presented in the study are included in the article/Supplementary material, further inquiries can be directed to the corresponding authors.
