# Supplementary material for: Trends and projections of dermatitis burden (1990–2040): a 2021 global burden of disease analysis
Source: Front Med (Lausanne). 2026 Jan 28;13:1696683. doi: 10.3389/fmed.2026.1696683 (PMC12891110; doi:10.3389/fmed.2026.1696683)
Supplement: Supplementary file 1 [file Table_1.DOCX]

Supplementary Table S1. Global and regional trends in Dermatitis burden: incidence and disability-adjusted life years (1990–2021).

| **Incidence** |  |  |  |  |  |
| --- | --- | --- | --- | --- | --- |
| Andean Latin America | 1691894.5 (1486770.7-1941851.2) | 5183.7 (4549.1-5970.7) | 3437205.7 (2993523.6-3953387.8) | 5178.9 (4545.5-5967.2) | -0.00359 (-0.0047 to -0.00248) |
| Australasia | 579727 (535338.3-628739.9) | 2791.7 (2581.9-3014.4) | 887869.1 (816541.6-969609) | 2793.7 (2580.4-3018) | 0.00225 (1e-04 to 0.0044) |
| Caribbean | 1580951.4 (1394038.7-1825075) | 4799.1 (4218-5535.1) | 2390508.4 (2092424.1-2784208.3) | 4799.2 (4218.1-5534.8) | -0.00055 (-0.00071 to -4e-04) |
| Central Asia | 3051283.4 (2652635.4-3539436.9) | 4849.9 (4205.6-5682.4) | 4585911.5 (3967492-5410751) | 4835.4 (4194.7-5662.5) | -0.00879 (-0.00985 to -0.00774) |
| Central Europe | 6474302.7 (5554702.1-7672366.9) | 4816.3 (4157.3-5672) | 6853491.3 (5795279-8371907.1) | 4818.4 (4159.3-5672.9) | 0.002 (0.00125 to 0.00276) |
| Central Latin America | 6979698.8 (6146980.5-7995318.2) | 5019.7 (4405.3-5841.1) | 13115663.8 (11472523.5-15301146.3) | 5025.6 (4411-5848.7) | 0.00221 (0.00176 to 0.00267) |
| Central Sub-Saharan Africa | 2201697.3 (1973355.2-2455115.1) | 4888.7 (4362.6-5530.7) | 5722152.8 (5120402.1-6377668.3) | 4889.6 (4363.4-5533) | -0.00023 (-0.00086 to 4e-04) |
| East Asia | 63775342.8 (54685833.2-75070759) | 5417.2 (4674.7-6376.4) | 94240502.7 (80690231.1-112262795.4) | 5416.8 (4674.5-6377.5) | 0.00189 (0.00114 to 0.00264) |
| Eastern Europe | 10920000.2 (9460329.8-12711948.8) | 4472.8 (3883.8-5164.1) | 10650950.5 (9242908.3-12499255.5) | 4409.4 (3844.7-5058.6) | -0.02502 (-0.03121 to -0.01882) |
| Eastern Sub-Saharan Africa | 7669907.7 (6908575.5-8551401) | 5029.8 (4482-5693.2) | 18280373.7 (16421068-20461707.8) | 5040.2 (4491.1-5704.1) | 0.00487 (0.00407 to 0.00567) |
| Global | 248056086.1 (218593920-284715695.8) | 4932.4 (4334.7-5691.6) | 405021857.7 (355572501.6-467256268.8) | 4945 (4354.9-5690.3) | 0.04407 (0.03169 to 0.05645) |
| High SDI | 44653833.2 (39452987.3-51057508.7) | 4788.9 (4257.5-5432.1) | 53918913.8 (47790780.9-61546411.1) | 4467.5 (3990.5-5028) | -0.03994 (-0.10647 to 0.02664) |
| High-income Asia Pacific | 5338055.9 (4933170.1-5813579.2) | 3006.2 (2790.5-3240.5) | 5727562.6 (5250051.2-6322677.8) | 2988.7 (2775.5-3226.1) | -0.02047 (-0.02382 to -0.01711) |
| High-income North America | 21038437.3 (18308252.4-24476312) | 7002.8 (6128-8106.2) | 23374564.5 (20567761.9-26848996.8) | 5662.1 (5015.1-6424.4) | -0.28593 (-0.43724 to -0.13439) |
| High-middle SDI | 50759458.7 (44156688.5-59212058.5) | 4697.4 (4108.2-5456.6) | 72225048.7 (62468085.7-84938466.2) | 4804 (4200-5582.1) | 0.07813 (0.072 to 0.08425) |
| Low SDI | 20487304.1 (18338254.6-22957232) | 4980 (4448.1-5648.5) | 47895631 (42787292.3-53747048.7) | 4985.7 (4455.1-5654.3) | 0.00283 (0.00233 to 0.00333) |
| Low-middle SDI | 49221989.8 (43739600.6-55845155.4) | 4950.2 (4352.7-5694.2) | 92052923.6 (81345398.2-105186358.9) | 4966 (4368.3-5708.2) | 0.00777 (0.00706 to 0.00848) |
| Middle SDI | 82699683.4 (71890545.3-95848632.6) | 5313 (4624-6202.8) | 138606250.4 (119987819.3-162432950.2) | 5277 (4600.8-6140.9) | -0.02231 (-0.02269 to -0.02193) |
| North Africa and Middle East | 12735841.9 (11341286.3-14415142.7) | 4405.3 (3882-5072.6) | 26717591.6 (23556274.2-30743308.7) | 4407.9 (3885.2-5075.8) | 0.0036 (0.00269 to 0.00451) |
| Oceania | 274308 (241859.6-314793.6) | 4983.9 (4382.6-5812.3) | 621776.3 (548360.2-715316) | 4984.9 (4383.6-5814.6) | -0.00023 (-0.00052 to 5e-05) |
| South Asia | 46136326.5 (41032611.2-52352144.6) | 4859.2 (4290.3-5553.5) | 88979797.3 (78276475.4-101632132.3) | 4868.4 (4297.2-5563.9) | 0.0053 (0.00443 to 0.00617) |
| Southeast Asia | 23355479.2 (20255982.2-27359503.1) | 5723.2 (4955.8-6718.9) | 41802086.3 (35944722.7-49416031.1) | 5723.2 (4955.4-6723) | -0.00161 (-0.00264 to -0.00058) |
| Southern Latin America | 1390340.5 (1286178.3-1502468.4) | 2826.4 (2615.8-3049.1) | 1952826.2 (1800528.8-2122202.9) | 2823.9 (2613.5-3046.3) | -0.00216 (-0.00246 to -0.00187) |
| Southern Sub-Saharan Africa | 2368357.5 (2116836.9-2651806.3) | 5195.8 (4638.2-5886.9) | 4084069.4 (3634670.6-4612470.9) | 5204.1 (4644.7-5895.1) | 0.0058 (0.00371 to 0.0079) |
| Tropical Latin America | 7185211 (6341805.9-8303693.6) | 5288.2 (4610.1-6170.3) | 13011411 (11251118.1-15329497.9) | 5290.7 (4612.5-6175.4) | 0.00108 (9e-04 to 0.00127) |
| Western Europe | 14826371.4 (13375109.3-16508916.2) | 3734.9 (3399.3-4147.7) | 16641344.5 (15010977.4-18657182.2) | 3711.6 (3378.6-4120.3) | -0.02107 (-0.02244 to -0.0197) |
| Western Sub-Saharan Africa | 8482551.1 (7644733.4-9456627.4) | 5307.3 (4760.4-5970.3) | 21944198.3 (19710557.7-24450918.4) | 5303.6 (4757.7-5961.3) | -0.00219 (-0.0026 to -0.00179) |
| **Disability-adjusted life years** | | | | | |
| Andean Latin America | 43804.2 (25378.1-70398.7) | 108.5 (64.9-171.9) | 70954.4 (42483.9-111163.3) | 108.6 (64.8-170.6) | 0.01495 (0.00844 to 0.02145) |
| Australasia | 21898.3 (11943.8-35418.3) | 121.1 (65.3-197.1) | 30553.4 (16921.5-49908.9) | 121.5 (66.1-201.5) | 0.01134 (0.00036 to 0.02233) |
| Caribbean | 41616.6 (24061.3-67535.8) | 114.6 (67-184) | 52187.7 (30649.4-82833.9) | 114.4 (66.3-184) | -0.0048 (-0.00652 to -0.00308) |
| Central Asia | 177370.8 (97104.1-290177.8) | 225.8 (125.4-365) | 216094.5 (120181.4-352078.9) | 225.6 (125.5-366.8) | 0.00118 (-5e-04 to 0.00287) |
| Central Europe | 130906.6 (79832.2-204065.8) | 111.7 (66.4-178.1) | 110541.7 (69292.5-167860.7) | 112.6 (66.6-178.3) | 0.02578 (0.02113 to 0.03042) |
| Central Latin America | 195922.5 (111386.3-315078.4) | 109.9 (64.8-176.2) | 267461.9 (158942.4-426084.5) | 109.4 (64.3-175.1) | -0.01875 (-0.02542 to -0.01207) |
| Central Sub-Saharan Africa | 41849.7 (23877-67026.6) | 69.1 (41.7-105) | 103897.2 (59835.1-163798.4) | 69.6 (42.2-107.2) | 0.02689 (0.02322 to 0.03057) |
| East Asia | 1151648.7 (692677.4-1810236.3) | 96.6 (58.3-151.2) | 1365318.6 (852495.6-2058027.3) | 96.3 (58-149.8) | 0.00953 (-0.00341 to 0.02248) |
| Eastern Europe | 322677.7 (187546.3-517335.4) | 156.7 (89.2-251.8) | 268081.8 (158046.3-427655.9) | 162.8 (91.8-261.8) | 0.16799 (0.13325 to 0.20275) |
| Eastern Sub-Saharan Africa | 148096.5 (86657.3-238196.2) | 72.1 (44.1-110.8) | 329778.6 (194431.1-526932.5) | 73.1 (44.6-113.5) | 0.06211 (0.05621 to 0.06801) |
| Global | 6217601.5 (3608073.1-9988923.1) | 113.3 (66.3-181.3) | 8177101.6 (4847630.1-12991190.8) | 106.2 (62.5-169.5) | -0.18385 (-0.19122 to -0.17649) |
| High SDI | 1423958.6 (813669.7-2285877.1) | 177.4 (100.2-285.2) | 1549999 (889148.4-2482297.7) | 167.9 (94.4-269.8) | -0.13226 (-0.14882 to -0.11571) |
| High-income Asia Pacific | 316915.1 (170025.9-516944.3) | 213.1 (113.5-349.7) | 275044.4 (149973.2-442147.9) | 212.9 (112.2-349.7) | -0.01851 (-0.03791 to 0.00089) |
| High-income North America | 511350 (308044.5-804327.5) | 188.9 (111.9-301.5) | 593916.1 (349306-945526.6) | 173.1 (100.2-277.3) | -0.14536 (-0.18903 to -0.10167) |
| High-middle SDI | 1259506 (734667.6-2020774.2) | 122.3 (70.6-197.1) | 1397649.1 (837050-2180029.8) | 121.6 (70.4-196) | 0.06178 (0.03359 to 0.08998) |
| Low SDI | 449477.8 (260445.3-719088.9) | 80.5 (48.5-125.7) | 961619.6 (563496.9-1540669.8) | 79.7 (48.2-124.9) | -0.02728 (-0.0329 to -0.02167) |
| Low-middle SDI | 1230336.5 (709070.9-1967306.9) | 95.8 (56.9-152) | 1818530.8 (1083146.3-2901245.1) | 94 (56.1-149.3) | -0.07039 (-0.07718 to -0.0636) |
| Middle SDI | 1848733.8 (1092393.4-2969718) | 104.1 (62.2-164.6) | 2442780.9 (1479576.7-3789573.4) | 104 (62-164.4) | -0.00142 (-0.00562 to 0.00277) |
| North Africa and Middle East | 341903.6 (195988-554793.4) | 90.3 (53.3-143.2) | 547964.7 (324418.9-871895.9) | 88 (52.2-139.6) | -0.09395 (-0.10016 to -0.08773) |
| Oceania | 7765.8 (4439.3-12471.4) | 107.2 (63.5-171.5) | 15919.1 (9218.1-25644.4) | 107.4 (63.5-170.9) | 0.00628 (0.00427 to 0.00829) |
| South Asia | 1134246.9 (655883.1-1808035.9) | 93.3 (55.5-147.6) | 1702117.4 (1018480.6-2716601.7) | 93.9 (56-149.2) | 0.00835 (-0.00053 to 0.01722) |
| Southeast Asia | 543467.4 (320304.2-872635.2) | 111.1 (66.5-173.3) | 758305.5 (460089.6-1177145.3) | 111.4 (67-175.1) | 0.01549 (0.01323 to 0.01775) |
| Southern Latin America | 76575.4 (40539.7-127177.9) | 150.7 (80-249.9) | 89833.5 (48351.9-147325.6) | 150.6 (79.9-248.9) | -0.00291 (-0.00529 to -0.00052) |
| Southern Sub-Saharan Africa | 41160.3 (24106.7-65940.1) | 74.5 (45.5-115) | 59345.1 (35774.7-92511.4) | 74.3 (45.4-114.6) | -0.00521 (-0.00672 to -0.00371) |
| Tropical Latin America | 215234.7 (123456-344800.7) | 135.1 (78.4-216.3) | 291757.1 (172894.2-461199.7) | 135.5 (79.1-217.4) | 0.00994 (0.0048 to 0.01507) |
| Western Europe | 597790.3 (334021.9-958223) | 184.4 (101.2-299.5) | 633289.7 (355889.6-1009165.2) | 183.7 (100.6-297.6) | -0.01858 (-0.03026 to -0.00689) |
| Western Sub-Saharan Africa | 155400.6 (91880.6-250321.2) | 75 (46.3-116.7) | 394739.2 (233241.2-633681.3) | 75.5 (46.4-117.1) | 0.03267 (0.02731 to 0.03803) |
