# Supplementary material for: Trends and projections of dermatitis burden (1990–2040): a 2021 global burden of disease analysis
Source: Front Med (Lausanne). 2026 Jan 28;13:1696683. doi: 10.3389/fmed.2026.1696683 (PMC12891110; doi:10.3389/fmed.2026.1696683)
Supplement: Supplementary file 2 [file Table_2.DOCX]

Supplementary Table S2. Correlation between Dermatitis Metrics and SDI.

| Metric | Correlation Coefficient (ρ) | p-value |
| --- | --- | --- |
| ASPR | 0.651 | <0.001 |
| ASDR | 0.697 | <0.001 |
| ASIR | -0.566 | <0.001 |
