# Supplementary material for: Trends and projections of dermatitis burden (1990–2040): a 2021 global burden of disease analysis
Source: Front Med (Lausanne). 2026 Jan 28;13:1696683. doi: 10.3389/fmed.2026.1696683 (PMC12891110; doi:10.3389/fmed.2026.1696683)
Supplement: Supplementary file 3 [file Table_3.DOCX]

Supplementary Table S3: Trends in the burden of Dermatitis in 204 countries: prevalence, incidence, and disability-adjusted life years (1990–2021)

Prevalence

| **location** | **1990** | | **2021** | | **EAPC_**  **95%CI** |
| --- | --- | --- | --- | --- | --- |
|  | **Number_95% UI** | **ASR** | **Number_95% UI** | **ASR** |  |
| Afghanistan | 270391.1 (253166.1-291582.4) | 2561.8 (2372.7-2798.1) | 841654.2 (788675.2-904978.3) | 2558.7 (2368.8-2794.1) | -0.0039 (-0.00436 to -0.00345) |
| Albania | 107562.8 (99477.4-116675.1) | 3168.8 (2922.6-3481) | 79596.8 (71881.2-89927.6) | 3180.5 (2934.4-3488.3) | 0.00648 (-0.00079 to 0.01374) |
| Algeria | 681301.9 (629875.1-741614.9) | 2553.4 (2345.8-2810.8) | 1123787.7 (1033653.4-1237847.5) | 2552 (2345-2809.1) | -0.01159 (-0.01693 to -0.00625) |
| American Samoa | 1539.9 (1432.5-1676.1) | 3107.9 (2873.6-3396.7) | 1548.2 (1426.7-1700) | 3108.9 (2874.1-3397.6) | 0.00328 (0.0021 to 0.00447) |
| Andorra | 1871.4 (1788.2-1968) | 4058.9 (3877.4-4257.4) | 2658 (2536-2795.2) | 4072.8 (3889.9-4271.4) | 0.01122 (0.0086 to 0.01384) |
| Angola | 226772.9 (211077.3-244605.5) | 2228.7 (2047.7-2451.1) | 734250 (684288.5-791884.7) | 2236 (2054.6-2461.3) | 0.01036 (0.00947 to 0.01124) |
| Antigua and Barbuda | 1956.8 (1822.9-2117.2) | 3245.2 (3019.3-3518.9) | 2773.8 (2552.1-3058.6) | 3239.5 (3013.2-3512.4) | -0.00903 (-0.01146 to -0.0066) |
| Argentina | 1267917.9 (1207956.9-1337671.9) | 3731 (3555.9-3934) | 1555900.2 (1485141.1-1640099) | 3728.3 (3553.5-3931.1) | -0.0022 (-0.0024 to -0.00199) |
| Armenia | 202380.5 (191195.5-214808.4) | 5768.1 (5449.2-6137.2) | 152593.2 (143276.6-164163.3) | 5752.1 (5435.6-6121.7) | -0.01174 (-0.01499 to -0.00848) |
| Australia | 442542 (419468.1-468428.3) | 2884.2 (2726.3-3054.8) | 637503.5 (604927.3-672051.1) | 2882.8 (2727.3-3060.5) | 0.00493 (-0.00379 to 0.01365) |
| Austria | 271370.6 (260062.1-284213.8) | 4077.9 (3895.3-4278.1) | 297307.6 (284693.3-311789.8) | 4072.8 (3890.4-4272.3) | -0.00258 (-0.00396 to -0.00119) |
| Azerbaijan | 450093.6 (425266.6-477901.4) | 5773.9 (5454.1-6143.2) | 545980.9 (514119.4-584409.2) | 5731.7 (5416.3-6100) | -0.02792 (-0.03007 to -0.02576) |
| Bahamas | 8230.6 (7668.7-8907) | 3243.8 (3018-3518.2) | 12058.7 (11135.4-13233.7) | 3244 (3018.2-3517.9) | 0.00036 (0.00019 to 0.00054) |
| Bahrain | 12463.3 (11541-13689.6) | 2537.8 (2350.2-2767.5) | 35464.4 (32197.9-40088.1) | 2529.9 (2343.1-2760.6) | -0.0138 (-0.01584 to -0.01176) |
| Bangladesh | 3192606.2 (2987808.2-3411244.5) | 2704.9 (2504-2941.2) | 4399436.8 (4055386.3-4794217.8) | 2713.5 (2512.4-2950.1) | 0.01369 (0.01194 to 0.01545) |
| Barbados | 8080.8 (7502.5-8802.1) | 3244.6 (3018.5-3519.5) | 9439.6 (8631.3-10500.9) | 3239.9 (3013.8-3514) | -0.00468 (-0.00493 to -0.00444) |
| Belarus | 324961.5 (299913.6-355585.3) | 3241.8 (3010.7-3513.4) | 276043 (250637.1-306574.8) | 3228.2 (2998.7-3498.4) | -0.0107 (-0.01177 to -0.00963) |
| Belgium | 350114.6 (335762.7-367025.6) | 4076.7 (3894-4276.7) | 393555.7 (377840.7-412582) | 4078.6 (3895.5-4278.4) | 0.00207 (0.0016 to 0.00253) |
| Belize | 6382.6 (5983.8-6830.3) | 3234.8 (3009.2-3507.3) | 13626.2 (12665.3-14811.3) | 3238 (3011.9-3509.8) | 0.00375 (0.00352 to 0.00399) |
| Benin | 113854.1 (106829-121936.3) | 2344.4 (2166.1-2569) | 313684 (293485.6-338353.6) | 2343.1 (2164.5-2566) | -0.00144 (-0.00195 to -0.00093) |
| Bermuda | 1833.5 (1688.2-2016.1) | 3242.9 (3017.4-3517.5) | 2023.5 (1841.8-2266.1) | 3239.9 (3014.2-3514) | -0.00352 (-0.00458 to -0.00246) |
| Bhutan | 17896.6 (16728.4-19173.6) | 2703.2 (2500.1-2938.4) | 19560.3 (17946.4-21494.3) | 2707 (2504.2-2943.1) | 0.00333 (0.00266 to 0.00401) |
| Bolivia (Plurinational State of) | 202762 (189075.7-219154) | 3132.4 (2889.1-3438.7) | 365133.9 (336517.8-401191.1) | 3129 (2885.9-3434.6) | -0.00382 (-0.00433 to -0.00331) |
| Bosnia and Herzegovina | 136919.7 (125541.7-150980.5) | 3189 (2942.4-3501.6) | 98421 (88034.7-111931.3) | 3186.3 (2939.3-3498.9) | -0.01011 (-0.01461 to -0.0056) |
| Botswana | 30358.2 (28315.9-32699.9) | 2289.2 (2110.8-2515.2) | 52959.8 (48579.9-58440.4) | 2284.8 (2108.1-2508.7) | -0.00568 (-0.0066 to -0.00476) |
| Brazil | 5647632.5 (5302269.5-6073397.3) | 3792.9 (3539.5-4089.8) | 8170482.6 (7529657-8932878.6) | 3796 (3542-4093.3) | -0.00055 (-0.00334 to 0.00223) |
| Brunei Darussalam | 12387.5 (11781.1-13082.3) | 4334.9 (4136.9-4570.4) | 17109.9 (16308.7-18015.8) | 4334.3 (4137.3-4569.6) | -0.00459 (-0.00964 to 0.00047) |
| Bulgaria | 264142.7 (239145.4-295537.7) | 3192.2 (2944.4-3504.8) | 203406.7 (181807-231097.6) | 3181.7 (2935.4-3494.3) | -0.01315 (-0.01485 to -0.01144) |
| Burkina Faso | 224071.3 (210118.1-240051.7) | 2346 (2167.7-2570.3) | 530005 (496370.6-569956.5) | 2345.3 (2166.4-2569.2) | -0.0013 (-0.00143 to -0.00118) |
| Burundi | 121734.9 (113816.4-131211.8) | 2235.8 (2067.5-2454) | 288737.6 (269273.9-311648.9) | 2228.5 (2060-2447.6) | -0.01289 (-0.0139 to -0.01187) |
| Cabo Verde | 8412.3 (7841.3-9063.5) | 2351.6 (2172.4-2577.6) | 12772.1 (11727.5-14002) | 2339.9 (2161.2-2561.9) | -0.01737 (-0.01789 to -0.01685) |
| Cambodia | 340410.7 (316976.4-369289.6) | 3242.2 (2968.8-3595.2) | 545023.7 (499038.3-604947) | 3218.3 (2948.5-3566.7) | -0.02226 (-0.02389 to -0.02063) |
| Cameroon | 270671.9 (253701.7-289792.7) | 2554.9 (2372.9-2779.3) | 815419.1 (763206.1-871987.8) | 2551.1 (2367.1-2770.8) | 0.01069 (0.00281 to 0.01858) |
| Canada | 1119827.2 (1053626.8-1200698.7) | 4233.9 (3987.7-4518.9) | 1559865.9 (1459025.7-1679934.8) | 4233.9 (3987.4-4517.9) | 0.00089 (-9e-05 to 0.00187) |
| Central African Republic | 60465.4 (56221.4-65437.4) | 2233.7 (2052.9-2458.8) | 121215.2 (112597-131358.2) | 2234.7 (2052.7-2459.8) | 0.00096 (0.00014 to 0.00178) |
| Chad | 141078.9 (132425.3-151124.4) | 2345.2 (2166.7-2569) | 414960.7 (389232.2-445489.8) | 2338.1 (2161.2-2559.4) | -0.01127 (-0.01174 to -0.01081) |
| Chile | 505534.5 (480615.3-533641.7) | 3731.2 (3556.3-3934.2) | 610346.1 (582607.3-642126.1) | 3724.4 (3550.2-3926.7) | -0.00388 (-0.00457 to -0.00319) |
| China | 32869285.6 (29722666-36564146.8) | 2887.7 (2617.3-3196.5) | 42423944.2 (37732817.8-48277948.1) | 2877 (2617-3183.7) | 0.00083 (-0.01046 to 0.01213) |
| Colombia | 1045365.3 (975191.2-1128113.4) | 3174.3 (2951.5-3441.8) | 1501677.4 (1384481.9-1644662.8) | 3173.6 (2950.8-3441) | -0.00086 (-0.00121 to -0.00051) |
| Comoros | 10150.9 (9471.5-10928.9) | 2231.7 (2063.2-2450.7) | 16283.5 (15075.3-17863.3) | 2231.1 (2062.8-2449.5) | -0.00138 (-0.0018 to -0.00096) |
| Congo | 47694 (43913.7-52042.3) | 2040.5 (1864.9-2268.1) | 106500.9 (97418.1-117221.8) | 2036.5 (1862.2-2263.8) | -0.01456 (-0.01895 to -0.01016) |
| Cook Islands | 597.9 (555.6-650.7) | 3104.1 (2870-3392.7) | 547.9 (502-606.5) | 3122.7 (2887.1-3412.8) | 0.02179 (0.01942 to 0.02417) |
| Costa Rica | 98926.1 (92448.9-106587) | 3171.3 (2948.6-3438.2) | 145843.4 (134202.5-159663.8) | 3176.5 (2954.3-3444.2) | 0.00642 (0.00593 to 0.00692) |
| Croatia | 146139.5 (132941.8-162578) | 3192 (2944.7-3507.4) | 125567 (112251-142911) | 3184.7 (2937.9-3497.3) | -0.00857 (-0.00941 to -0.00774) |
| Cuba | 339282.6 (314877-370621.5) | 3234.7 (3008.6-3507.7) | 355806.4 (325725.2-395218.9) | 3234.2 (3008.3-3508.3) | -0.00064 (-0.00072 to -0.00056) |
| Cyprus | 27782.1 (25753.7-29971.3) | 3745.7 (3473.6-4043.8) | 42044.2 (39246.9-45177.5) | 3748.5 (3476.1-4047.2) | -0.00728 (-0.01356 to -0.001) |
| Czechia | 312377.9 (284692-346142.2) | 3192.6 (2945.4-3506.8) | 320882.9 (288144.6-361708) | 3181 (2934.5-3493) | -0.0133 (-0.0139 to -0.01269) |
| Côte d'Ivoire | 281781.7 (263441-303969.4) | 2333.4 (2156.3-2553.6) | 640938.3 (598820.7-694110.8) | 2332.8 (2156.1-2553.5) | -0.00253 (-0.00335 to -0.0017) |
| Democratic People's Republic of Korea | 639020.9 (589446.2-702429.2) | 3144.5 (2907.2-3440.1) | 801867.3 (730485-892586.6) | 3128.5 (2892.9-3420.1) | -0.01856 (-0.01939 to -0.01773) |
| Democratic Republic of the Congo | 848872.7 (789985.2-914894.9) | 2232.5 (2051.8-2457.7) | 1997959.3 (1855826.9-2161896.2) | 2230.8 (2049.4-2454.3) | -0.00325 (-0.00385 to -0.00265) |
| Denmark | 203446.2 (189610.7-217422.6) | 4729.1 (4379.6-5066.2) | 227212.7 (212901.1-242073.7) | 4727.2 (4378.5-5064.6) | 0.04174 (0.02294 to 0.06054) |
| Djibouti | 8920.4 (8288.2-9638.4) | 2223 (2055.1-2441.9) | 26995.8 (24918.1-29624.4) | 2218 (2050-2437.6) | -0.00825 (-0.00914 to -0.00736) |
| Dominica | 2388.9 (2234.7-2583.4) | 3240 (3013.4-3517.2) | 2105.7 (1944.6-2304.8) | 3234.4 (3009.1-3506.8) | -0.00795 (-0.00963 to -0.00628) |
| Dominican Republic | 235903.3 (220453.2-254761.4) | 3241.8 (3015.7-3514.1) | 350060 (324903.6-380618.3) | 3236.2 (3010.3-3509.6) | -0.00721 (-0.00817 to -0.00626) |
| Ecuador | 316590.9 (293830.8-343060.2) | 3157.7 (2906.7-3468.7) | 566195.5 (521309.3-620031.9) | 3157.3 (2906.8-3468.1) | 0.00279 (0.00066 to 0.00492) |
| Egypt | 1144794.2 (1054712.7-1264486.5) | 2076.4 (1890.5-2318.5) | 2158025.7 (1972178.6-2396660.8) | 2062.7 (1877-2300) | -0.04178 (-0.05479 to -0.02877) |
| El Salvador | 175736.6 (164213.1-188251.7) | 3177.8 (2955.5-3445.8) | 204468.9 (189970.8-221700.6) | 3182 (2959.8-3450.5) | 0.00402 (0.00325 to 0.00479) |
| Equatorial Guinea | 9451.3 (8811.6-10175.4) | 2236.9 (2055.3-2462.2) | 32936.2 (30425.7-35774.7) | 2223.2 (2042.7-2448.5) | -0.02425 (-0.02587 to -0.02264) |
| Eritrea | 74105.7 (69197.4-79954.9) | 2235.1 (2066.4-2453.5) | 142803.2 (132643.5-155398.2) | 2232.5 (2064.7-2450.4) | -0.004 (-0.00456 to -0.00344) |
| Estonia | 78174.2 (73771.8-83088.2) | 5408.6 (5136.2-5711.3) | 59446.4 (55605.3-63653) | 5378.2 (5108.7-5678.4) | -0.03671 (-0.04669 to -0.02673) |
| Eswatini | 18622.9 (17391.1-20013.1) | 2290.9 (2111.7-2517.7) | 25913 (23997.7-28260.1) | 2287.3 (2109.9-2513.4) | -0.00529 (-0.00661 to -0.00397) |
| Ethiopia | 1077751.1 (1008247.4-1167119.8) | 2256.6 (2063.3-2505.3) | 2319243.6 (2153699.3-2530014.7) | 2256 (2062.7-2504.4) | 0.02688 (0.01752 to 0.03625) |
| Fiji | 24012.6 (22323.8-26203.8) | 3111.4 (2876.6-3399.5) | 28660.4 (26455-31333.1) | 3112.2 (2877.9-3399.6) | 0.00038 (-0.00017 to 0.00093) |
| Finland | 177899.6 (170473.3-186691.4) | 4078.1 (3895.3-4278.3) | 186567.5 (179166.6-195730.7) | 4074.8 (3892-4274.4) | -0.00251 (-0.00267 to -0.00235) |
| France | 3163949.3 (2968704.3-3379885.3) | 5971.2 (5594.4-6391.3) | 3497610.1 (3286612.5-3736625.3) | 5973.4 (5596.6-6393.6) | -0.02737 (-0.04383 to -0.0109) |
| Gabon | 25983 (24151.5-28056.8) | 2560.6 (2365-2785.3) | 46923.5 (43592.9-51134) | 2559 (2371.6-2786.8) | -0.01325 (-0.02821 to 0.00171) |
| Gambia | 22738 (21276.8-24501.1) | 2337 (2160.1-2557.5) | 55308.5 (51603.2-59860.2) | 2341.7 (2163.5-2564) | 0.00814 (0.00684 to 0.00945) |
| Georgia | 302534.5 (284296.7-323290) | 5725.9 (5399.8-6101.1) | 186854.4 (175375.8-200335.9) | 5726.5 (5412.7-6105.9) | 0.01795 (0.00706 to 0.02885) |
| Germany | 3043290.8 (2910125-3195696.6) | 4454.1 (4265.5-4651.6) | 3154193.6 (3010250.4-3318197.3) | 4444.3 (4241.7-4656.1) | -0.01196 (-0.05723 to 0.03333) |
| Ghana | 317036 (295247.3-342847.8) | 2178.1 (1997.9-2403.3) | 725314.6 (671187.9-788884.6) | 2182.2 (2002.1-2408.6) | 0.032 (-0.00863 to 0.07263) |
| Greece | 332098.7 (311648.1-358539.4) | 3654.5 (3427.4-3955.6) | 298629 (279693.9-319954.6) | 3678.2 (3421.5-3971.5) | 0.03251 (0.02661 to 0.03841) |
| Greenland | 2237.8 (2104.1-2393.1) | 4183.8 (3940.5-4467.6) | 2286.1 (2133.8-2457.2) | 4211.2 (3967.3-4495.5) | 0.0188 (0.01774 to 0.01986) |
| Grenada | 2949.2 (2765.6-3170.7) | 3242.5 (3016.7-3517.5) | 3208.3 (2965.2-3514.9) | 3233 (3007.5-3505.7) | -0.01201 (-0.01458 to -0.00944) |
| Guam | 4155.5 (3832.6-4573.8) | 3097.8 (2863.7-3384) | 4886 (4482-5387.9) | 3107.9 (2873.3-3394.5) | 0.00722 (0.00375 to 0.0107) |
| Guatemala | 288554.4 (270979.3-307562.5) | 3176.1 (2953.2-3443.4) | 502463.9 (467783.9-542945.8) | 3178.4 (2956.5-3446.1) | 0.00232 (0.00217 to 0.00246) |
| Guinea | 140477.2 (131830.1-151155.5) | 2342.9 (2165-2566.4) | 313202.6 (293026.4-337218.6) | 2343.2 (2165.4-2566.3) | 0.00047 (-0.00048 to 0.00142) |
| Guinea-Bissau | 23507.2 (22016.1-25244.8) | 2345.2 (2166.9-2568.7) | 47711 (44549-51474.1) | 2345.1 (2166.5-2568.7) | -0.00017 (-0.00039 to 5e-05) |
| Guyana | 25541.2 (23865-27591.5) | 3241 (3015.2-3513.9) | 24473.9 (22730.9-26676.3) | 3240.4 (3014.5-3513.9) | -0.00028 (-0.00153 to 0.00097) |
| Haiti | 215915 (202046.1-232624.6) | 3245.3 (3019.2-3517.6) | 416774.7 (388054-452040) | 3241.9 (3015.6-3515) | -0.00424 (-0.00457 to -0.00391) |
| Honduras | 160986.1 (151063-171711.1) | 3173.9 (2951.2-3441) | 321613.3 (299534.9-347363.5) | 3177.3 (2955.4-3445) | 0.0037 (0.00323 to 0.00417) |
| Hungary | 385501.9 (353564.6-422849.5) | 4013 (3703.7-4355.9) | 341407.4 (310218.5-379851.9) | 4000.7 (3691.9-4342.5) | -0.04318 (-0.06227 to -0.02409) |
| Iceland | 10911.7 (10162.2-11741.9) | 4511 (4206.6-4852.6) | 13691.2 (12829.1-14647.1) | 4489.5 (4192.6-4807.6) | -0.02716 (-0.0366 to -0.01772) |
| India | 24486289.4 (22842410.1-26507937.1) | 2792.5 (2583.1-3061.2) | 38336296.4 (35336140.6-42296469) | 2795.3 (2586-3064.3) | -0.01103 (-0.01961 to -0.00245) |
| Indonesia | 6162467.5 (5653355-6760703.9) | 3369.4 (3062.5-3744.5) | 9301190.5 (8337645.5-10505282.2) | 3377.6 (3058.3-3746.6) | 0.01391 (0.01016 to 0.01767) |
| Iran (Islamic Republic of) | 1715256.1 (1603949.6-1833723.4) | 2844 (2614.9-3122.1) | 2372050.3 (2151524.1-2651497.9) | 2865.6 (2626.2-3146.1) | 0.04186 (0.03385 to 0.04987) |
| Iraq | 497654.4 (467261.5-533882.9) | 2554.2 (2364.8-2788) | 1051934.7 (976861.7-1148704.1) | 2553.2 (2363.7-2786.7) | -0.00136 (-0.00186 to -0.00087) |
| Ireland | 144877.7 (138527.8-152068.2) | 4078 (3895-4278) | 178147.1 (171089.4-186896.5) | 4082 (3898.6-4281.7) | 0.00438 (0.00318 to 0.00558) |
| Israel | 209415.3 (200030.5-220041.1) | 4083.2 (3899.9-4283) | 385758.7 (369372.5-404691.2) | 4078.5 (3895.6-4278.3) | -0.00386 (-0.00392 to -0.00379) |
| Italy | 2426736.1 (2320399.2-2553348.3) | 5026.3 (4815.9-5277.8) | 2405100.9 (2294659-2529672.5) | 5019.6 (4809.7-5270.1) | -0.0151 (-0.02141 to -0.00878) |
| Jamaica | 78437.8 (73438.9-84695.8) | 3242 (3016.1-3515.4) | 87085.5 (80476.3-95521.3) | 3237.8 (3011.9-3510.4) | -0.00448 (-0.00484 to -0.00412) |
| Japan | 5746506.2 (5530090.9-6004253.4) | 5430.7 (5209.3-5681.4) | 5214750.6 (5029635.3-5443069.3) | 5437.8 (5233.2-5697.6) | -0.02435 (-0.0592 to 0.01051) |
| Jordan | 99078.1 (91928.9-107718) | 2528.8 (2325.9-2790) | 305646.1 (279489.9-338164.1) | 2524.9 (2322.7-2786.3) | -0.00966 (-0.01362 to -0.00571) |
| Kazakhstan | 982586.9 (928243.2-1042649.1) | 5774.3 (5454.5-6145.3) | 1082568.5 (1023949.4-1153576.5) | 5767.4 (5448-6136.8) | -0.0032 (-0.00479 to -0.00161) |
| Kenya | 470317.9 (436990.2-508725.4) | 2191.9 (1998.3-2437.6) | 1064558.7 (984819.4-1164283.1) | 2239.6 (2046-2489.3) | 0.0969 (0.08375 to 0.11005) |
| Kiribati | 2384.4 (2220-2594.2) | 3120.1 (2885.4-3408) | 3816.6 (3538.1-4177.5) | 3123 (2888.2-3410.1) | 0.00218 (0.00147 to 0.00289) |
| Kuwait | 44306.6 (40729.5-48828.1) | 2619.5 (2415.4-2878.1) | 110632.4 (99642.4-125004.2) | 2623.2 (2408.4-2881.6) | -0.01137 (-0.01773 to -0.005) |
| Kyrgyzstan | 289301.1 (273285-306278.4) | 5770.9 (5451.4-6140.8) | 416264.2 (393298.5-441900.4) | 5762 (5442.9-6131.2) | -0.00288 (-0.00408 to -0.00168) |
| Lao People's Democratic Republic | 137375.3 (127890.1-149555.3) | 3225.9 (2954.2-3576.7) | 235284.1 (215829.9-261136.8) | 3211.6 (2941.7-3560) | -0.01423 (-0.01553 to -0.01293) |
| Latvia | 58167.5 (51411.5-65571.1) | 2184.1 (1948.8-2434.4) | 41811.2 (36509.1-48150.9) | 2169.9 (1936.8-2418.2) | -0.02051 (-0.02331 to -0.01772) |
| Lebanon | 77954.9 (72586.1-84866.2) | 2559.8 (2370.5-2794.7) | 137274 (126042.9-151425) | 2558.6 (2368.6-2793.2) | -0.00129 (-0.00216 to -0.00043) |
| Lesotho | 35677.8 (33259.6-38373.1) | 2298.7 (2117.3-2526.1) | 42202.1 (38976.3-45972.4) | 2289.4 (2111.7-2515.4) | -0.01261 (-0.01304 to -0.01217) |
| Liberia | 57392.8 (53767.1-61706.8) | 2336.6 (2159.3-2557.9) | 125379.2 (117059.4-136228.1) | 2335.8 (2158.5-2556.9) | -0.00772 (-0.00935 to -0.00609) |
| Libya | 113034.9 (105957.9-121425.5) | 2547.2 (2358.3-2780.4) | 165409.2 (151187.8-184918.5) | 2553.4 (2364.1-2787.3) | 0.00554 (0.00462 to 0.00645) |
| Lithuania | 113740.4 (105029.4-124425.1) | 3241.4 (3010.6-3512.9) | 80967.5 (73222.7-90515.3) | 3226.8 (2997.3-3496.4) | -0.01554 (-0.01679 to -0.0143) |
| Luxembourg | 13167.1 (12606.7-13819.2) | 4076.9 (3894.1-4277.1) | 21560.7 (20642.5-22633) | 4072.7 (3890-4272.4) | -0.00348 (-0.00404 to -0.00292) |
| Madagascar | 260440.5 (243005.5-280896.2) | 2229.9 (2061.7-2448.7) | 624055.8 (579674.4-676178.6) | 2232.5 (2064-2451) | 0.00362 (0.00329 to 0.00395) |
| Malawi | 213075.2 (198895.5-229790.4) | 2233.4 (2064.9-2451.9) | 426113.2 (396257.6-459435.7) | 2235.9 (2067.2-2454.3) | 0.00478 (0.00429 to 0.00526) |
| Malaysia | 571321.3 (527459.6-627736.6) | 3211.3 (2941.1-3560.1) | 1000993.2 (910788.8-1123394.2) | 3199.2 (2929.7-3545.8) | -0.01416 (-0.01505 to -0.01328) |
| Maldives | 7377.8 (6856.9-8007.8) | 3200.6 (2929.1-3550.4) | 15366.2 (13731-17622.8) | 3144.5 (2876.1-3483.2) | -0.06667 (-0.08077 to -0.05257) |
| Mali | 213486.9 (199335.7-229745.8) | 2450.5 (2270.6-2678.5) | 593290.5 (550624.9-639329.6) | 2443 (2252.5-2678.7) | -0.01282 (-0.01504 to -0.0106) |
| Malta | 14035.3 (13441.5-14730) | 4078.6 (3896-4278.3) | 14683.8 (14092.7-15415.6) | 4069.9 (3887.8-4269.4) | -0.0069 (-0.00725 to -0.00654) |
| Marshall Islands | 1513.6 (1413.3-1631.6) | 3109.9 (2876-3398.4) | 1735.3 (1605.8-1903.7) | 3108.3 (2874.1-3396.1) | -0.0026 (-0.00335 to -0.00186) |
| Mauritania | 47935.2 (44887.2-51620.9) | 2341.5 (2163.2-2564.1) | 102610.4 (95926-110846.1) | 2341.5 (2163.5-2564.2) | 0.00043 (4e-05 to 0.00082) |
| Mauritius | 34792.4 (31812.1-38719.5) | 3213.9 (2944.3-3561.9) | 40234.4 (36279.5-45425.1) | 3213.6 (2943.9-3562) | -0.00099 (-0.0022 to 0.00021) |
| Mexico | 2594351.1 (2412586.9-2801704.7) | 3044.5 (2796.3-3344.7) | 3837212.4 (3491132.5-4249379.6) | 3016.5 (2766.3-3315.9) | -0.04209 (-0.0469 to -0.03727) |
| Micronesia (Federated States of) | 3395.4 (3163.7-3667.6) | 3110 (2876.2-3398.6) | 3168.7 (2928.3-3473) | 3110.6 (2876.4-3397.7) | -0.00068 (-0.00177 to 0.00041) |
| Monaco | 963.6 (922.9-1011.3) | 4076.3 (3893.9-4275.7) | 1236.4 (1186.1-1297) | 4085.8 (3901.9-4285.2) | 0.01139 (0.00921 to 0.01356) |
| Mongolia | 145791.2 (137736.4-154629.9) | 5752.8 (5434.3-6119.9) | 200677.1 (189923.8-213499) | 5763.9 (5445-6133.5) | 0.00529 (0.00445 to 0.00613) |
| Montenegro | 19457.4 (17863.1-21422.5) | 3189.4 (2942.7-3501.2) | 18617.7 (16761.7-20899.1) | 3183.9 (2937.2-3495.1) | -0.00521 (-0.00657 to -0.00385) |
| Morocco | 667139.8 (623801-721177.1) | 2556.5 (2367.8-2791.1) | 933555.9 (860034.7-1024850.7) | 2555.9 (2366.9-2789.9) | -0.00101 (-0.00202 to -1e-05) |
| Mozambique | 293785.7 (274456.9-316149.8) | 2236.8 (2067.1-2456) | 684260.5 (639177.2-736751.9) | 2238 (2068.9-2456.2) | 0.00168 (0.00125 to 0.00211) |
| Myanmar | 1312431.3 (1211513.8-1445363.6) | 3221.4 (2950.8-3571.4) | 1807881.6 (1651812-2012348.1) | 3224.5 (2953.9-3574.2) | 0.00216 (0.00071 to 0.00361) |
| Namibia | 32084.6 (29905.5-34549.1) | 2286.2 (2108.9-2510.8) | 54645.4 (50471.3-59779.8) | 2287.9 (2110.4-2513.5) | 0.00199 (0.00167 to 0.0023) |
| Nauru | 328.5 (306.2-356.2) | 3102.8 (2868.9-3390.2) | 347.5 (322.8-379) | 3114.8 (2879.9-3401.5) | 0.01262 (0.01186 to 0.01338) |
| Nepal | 571225.6 (534475.2-609944) | 2766.1 (2563.4-3004.3) | 855563.9 (789189.4-926700.8) | 2773.6 (2568.8-3012) | 0.01031 (0.00923 to 0.0114) |
| Netherlands | 529578.1 (506023.9-556486.2) | 4096.1 (3905.9-4297.6) | 571642.9 (549731.8-599642.6) | 4032.1 (3851.1-4222.6) | -0.03444 (-0.05003 to -0.01884) |
| New Zealand | 123393.5 (118608.3-128623.3) | 3906.2 (3751.4-4076.4) | 175000.4 (168366.6-182566.2) | 3931.4 (3777.6-4112.2) | 0.03299 (0.0251 to 0.04089) |
| Nicaragua | 132931.9 (124565.6-142023.2) | 3177.8 (2955.3-3445.8) | 209662.3 (195089.8-226985.2) | 3174.1 (2951.3-3441.6) | -0.0037 (-0.00408 to -0.00331) |
| Niger | 186687.9 (175050.5-199982.6) | 2339.3 (2161.6-2560.7) | 586441.5 (549938.8-629622.3) | 2341.4 (2163.2-2563.7) | 0.00392 (0.0032 to 0.00465) |
| Nigeria | 2187760.9 (2032604.1-2359530.1) | 2507.6 (2296.1-2767.6) | 5610514.6 (5217224.5-6062246) | 2510.6 (2300.1-2772.1) | 0.00577 (0.00472 to 0.00681) |
| Niue | 74.5 (69.3-80.7) | 3110.2 (2875.7-3396.8) | 51.5 (47.2-56.8) | 3110.1 (2875.7-3398.3) | -8e-05 (-0.00037 to 0.00022) |
| North Macedonia | 61886.8 (56864.9-68168.2) | 3187.9 (2940.7-3499.5) | 63106.1 (56687.4-71235.5) | 3174.8 (2928.9-3485) | -0.01663 (-0.01775 to -0.01551) |
| Northern Mariana Islands | 1330 (1212.6-1481.3) | 3105 (2874.6-3390.3) | 1471.6 (1345-1638.8) | 3101.6 (2869.4-3389.2) | -0.00601 (-0.01432 to 0.00231) |
| Norway | 168814.1 (160969.4-177202.2) | 4503.4 (4304.9-4734.7) | 209279.6 (199699-219498.6) | 4522.4 (4326.2-4736.5) | 0.02295 (0.00143 to 0.04447) |
| Oman | 51336.3 (47482.7-55883.5) | 2509.1 (2303.9-2755.1) | 110418.5 (100057-123709.7) | 2503.3 (2296.6-2757.2) | -0.01974 (-0.02986 to -0.00962) |
| Pakistan | 3317032.5 (3124817.1-3553211.9) | 2822.9 (2615.7-3098.5) | 6718629.2 (6266085.6-7281690) | 2827.7 (2619.5-3104) | 0.00538 (0.0053 to 0.00546) |
| Palau | 465.8 (430.1-511.6) | 3106.7 (2872.2-3393.7) | 536.6 (486.6-604.9) | 3086.4 (2854.7-3369.6) | -0.02128 (-0.02704 to -0.01552) |
| Palestine | 58081.5 (54179.8-62709.7) | 2633.7 (2431.7-2890.6) | 136466.3 (126310.7-149352.6) | 2607.1 (2404.6-2866.3) | -0.01854 (-0.02838 to -0.00871) |
| Panama | 76916.5 (71749.7-82911.6) | 3168.6 (2945.9-3434.8) | 135123.6 (125476-146677.6) | 3168.8 (2946.4-3435.2) | 0.00059 (0.00016 to 0.00102) |
| Papua New Guinea | 131693.1 (122708.6-142891.6) | 3103.8 (2869.4-3392.4) | 329686.4 (306863.8-359755.8) | 3103.3 (2868.9-3392.2) | -0.00174 (-0.00213 to -0.00135) |
| Paraguay | 157201 (148105.3-166947.1) | 3700.7 (3461.1-3973.1) | 260886.8 (243943.3-279775.6) | 3698.3 (3458.6-3970.7) | -0.00244 (-0.00263 to -0.00225) |
| Peru | 696106.4 (646474.3-755152.2) | 3206.9 (2957.7-3496.5) | 1146517.7 (1054082.3-1253719.4) | 3202.4 (2953.6-3491.5) | 0.0025 (-0.00901 to 0.014) |
| Philippines | 2111211.2 (1951518.6-2306627.4) | 3381.2 (3080-3742.3) | 3780500.7 (3445553.2-4217540.5) | 3377 (3076.4-3737.4) | -0.00428 (-0.00451 to -0.00404) |
| Poland | 1259640.1 (1152527.9-1391322.4) | 3359.9 (3097.6-3669.8) | 1230904.9 (1099930.4-1408280.4) | 3351.1 (3089.5-3658.7) | -0.00963 (-0.01023 to -0.00904) |
| Portugal | 370317.9 (355156.4-388112) | 4084.7 (3901.7-4284.7) | 343634.1 (329330.3-360522.3) | 4084.2 (3901-4284.1) | 0.00054 (-0.00018 to 0.00126) |
| Puerto Rico | 116249.8 (108229-126281.8) | 3242.2 (3015.8-3515.7) | 104959.2 (96053.3-116814.7) | 3241.1 (3014.9-3515.2) | -0.00149 (-0.00169 to -0.00128) |
| Qatar | 10438.1 (9580.5-11621.2) | 2515.2 (2330.4-2743.7) | 64706.2 (57871.6-74781.5) | 2514.5 (2330-2742.6) | -0.01295 (-0.01756 to -0.00834) |
| Republic of Korea | 1941349.8 (1840846.6-2049724.6) | 4572.6 (4344.9-4816.4) | 1726493.9 (1644844.1-1814344.1) | 4572.3 (4336.8-4815.1) | 0.01969 (0.00462 to 0.03476) |
| Republic of Moldova | 143460.1 (133177.9-155949.4) | 3246.6 (3015.1-3519) | 102500.6 (92572.4-114172.4) | 3224.7 (2995.3-3494) | -0.0212 (-0.02216 to -0.02024) |
| Romania | 601976 (540361.4-675672.8) | 2597.3 (2351.2-2889.2) | 494257.3 (436998-568985.3) | 2605.4 (2366.6-2909.7) | 0.01998 (0.01508 to 0.02488) |
| Russian Federation | 6378814.4 (6065957.6-6756589.8) | 4542.4 (4336.4-4786.6) | 5704069.5 (5420703.1-6055483.6) | 4608.2 (4413.7-4842.8) | 0.08942 (0.05682 to 0.12203) |
| Rwanda | 131397.4 (121665-142915.7) | 1939.5 (1761.7-2152.6) | 244450.6 (224153.5-269265.2) | 1940.1 (1762.2-2153.9) | -0.01086 (-0.02354 to 0.00181) |
| Saint Kitts and Nevis | 1381.3 (1290.4-1492.8) | 3242.8 (3016.7-3517.2) | 1799.3 (1646.1-1991.8) | 3239.1 (3013.9-3512.9) | -0.0036 (-0.00406 to -0.00315) |
| Saint Lucia | 4567.3 (4274.2-4924.5) | 3245.5 (3019.4-3519.5) | 5491.2 (5032.9-6039.1) | 3235.5 (3009.6-3508.8) | -0.01134 (-0.01177 to -0.01091) |
| Saint Vincent and the Grenadines | 3655.6 (3421.7-3948.7) | 3240.6 (3014.7-3515.1) | 3618.9 (3347.2-3955.1) | 3233 (3007.2-3504.9) | -0.00765 (-0.00823 to -0.00707) |
| Samoa | 5462.9 (5091.1-5899.6) | 3102.1 (2868-3388.4) | 6828.5 (6344.7-7407.8) | 3107.7 (2872.8-3395.6) | 0.00617 (0.00603 to 0.00632) |
| San Marino | 824.9 (790.3-864.7) | 4082.8 (3899.3-4283) | 1066.9 (1022.5-1119.1) | 4081 (3898.4-4280.1) | -0.00258 (-0.00396 to -0.0012) |
| Sao Tome and Principe | 2860.2 (2673.5-3075.4) | 2342.9 (2164.8-2565.5) | 4985.4 (4635.8-5424) | 2339.4 (2161.2-2561.2) | -0.00525 (-0.00566 to -0.00483) |
| Saudi Arabia | 417201.4 (390342.1-450919.9) | 2536.2 (2349.3-2767.2) | 871929.4 (790760.6-982991.4) | 2535.6 (2348.2-2767) | -0.00128 (-0.00268 to 0.00012) |
| Senegal | 178634.4 (167499.2-191706.8) | 2342.9 (2164.7-2565.3) | 367049.5 (342249.2-397651.7) | 2339.2 (2161.7-2561.8) | -0.00615 (-0.00645 to -0.00586) |
| Serbia | 293907.5 (267527.4-326748.7) | 3197.7 (2950.5-3510.9) | 262188.6 (235350.9-295956) | 3172.9 (2927.2-3482.8) | -0.02551 (-0.02971 to -0.02131) |
| Seychelles | 2355 (2162.8-2598.5) | 3213.7 (2944.1-3561.3) | 3314.9 (3003.6-3741.3) | 3192.7 (2923.9-3537) | -0.02918 (-0.03213 to -0.02623) |
| Sierra Leone | 96118.4 (89910.4-103727.1) | 2340.3 (2162.6-2563) | 204320.9 (190501.9-221589.5) | 2339.5 (2161.6-2561) | -0.00416 (-0.00614 to -0.00218) |
| Singapore | 101016.9 (94232.8-108522.3) | 3798.7 (3541.6-4054.8) | 164425.9 (155720-174574.4) | 3769 (3539.2-4024.7) | -0.0377 (-0.0464 to -0.02899) |
| Slovakia | 164116.2 (150864.1-180778.5) | 3197 (2949.4-3510.8) | 160910.1 (144410.8-181603.3) | 3184.3 (2937.6-3497.3) | -0.01485 (-0.01576 to -0.01394) |
| Slovenia | 59469 (54204.7-65692.5) | 3193.2 (2945.7-3509) | 61954.7 (55501.4-70373.2) | 3174.7 (2928.8-3485.8) | -0.02068 (-0.02235 to -0.019) |
| Solomon Islands | 11104.1 (10362.8-11963.9) | 3103 (2869.6-3390.9) | 21704.7 (20239.9-23601.3) | 3109.6 (2874.2-3398.3) | 0.00822 (0.00781 to 0.00863) |
| Somalia | 171882.3 (161140.1-184985.4) | 2226.4 (2058.6-2445.5) | 469367.6 (439791.1-505422.8) | 2232.2 (2064.5-2450.3) | 0.00637 (0.00534 to 0.0074) |
| South Africa | 866032.3 (803649.1-946293.7) | 2403.4 (2211-2658.6) | 1334997.8 (1222043.3-1485966.3) | 2401.1 (2209.3-2655.2) | -0.00461 (-0.00579 to -0.00343) |
| South Sudan | 128001.7 (119389.9-138042.5) | 2218.5 (2050-2438.5) | 211261.6 (197180.6-227718.5) | 2228.7 (2059.6-2449.1) | 0.01405 (0.01218 to 0.01592) |
| Spain | 1332562.7 (1239107.3-1446523.1) | 3884.8 (3627.2-4224.3) | 1417497.4 (1326289.8-1530163.1) | 3883.7 (3626.1-4223.3) | 0.00056 (-0.00177 to 0.00289) |
| Sri Lanka | 585360.4 (536179.5-646901) | 3411.3 (3131.3-3771.1) | 754836.1 (685833.8-840632.6) | 3401.7 (3116.8-3765.2) | -0.0171 (-0.02292 to -0.01129) |
| Sudan | 537351.2 (504337.5-576952.1) | 2554.6 (2366.2-2788.5) | 1134836.3 (1061046.8-1231157.9) | 2553.1 (2364.3-2786.8) | -0.00092 (-0.00175 to -8e-05) |
| Suriname | 12632.4 (11802.5-13698.9) | 3237.9 (3011.9-3510.5) | 18470.3 (17145.6-20202.9) | 3238.6 (3012.7-3512.6) | -8e-05 (-0.00073 to 0.00057) |
| Sweden | 308290.2 (295415.9-322529.1) | 4139.9 (3962.1-4335.2) | 370394.3 (355096.9-388205.6) | 4136.2 (3958.5-4331.3) | -0.00268 (-0.00323 to -0.00213) |
| Switzerland | 236418.4 (226284-247781.4) | 4074.7 (3892.2-4274.6) | 297100 (284626.9-311527.9) | 4073.1 (3890.5-4272.7) | -0.00181 (-0.00317 to -0.00044) |
| Syrian Arab Republic | 347461.1 (326273.7-372470.7) | 2552.7 (2363.9-2786.2) | 355362.4 (326606.4-392335.9) | 2562.6 (2373.2-2800.7) | 0.01107 (0.00683 to 0.01531) |
| Taiwan (Province of China) | 740815.7 (683579.4-805731.4) | 3695 (3425.7-3993.4) | 837359 (768542.3-927428.7) | 3745.1 (3492.1-4051.4) | 0.11309 (0.07966 to 0.14653) |
| Tajikistan | 371311.1 (350776.7-392591.8) | 5758.3 (5440.5-6126) | 630546.4 (595573.4-669132.7) | 5741.6 (5425.6-6109.2) | -0.0089 (-0.00965 to -0.00814) |
| Thailand | 1809561.2 (1657351.9-2016909.1) | 3216.8 (2946.6-3566) | 2116341.1 (1898947.8-2402004.6) | 3218.2 (2947.6-3567.7) | 0.00185 (0.00085 to 0.00284) |
| Timor-Leste | 25199.6 (23394.4-27638.6) | 3198.8 (2928.5-3546.1) | 45421.5 (41920.5-49801) | 3210.2 (2939.3-3559.8) | 0.0124 (0.01103 to 0.01378) |
| Togo | 83545.3 (77450.5-90772) | 2307.8 (2129.3-2546.3) | 190330 (176423-207812.7) | 2308.2 (2129.2-2547.3) | -0.00541 (-0.00891 to -0.00191) |
| Tokelau | 52 (48.4-56.3) | 3114.5 (2879.5-3402) | 42.9 (39.7-46.7) | 3107.3 (2872.4-3394.8) | -0.01191 (-0.01533 to -0.00848) |
| Tonga | 3221.9 (3003.3-3479.2) | 3114.3 (2878.7-3402.2) | 3410.8 (3171.9-3693.8) | 3116.6 (2880.7-3405.1) | 0.00383 (0.00212 to 0.00553) |
| Trinidad and Tobago | 39635.3 (36980.9-42855.2) | 3238.4 (3012.5-3511.6) | 43950.3 (40398.9-48245.5) | 3235.9 (3010.2-3509.3) | -0.00259 (-0.00281 to -0.00237) |
| Tunisia | 219277.9 (204822-237235.8) | 2554.8 (2366.3-2788.6) | 296222.4 (270887.2-326680.2) | 2557.7 (2368.7-2792) | 0.00362 (0.00334 to 0.0039) |
| Turkey | 2015048.2 (1884637.4-2159688.9) | 3351.5 (3124.5-3622.5) | 2684172.9 (2495775.1-2921017.2) | 3360.8 (3139.2-3624) | -0.00107 (-0.01143 to 0.00928) |
| Turkmenistan | 247688.5 (233747.5-262297.5) | 5768.8 (5449.7-6138.1) | 297007.9 (280430.5-315901.7) | 5721.9 (5406.2-6090.7) | -0.02722 (-0.02822 to -0.02622) |
| Tuvalu | 303 (281.6-330.2) | 3126.2 (2890.4-3413.9) | 384 (355.6-419.9) | 3102.9 (2869.2-3389.2) | -0.02521 (-0.02639 to -0.02402) |
| Uganda | 438912.1 (408919.4-472031.1) | 2531.9 (2341.7-2766.4) | 1106246.8 (1028273.3-1193385.1) | 2534.6 (2344.7-2769.4) | -0.0032 (-0.01197 to 0.00556) |
| Ukraine | 1729346.3 (1584151.6-1916469.2) | 3393 (3141.3-3724.9) | 1341965.4 (1205956.1-1523911.4) | 3378.4 (3127-3708.5) | -0.01141 (-0.01255 to -0.01027) |
| United Arab Emirates | 45191.9 (41686.5-50083) | 2519.7 (2335.7-2750) | 209279 (184372.9-245796.7) | 2509.9 (2325.5-2739.5) | -0.01652 (-0.01887 to -0.01418) |
| United Kingdom | 2316527.2 (2224556-2421238.4) | 4617.6 (4432.8-4831.1) | 2629829.9 (2523590.7-2745400.8) | 4550.2 (4353.2-4746.2) | -0.0707 (-0.10869 to -0.0327) |
| United Republic of Tanzania | 711952.3 (671474-754498.9) | 2683.5 (2494.5-2910.8) | 1591701.2 (1494210-1697989.3) | 2670.5 (2476.7-2893.8) | -0.03794 (-0.05263 to -0.02326) |
| United States Virgin Islands | 3416.5 (3178.1-3718.2) | 3244.4 (3018.2-3517.7) | 2787.8 (2544.5-3126) | 3237.3 (3011-3511.1) | -0.00718 (-0.00907 to -0.00529) |
| United States of America | 13816674.4 (12813082.2-14940685.5) | 5487.6 (5117.1-5917.6) | 15793149.5 (14890625.5-16907489.7) | 4826.4 (4574-5128.9) | -0.19141 (-0.26961 to -0.11315) |
| Uruguay | 113004.4 (107962.1-119204.9) | 3729.4 (3554.5-3932.3) | 111885.3 (106937.5-117706.4) | 3728.1 (3553.5-3931) | -0.00111 (-0.0014 to -0.00082) |
| Uzbekistan | 1410352.4 (1331407-1492506.6) | 5766 (5447-6134.9) | 1978532.8 (1870790.9-2110395.7) | 5749.8 (5432.2-6118) | -0.00821 (-0.00874 to -0.00768) |
| Vanuatu | 4960.3 (4632.4-5361.6) | 3106.6 (2872-3394.9) | 9962.9 (9280.2-10843.4) | 3113 (2877.8-3401.9) | 0.00689 (0.00657 to 0.0072) |
| Venezuela (Bolivarian Republic of) | 714009.2 (668016.3-768821.5) | 3647.1 (3410.3-3938.3) | 948635.6 (880215.4-1031345.7) | 3645.3 (3393-3934.5) | -0.01294 (-0.01741 to -0.00847) |
| Viet Nam | 2238349.7 (2067997.5-2449536) | 3228.3 (2957-3578.6) | 3181192.4 (2891928.5-3568844.2) | 3209.2 (2939.2-3556.3) | -0.01814 (-0.01902 to -0.01727) |
| Yemen | 380380.9 (358444.3-405386.3) | 2558.4 (2369.4-2792.1) | 890978 (833370-963041.1) | 2556.7 (2367.6-2790.9) | -0.00188 (-0.00304 to -0.00073) |
| Zambia | 173055.5 (161867.9-186354) | 2230.7 (2062.2-2450) | 425880.4 (396010-460329.3) | 2232 (2063.3-2451) | 0.00243 (0.00208 to 0.00278) |
| Zimbabwe | 238550.7 (222706-256463.4) | 2284.5 (2107.4-2508.4) | 354587.1 (329532.5-384440.6) | 2289.2 (2110.8-2515.6) | 0.0065 (0.00596 to 0.00705) |

Incidence

| **location** | **1990** | | **2021** | | **EAPC_**  **95%CI** |
| --- | --- | --- | --- | --- | --- |
|  | **Number_95% UI** | **ASR** | **Number_95% UI** | **ASR** |  |

| Afghanistan | 370749.5 (329678-417941) | 4339.9 (3828.3-4996.4) | 1091453.8 (970424.5-1236492.6) | 4349.8 (3834.9-5000.2) | 0.00431 (0.00244 to 0.00619) |
| --- | --- | --- | --- | --- | --- |
| Albania | 139286.6 (120099.7-163713.1) | 4636.2 (4003.5-5440.7) | 146673.2 (124903.3-177312.2) | 4663.6 (4034.4-5480.5) | 0.01626 (0.00811 to 0.02441) |
| Algeria | 928082.1 (825702.5-1053478.6) | 4336.7 (3823.9-4988.1) | 1880981.2 (1662780.3-2166359.4) | 4335.3 (3822.5-4985.4) | -0.00259 (-0.00299 to -0.0022) |
| American Samoa | 2067.7 (1817.2-2379.1) | 4986.3 (4384.6-5813.7) | 2500.7 (2200.9-2905.6) | 4987.7 (4385.6-5817.1) | 0.00215 (0.00108 to 0.00322) |
| Andorra | 1908.5 (1726.1-2121.1) | 3316 (3038.6-3634.5) | 2987.7 (2718.1-3326.1) | 3331.7 (3051.4-3650.1) | 0.018 (0.01445 to 0.02156) |
| Angola | 412269.2 (369402.1-459005.5) | 4880.9 (4357.2-5523.7) | 1317078.8 (1180991.5-1464587.2) | 4896.7 (4368.4-5540.1) | 0.01041 (0.00984 to 0.01098) |
| Antigua and Barbuda | 2786.7 (2446-3225.8) | 4807.6 (4224-5545.5) | 4765.9 (4162.5-5582.1) | 4800.9 (4219-5537.3) | -0.00586 (-0.00758 to -0.00413) |
| Argentina | 926282.7 (856901-999706.3) | 2825.8 (2615.4-3048.4) | 1310483.9 (1209583.7-1421122.6) | 2825 (2614.5-3047.6) | -0.00121 (-0.0015 to -0.00093) |
| Armenia | 159874.7 (138098.5-186818) | 4852.7 (4209.6-5684.9) | 163128.9 (139151.3-194694.7) | 4849.6 (4207.6-5680.7) | -0.00145 (-0.00571 to 0.00281) |
| Australia | 476170.1 (439633.2-517016.1) | 2754.1 (2543-2976.1) | 729994.8 (671270.8-797851.3) | 2757.3 (2543.6-2976.5) | 0.00394 (0.00236 to 0.00552) |
| Austria | 266763.6 (243116.7-293510.2) | 3341.6 (3059.4-3663.9) | 305895 (278382.1-339521.7) | 3336.9 (3055.6-3657.7) | -0.00252 (-0.00412 to -0.00092) |
| Azerbaijan | 329361.3 (284764.2-383921.6) | 4858.1 (4215.4-5691.3) | 532634.1 (456668.2-630853.2) | 4825 (4187.5-5650.6) | -0.02318 (-0.02501 to -0.02135) |
| Bahamas | 11473 (10086.7-13310.2) | 4807 (4223.9-5545.6) | 20092.2 (17556.8-23397.1) | 4805.8 (4223-5544) | -0.00129 (-0.00147 to -0.00112) |
| Bahrain | 19689.2 (17058.2-23138.5) | 4304.6 (3795.2-4936.5) | 68380.5 (59406.4-79748.9) | 4299.7 (3794.9-4925.7) | -0.00411 (-0.00768 to -0.00054) |
| Bangladesh | 4243686.8 (3806322.4-4803703.3) | 4652.5 (4134.3-5284.7) | 7677313.4 (6813114.7-8718410.8) | 4674.6 (4155.4-5315.9) | 0.0182 (0.0164 to 0.01999) |
| Barbados | 12566.6 (10952.8-14554.9) | 4809.3 (4225.8-5549.5) | 16864.3 (14586.4-19923) | 4803.4 (4221.4-5540.7) | -0.00381 (-0.004 to -0.00363) |
| Belarus | 492867.9 (424604-579783.2) | 4398.2 (3816.7-5156.2) | 479653.5 (410465.1-574660.8) | 4379.2 (3800-5131.3) | -0.01017 (-0.01152 to -0.00883) |
| Belgium | 340863.3 (310692.9-375942) | 3339.2 (3057.4-3660.7) | 386019.1 (352142.7-427678.3) | 3341.5 (3059.5-3662) | 0.0028 (0.00237 to 0.00324) |
| Belize | 7291.2 (6454.9-8325.1) | 4790.3 (4211.3-5522.2) | 19890.6 (17563.6-22982.1) | 4794.7 (4214.1-5529.9) | 0.00325 (0.00291 to 0.00359) |
| Benin | 196883.7 (177866.3-219203.9) | 5078.4 (4558.2-5669.3) | 576495.3 (518425.3-642350.3) | 5074.6 (4555.2-5664.3) | -0.00247 (-0.00286 to -0.00208) |
| Bermuda | 3094.8 (2689.4-3607.4) | 4805.7 (4222.9-5543.7) | 3750.3 (3220-4479.1) | 4800.8 (4219.3-5537.4) | -0.00334 (-0.00384 to -0.00284) |
| Bhutan | 24823.7 (22108.9-28215.7) | 4655.6 (4138.3-5285.7) | 35818.7 (31613.9-40692) | 4657.6 (4139.7-5289.6) | 0.00058 (-0.00103 to 0.0022) |
| Bolivia (Plurinational State of) | 269419.5 (236439.7-310457.4) | 5078.1 (4433.4-5873.7) | 582911 (506993.5-674038.9) | 5073.9 (4431.2-5868.8) | -0.00307 (-0.00335 to -0.00278) |
| Bosnia and Herzegovina | 215227.6 (184617.7-254128.7) | 4671.1 (4037.6-5492.7) | 189048.2 (160349.1-231640.8) | 4667.5 (4032.8-5482.8) | -0.01059 (-0.01577 to -0.00541) |
| Botswana | 54069.5 (48451.8-60438.8) | 4904 (4373.8-5546.5) | 115724.4 (102679.7-130657.4) | 4898 (4370.4-5538.9) | -0.0032 (-0.0044 to -0.002) |
| Brazil | 7018892.3 (6191810.3-8117337.9) | 5299.3 (4617.7-6184.2) | 12669802.3 (10947031.3-14934302.9) | 5302.4 (4620.1-6189.4) | 0.00126 (0.00108 to 0.00144) |
| Brunei Darussalam | 7355.9 (6799.4-7964.8) | 2832.7 (2624.2-3051.2) | 13367.6 (12296.2-14482.7) | 2836.1 (2626-3055.3) | 0.00033 (-0.00728 to 0.00794) |
| Bulgaria | 461463.2 (394346-553828.7) | 4678.4 (4041.7-5495.3) | 397316.1 (334465.1-488909.4) | 4657.4 (4024.5-5471.8) | -0.01805 (-0.02036 to -0.01573) |
| Burkina Faso | 390077.6 (352231.2-433156.9) | 5080.6 (4561.1-5671.5) | 967200.8 (871303.4-1076595.3) | 5077.6 (4558.1-5667.4) | -0.00242 (-0.00267 to -0.00217) |
| Burundi | 220005 (197703.9-245237.4) | 4891.4 (4360.2-5532.3) | 541909.3 (486152.7-604837) | 4874.5 (4347.8-5516.4) | -0.01324 (-0.01405 to -0.01243) |
| Cabo Verde | 15211.4 (13666.8-16950.9) | 5092.9 (4570.2-5693.5) | 28997.6 (25990-32620.4) | 5068.7 (4548.4-5655.4) | -0.01658 (-0.01718 to -0.01597) |
| Cambodia | 446041.3 (389411.7-515900.1) | 5452.5 (4736-6353.1) | 900240.2 (778103.4-1055354) | 5439 (4725.3-6337.2) | -0.00794 (-0.00804 to -0.00785) |
| Cameroon | 441629.9 (398515.8-490337.1) | 5126.6 (4599.3-5722.1) | 1415822.3 (1274421.8-1577511.3) | 5123.4 (4596-5715.4) | -0.00223 (-0.00246 to -0.002) |
| Canada | 1385541.5 (1223657.9-1586831.6) | 4771.9 (4238.3-5427.2) | 2000398.4 (1757134-2305136.3) | 4770.9 (4238.1-5426) | 0.00017 (-0.00074 to 0.00108) |
| Central African Republic | 110941.4 (99319-123596.2) | 4892.9 (4365.2-5536.2) | 231786.7 (207464.6-258549.2) | 4895.3 (4367.5-5540) | 0.00089 (-1e-05 to 0.0018) |
| Chad | 247247.9 (223104-274969.2) | 5077.7 (4558.6-5668.1) | 709886.1 (639396.2-791352.1) | 5062.7 (4548.4-5652.5) | -0.01093 (-0.01147 to -0.01038) |
| Chile | 375452.9 (346945.2-406128.7) | 2827.8 (2617.1-3050.8) | 544256.5 (500366.1-594168) | 2820.8 (2610.7-3042.6) | -0.00521 (-0.00617 to -0.00424) |
| China | 61742927.1 (52918193-72709474.8) | 5432.6 (4686.5-6397.1) | 91306112.2 (78062716.6-108832839.2) | 5431.5 (4685.1-6397.3) | 0.00131 (0.00046 to 0.00215) |
| Colombia | 1385402.6 (1221003.5-1602203.8) | 4794.2 (4216.9-5528.8) | 2512343.4 (2198348.5-2936009.4) | 4798.3 (4220.8-5536.4) | 0.00286 (0.00226 to 0.00345) |
| Comoros | 18545.8 (16647.2-20706.8) | 4884.2 (4354-5526.8) | 34800.7 (30974.6-39079.8) | 4884.1 (4355.6-5525.6) | -0.00049 (-0.00086 to -0.00012) |
| Congo | 98038.5 (87845.4-109682.8) | 4872.1 (4343.4-5513.4) | 243007.2 (218383.3-272689.1) | 4863.1 (4336.1-5506.4) | -0.00739 (-0.00787 to -0.00691) |
| Cook Islands | 858.5 (755.1-987.9) | 4980.8 (4380.3-5807.8) | 976.7 (848.7-1153.8) | 5001.6 (4395.3-5836.4) | 0.01564 (0.01437 to 0.0169) |
| Costa Rica | 129102.1 (113798.3-149231.8) | 4792.1 (4215.5-5526.8) | 245639.7 (214378.4-286742.5) | 4800.2 (4221.9-5538.3) | 0.00591 (0.00545 to 0.00636) |
| Croatia | 251910.3 (215155.9-298999.4) | 4676.2 (4039.2-5497) | 244733.8 (206421.4-299941.3) | 4663.6 (4029.5-5477.5) | -0.00954 (-0.01043 to -0.00866) |
| Cuba | 532669.6 (467030.9-613498.4) | 4794.4 (4214.3-5528.9) | 637577.5 (554165-755428.9) | 4795.9 (4215.7-5530.7) | 0.00065 (0.00053 to 0.00077) |
| Cyprus | 26179.4 (23905.6-28796.2) | 3318.9 (3041.3-3632.9) | 47241.5 (42844.3-52427) | 3326.2 (3049.2-3639.8) | 0.00907 (0.00794 to 0.0102) |
| Czechia | 531067 (455823.2-629139.6) | 4677 (4040.4-5496) | 607951.2 (512915.9-745894.8) | 4654.8 (4022.1-5466.5) | -0.01723 (-0.0179 to -0.01656) |
| Côte d'Ivoire | 507869.7 (456296.8-567681.4) | 5053.6 (4539.9-5641) | 1235513.8 (1116036-1379455.3) | 5054.6 (4539.1-5641.1) | -7e-04 (-0.00152 to 0.00012) |
| Democratic People's Republic of Korea | 1012164.7 (887114.7-1174458) | 5017.4 (4405-5856.9) | 1487252.8 (1295587.8-1741635.3) | 4996.8 (4388.5-5825.8) | -0.01457 (-0.01513 to -0.01401) |
| Democratic Republic of the Congo | 1521445.2 (1363964.6-1697882.8) | 4890.3 (4364.1-5533.4) | 3781771.3 (3383993.7-4214842) | 4887.9 (4362.6-5531.3) | -0.00257 (-0.00335 to -0.00179) |
| Denmark | 139531.5 (130109-149845.7) | 2725.9 (2545-2925.4) | 153150.5 (142734.3-164010.1) | 2725.8 (2544.9-2925) | 0.00531 (0.0029 to 0.00772) |
| Djibouti | 17169.6 (15378.3-19223.3) | 4870.4 (4344.6-5513) | 58371.6 (52126.5-65863) | 4862.5 (4337.7-5507.3) | -0.00544 (-0.00587 to -0.00501) |
| Dominica | 3199.6 (2810.6-3673.8) | 4808.7 (4226.2-5550.4) | 3510.8 (3076.1-4095.9) | 4792.4 (4212.7-5523.9) | -0.01267 (-0.01362 to -0.01172) |
| Dominican Republic | 297275.9 (261806-341377.1) | 4798.7 (4217.6-5535.3) | 531649.3 (466425.6-611048.7) | 4795 (4215.1-5528.8) | -0.00452 (-0.00532 to -0.00373) |
| Ecuador | 435439.5 (379991.9-502501) | 5075.1 (4432-5870.7) | 914537.2 (798106.9-1053270.9) | 5076.7 (4434.6-5871.1) | 0.00127 (0.00101 to 0.00153) |
| Egypt | 2068030.5 (1836856.1-2345976.7) | 4325.5 (3805.2-4967.8) | 4198801.1 (3722220.3-4794568.8) | 4318.6 (3798.7-4960.2) | -0.00673 (-0.00859 to -0.00486) |
| El Salvador | 215756.4 (191038.7-246785.2) | 4800.9 (4222.7-5538.4) | 308109.8 (270969.1-353730.7) | 4813.5 (4232.3-5558.3) | 0.00858 (0.00805 to 0.0091) |
| Equatorial Guinea | 16925.6 (15154.3-18834) | 4901 (4370.3-5545.8) | 65290.6 (58321.1-73203.6) | 4883.6 (4358.9-5530.5) | -0.01461 (-0.01667 to -0.01256) |
| Eritrea | 135275.4 (121346.3-150967.9) | 4895.5 (4362.9-5539.4) | 288053 (257784.5-323479.9) | 4890 (4360.6-5534.1) | -0.00386 (-0.00459 to -0.00312) |
| Estonia | 75646.7 (65280.2-89228.6) | 4441.2 (3857.5-5194) | 68712.3 (59008.7-81922.5) | 4402.5 (3824-5148.1) | -0.03341 (-0.03655 to -0.03027) |
| Eswatini | 31916.8 (28689.8-35635.6) | 4907.7 (4375.8-5553.2) | 52075.2 (46438.7-58382.5) | 4906 (4376.2-5550.2) | -0.00103 (-0.00165 to -0.00041) |
| Ethiopia | 2079896 (1871437.7-2331330) | 5255 (4682.9-5955) | 4843625.3 (4325566-5457288.8) | 5253.8 (4680.8-5954.7) | 0.00228 (7e-04 to 0.00387) |
| Fiji | 33331.1 (29302.6-38473.4) | 4991.4 (4388.9-5822.2) | 45611 (40078.8-53176.8) | 4994.4 (4391.7-5823.8) | 0.00088 (0.00056 to 0.0012) |
| Finland | 172450.2 (156827.5-190336.4) | 3340.4 (3058.3-3662.6) | 184827.2 (168120.8-205109.4) | 3335.6 (3054.7-3656) | -0.00448 (-0.0048 to -0.00415) |
| France | 2643279.7 (2342963.5-3031530.7) | 4416.9 (3937.4-5043.9) | 3037443.3 (2692449.2-3476100.4) | 4420.7 (3941.2-5048.3) | 0.00099 (-0.00034 to 0.00231) |
| Gabon | 42077.3 (37690.3-46967.3) | 4923.8 (4392.3-5568.1) | 83218.3 (74388.7-93260.5) | 4927 (4395.4-5570.7) | 0.00123 (0.00079 to 0.00168) |
| Gambia | 40854.2 (36840.7-45633) | 5059.4 (4545.1-5647.5) | 106096.3 (95658.5-118415.6) | 5071 (4553.3-5660.3) | 0.00879 (0.00762 to 0.00997) |
| Georgia | 281191.9 (242505.8-329776.4) | 4860.4 (4209.8-5698.4) | 198981.2 (170575.9-239462.6) | 4827.6 (4186.3-5655.5) | -0.02121 (-0.02455 to -0.01788) |
| Germany | 3286401.4 (2931716.7-3674053.7) | 3912.5 (3525.1-4340.2) | 3436421 (3073369.8-3812710.3) | 3903.9 (3525.8-4344.5) | -0.00445 (-0.00639 to -0.00251) |
| Ghana | 645860.4 (583452.7-719450.6) | 5106.8 (4576.7-5719.2) | 1599544.1 (1434149.4-1781010.2) | 5117 (4584.9-5729) | 0.00938 (0.00627 to 0.01248) |
| Greece | 351411.9 (320845-387471.2) | 3318.1 (3045.3-3637.2) | 342543.8 (310758-380157) | 3321.1 (3045.7-3643.3) | 0.00622 (0.00317 to 0.00926) |
| Greenland | 2630.3 (2303.1-3035.9) | 4712.4 (4190.1-5362.7) | 2811.3 (2474.1-3220.1) | 4742.4 (4215-5395.3) | 0.01795 (0.01689 to 0.01901) |
| Grenada | 3702.8 (3274.1-4227.5) | 4806.3 (4223.6-5544.9) | 5290.4 (4627.2-6159.4) | 4793.6 (4214.1-5523.9) | -0.00778 (-0.00898 to -0.00657) |
| Guam | 6416.3 (5601.8-7444.4) | 4977.1 (4377-5800.5) | 8551.8 (7438.5-10048.9) | 4990.2 (4388.2-5819.6) | 0.00734 (0.00529 to 0.0094) |
| Guatemala | 313507.6 (278777-357293.4) | 4793.7 (4216.3-5527.4) | 711712.8 (627186.6-821553.5) | 4801.5 (4222.7-5540.2) | 0.00508 (0.00478 to 0.00538) |
| Guinea | 254369.4 (230411.5-283340.3) | 5073.7 (4555.8-5664.3) | 576341.9 (519234.5-641847.6) | 5074.1 (4556.7-5665.1) | 0.00022 (-0.00028 to 0.00072) |
| Guinea-Bissau | 41777.6 (37653.4-46557) | 5077.2 (4558.6-5667.9) | 89818.3 (80888.6-100437.3) | 5079.5 (4559.4-5670) | 0.0013 (0.00115 to 0.00145) |
| Guyana | 32326.8 (28457.8-37144.4) | 4798.4 (4217.4-5533.4) | 36396.9 (31973.5-42010.5) | 4801.9 (4219.9-5538.5) | 0.0021 (0.00135 to 0.00285) |
| Haiti | 256005.1 (227557.8-293612.5) | 4802 (4219.8-5538.6) | 567044.9 (499850.3-653869.9) | 4802.4 (4220.6-5540.4) | -0.00019 (-0.00037 to -1e-05) |
| Honduras | 178557.9 (158564-203654.1) | 4794.4 (4217.3-5528.9) | 450029.1 (397167.7-519869.8) | 4801.6 (4222.6-5540) | 0.00462 (0.00387 to 0.00538) |
| Hungary | 553518.1 (474045.2-658970.4) | 4752.1 (4112.3-5570.4) | 560154.2 (473095.1-685736.7) | 4734.4 (4097.5-5548.8) | -0.01609 (-0.01884 to -0.01334) |
| Iceland | 8633 (7878.1-9467.5) | 3367.6 (3085.8-3685.4) | 11907 (10866.7-13148.9) | 3361.2 (3087.5-3678.1) | -0.00696 (-0.00943 to -0.00449) |
| India | 36435550.8 (32310651.2-41446675.2) | 4858.3 (4281.5-5553.7) | 69127370.9 (60731944.4-79233471.1) | 4863.2 (4285.1-5559.6) | 0.00284 (0.00173 to 0.00396) |
| Indonesia | 9607877.1 (8284407.6-11315065.8) | 5969 (5129.8-7044.4) | 17465367.6 (14974711.3-20734695.5) | 5965.7 (5127.9-7039.2) | -0.00165 (-0.00193 to -0.00137) |
| Iran (Islamic Republic of) | 2195284.3 (1939020.5-2490164.4) | 4745.9 (4115.7-5485.7) | 4246905.3 (3656579.1-4958751.9) | 4753.2 (4120.6-5504.3) | 0.00809 (0.00711 to 0.00907) |
| Iraq | 655233.4 (583825.9-741550.6) | 4338.1 (3825.3-4985.9) | 1664517.9 (1477739.7-1905598.1) | 4336.1 (3823-4982.5) | -0.00238 (-0.00285 to -0.00192) |
| Ireland | 120271.7 (110079.4-131583.1) | 3341.7 (3059.6-3662.6) | 167895.3 (153324-184884.4) | 3347.1 (3064.9-3666.9) | 0.0058 (0.00374 to 0.00785) |
| Israel | 164252 (150370.1-179436.4) | 3349.3 (3067.3-3670) | 318106.3 (291602-347664.9) | 3342.1 (3060.5-3662.8) | -0.00754 (-0.0077 to -0.00737) |
| Italy | 2296388.9 (2077008.6-2566457.6) | 3894.2 (3543.4-4345.6) | 2390017.8 (2146011.3-2714526) | 3887.4 (3537.5-4337.9) | -0.00579 (-0.00649 to -0.00509) |
| Jamaica | 102609 (90372.4-117752.7) | 4801.8 (4219.9-5538) | 143705.5 (125611.6-167103.9) | 4797.4 (4216.3-5532.4) | -0.00291 (-0.00318 to -0.00264) |
| Japan | 3953795.3 (3646825.3-4313825.4) | 3057.2 (2842.3-3307.1) | 4019436.2 (3662190.9-4439323.1) | 3054 (2838.5-3301.6) | -0.00711 (-0.01126 to -0.00297) |
| Jordan | 132215.5 (116938.8-150232.2) | 4332.3 (3816.4-4974.9) | 510482.4 (451048.4-583861.9) | 4323.2 (3808.6-4963.1) | -0.00439 (-0.0058 to -0.00299) |
| Kazakhstan | 756501 (655862.8-884308.9) | 4853.9 (4208.3-5687.7) | 926573.3 (801018.4-1091464.5) | 4849.6 (4206.4-5679.8) | -0.00137 (-0.00383 to 0.0011) |
| Kenya | 938842.4 (843483.4-1049735) | 5249.2 (4675.2-5951.5) | 2333596.1 (2084532.3-2630742) | 5260.3 (4687.6-5963.1) | 0.00771 (0.00679 to 0.00862) |
| Kiribati | 3174.8 (2787.9-3651.9) | 5005.3 (4399.9-5840) | 5557.2 (4889.9-6398.6) | 5010.5 (4404.1-5849.3) | 0.00321 (0.00282 to 0.00361) |
| Kuwait | 67142.2 (58446.5-78552.7) | 4295.3 (3788.9-4927.4) | 213730 (184112.5-250283.5) | 4325.3 (3818.8-4966.6) | 0.02233 (0.0199 to 0.02477) |
| Kyrgyzstan | 193957.9 (168759-224201.9) | 4847.4 (4203.2-5678.9) | 311959.4 (271320.7-364866.4) | 4843.5 (4201.6-5671.8) | 0.00083 (-0.00076 to 0.00243) |
| Lao People's Democratic Republic | 184243.4 (160855.4-213071.6) | 5433.9 (4719.7-6330.7) | 380671.4 (329054.9-446637.3) | 5424.3 (4711.3-6317.5) | -0.00576 (-0.00628 to -0.00525) |
| Latvia | 126427.9 (108923.5-149824) | 4322.9 (3737.6-5074.6) | 97761.7 (83657.3-117597.1) | 4294.7 (3709.3-5039.9) | -0.02191 (-0.0243 to -0.01952) |
| Lebanon | 120268.5 (106711.1-136986.6) | 4349.7 (3837.3-5001.2) | 252844.1 (222088.9-292159.4) | 4354.7 (3842.8-5004.9) | 0.00529 (0.00417 to 0.00641) |
| Lesotho | 64046.6 (57301.5-71124.2) | 4926.4 (4389.1-5574.9) | 86154.3 (76712.2-96672.3) | 4908.2 (4378.1-5554) | -0.01195 (-0.01225 to -0.01165) |
| Liberia | 103761.8 (93628.9-115521.4) | 5058.6 (4544.9-5647.1) | 246726.4 (222799.9-275081.3) | 5059.3 (4543.6-5646.6) | -0.00509 (-0.00639 to -0.00379) |
| Libya | 152319.4 (135586.7-172465.1) | 4312 (3800-4953.2) | 308000.2 (270062.8-355725.2) | 4333.1 (3820.6-4980.9) | 0.01859 (0.0154 to 0.02178) |
| Lithuania | 173239.9 (149667.3-203048) | 4399.6 (3818.1-5154.7) | 144999.7 (124294.4-173824.1) | 4375.4 (3797-5126.7) | -0.01858 (-0.02034 to -0.01682) |
| Luxembourg | 13177.7 (11984.7-14550.1) | 3338.7 (3057-3660.4) | 22269.9 (20298.4-24654.2) | 3335.8 (3054.7-3656.2) | -0.00316 (-0.00423 to -0.00209) |
| Madagascar | 477130.1 (428526.4-532162.5) | 4879.5 (4350.9-5520.5) | 1211776.8 (1083700.2-1352888.8) | 4885 (4355-5527.2) | 0.00344 (0.00317 to 0.00371) |
| Malawi | 388664.4 (348849.9-433148.9) | 4886.5 (4357-5527.8) | 817385.3 (732311-912482) | 4891.8 (4360.8-5534.3) | 0.00469 (0.00432 to 0.00506) |
| Malaysia | 844916.4 (732655.3-984807.5) | 5423.2 (4710.3-6316.5) | 1791770.3 (1543850.2-2093720.1) | 5414.2 (4703.3-6303.7) | -0.00608 (-0.00638 to -0.00578) |
| Maldives | 9326.3 (8155.8-10741.6) | 5400.4 (4689.4-6290) | 30309.3 (25796.3-36178.3) | 5368.5 (4665.6-6250.5) | -0.02 (-0.02689 to -0.01311) |
| Mali | 360749.1 (325263-401369) | 5080.6 (4561.3-5672.2) | 996819.3 (897185-1111726.2) | 5075.5 (4556.8-5667.1) | -0.00351 (-0.00429 to -0.00272) |
| Malta | 12689.5 (11602.7-13956.3) | 3343.7 (3062.2-3664.6) | 14902.8 (13521.3-16603.2) | 3333 (3052.4-3652.9) | -0.01056 (-0.01092 to -0.01019) |
| Marshall Islands | 1743.3 (1542.8-1995.8) | 4988.9 (4387-5815.7) | 2673.3 (2344.3-3090.6) | 4986.4 (4384-5815.6) | -0.00252 (-0.00331 to -0.00173) |
| Mauritania | 87698.3 (78924.7-97550.8) | 5071.8 (4553-5660.8) | 194584.7 (175364-216485.4) | 5070.5 (4552.5-5660.1) | -0.00066 (-0.00117 to -0.00015) |
| Mauritius | 57119.8 (49240.8-67224.7) | 5429.9 (4717.7-6323.6) | 79671.6 (68437-93862.7) | 5427.2 (4714.5-6320.5) | -0.00183 (-0.00234 to -0.00131) |
| Mexico | 3711833.2 (3255697.3-4274316.4) | 5227.4 (4546.5-6112.1) | 7010841.2 (6073745.4-8210730.7) | 5229.4 (4546.7-6116.3) | -0.00013 (-0.00083 to 0.00057) |
| Micronesia (Federated States of) | 4183.5 (3695.5-4794.4) | 4989.9 (4388.3-5816.5) | 4936.8 (4344.4-5708.8) | 4993.4 (4391.4-5822) | 0.00148 (0.00018 to 0.00278) |
| Monaco | 1038.5 (941.4-1153.1) | 3346.1 (3064.2-3667.6) | 1259.5 (1142-1408.3) | 3348.6 (3066.5-3668.9) | 0.00741 (0.00429 to 0.01052) |
| Mongolia | 87917.2 (76616-101606.7) | 4825.8 (4186.5-5648.3) | 153581.8 (133243.8-179722.1) | 4846.5 (4204.2-5676.4) | 0.01325 (0.01223 to 0.01426) |
| Montenegro | 29931.7 (25694.8-35157) | 4672.7 (4039.7-5492.8) | 33698.7 (28643.8-40878.6) | 4666.7 (4033.8-5483) | -0.00202 (-0.00416 to 0.00012) |
| Morocco | 964234.9 (858009.3-1094287.9) | 4333.2 (3822.2-4985.1) | 1634380.7 (1441871.1-1885884.7) | 4338.4 (3827.3-4988.3) | 0.00452 (0.00368 to 0.00537) |
| Mozambique | 538191.5 (482355.7-599141.6) | 4892.3 (4359.8-5535.6) | 1251480.1 (1123518.9-1396643.6) | 4896.1 (4362.8-5538.7) | 0.00209 (0.00184 to 0.00233) |
| Myanmar | 1945015.1 (1689412.2-2267446.5) | 5431.9 (4717.6-6327.9) | 3079322.9 (2678228.3-3587835.1) | 5439.8 (4725.6-6338.4) | 0.00466 (0.00419 to 0.00513) |
| Namibia | 58411.7 (52219.1-65270.5) | 4899 (4370.7-5541.5) | 111946.7 (99628-125759.8) | 4903.7 (4374.5-5546.5) | 0.00267 (0.00243 to 0.00292) |
| Nauru | 427.9 (377.1-492.5) | 4972.3 (4371.9-5799) | 491.4 (432.1-564.8) | 5001.5 (4395.9-5837.2) | 0.01871 (0.01723 to 0.02019) |
| Nepal | 809083 (724649.6-912527.9) | 4845.9 (4313.6-5495.9) | 1471919.2 (1309130.4-1668018.4) | 4868.2 (4331.8-5523.2) | 0.01658 (0.01455 to 0.0186) |
| Netherlands | 521738.4 (473748.3-579952.4) | 3374.8 (3079.3-3703.2) | 557856.5 (509395.1-614736.4) | 3237 (2970.5-3539.8) | -0.09163 (-0.13269 to -0.05055) |
| New Zealand | 103556.9 (95574.6-112598.7) | 2977.5 (2758.6-3225) | 157874.3 (145230.9-172790.8) | 2975.7 (2755.4-3221) | 0.00299 (-0.00071 to 0.00669) |
| Nicaragua | 146303.5 (129766.8-166733.5) | 4799.3 (4220.9-5534.4) | 309310.2 (271558.2-356284.5) | 4799.5 (4221.6-5537.9) | 5e-04 (0.00026 to 0.00074) |
| Niger | 322084.1 (289640-358518.4) | 5064.5 (4549-5653.9) | 997784.5 (899763.3-1114649.3) | 5070.8 (4551.9-5660.4) | 0.00478 (0.00431 to 0.00526) |
| Nigeria | 4176139.2 (3754808.2-4685890.9) | 5538.7 (4946.9-6275.8) | 10701223 (9615190.6-11971526.5) | 5555.3 (4967-6284.6) | 0.01089 (0.01017 to 0.01161) |
| Niue | 107.9 (95-124.3) | 4997.7 (4394.2-5828.3) | 89.8 (78.4-105.4) | 4995.5 (4391.9-5826.6) | -0.00142 (-0.00269 to -0.00016) |
| North Macedonia | 94208.5 (81145.3-110897.8) | 4671.2 (4036.5-5486.1) | 120371.6 (102162-146032.5) | 4646.3 (4015.8-5456.1) | -0.02167 (-0.02313 to -0.02021) |
| Northern Mariana Islands | 2170.2 (1862.5-2566.4) | 4963.4 (4362.7-5779.3) | 2603.3 (2274.5-3046.3) | 4979.4 (4379.2-5803.6) | 0.00758 (0.00313 to 0.01202) |
| Norway | 157816.5 (140635.7-178494.8) | 3573 (3209.3-4008.9) | 203034.9 (180713.2-230909.7) | 3571.1 (3210.8-4004.2) | -7e-04 (-0.00376 to 0.00237) |
| Oman | 71493.9 (63200.9-81996) | 4301.3 (3798.3-4925.2) | 196418.2 (169659.6-230970.9) | 4293 (3787.8-4919.9) | 0.00051 (-0.00256 to 0.00358) |
| Pakistan | 4623182.3 (4123479.6-5196832.1) | 5036.2 (4434.2-5756.3) | 10667375 (9465188.5-12138667) | 5049.3 (4447-5774.1) | 0.00874 (0.00855 to 0.00894) |
| Palau | 719.4 (629-830.9) | 4987.5 (4385.6-5816.6) | 1029.9 (892-1225.9) | 4964.3 (4365.4-5784.9) | -0.01251 (-0.01682 to -0.0082) |
| Palestine | 71276.7 (63416.8-80573.9) | 4358.4 (3846.1-5010.7) | 199345.5 (177175.1-226798.5) | 4343.9 (3829.7-4990) | -0.00956 (-0.0105 to -0.00862) |
| Panama | 103376.9 (91335.4-118884.2) | 4786.6 (4210.8-5518.6) | 209067.1 (183730.5-241920.3) | 4789.7 (4213.6-5523.7) | 0.00227 (0.00204 to 0.0025) |
| Papua New Guinea | 170646.4 (150465.1-195755.7) | 4982 (4380.8-5810.3) | 460096.1 (405425.9-530187.4) | 4981.3 (4380.4-5811.1) | -0.00162 (-0.00203 to -0.00121) |
| Paraguay | 166318.7 (147964.5-190337.1) | 4833.1 (4257.3-5565.1) | 341608.7 (300201.8-393432.7) | 4832.2 (4256.6-5564.1) | -0.00088 (-0.00103 to -0.00072) |
| Peru | 987035.6 (868486.3-1136176.8) | 5263.4 (4619.9-6037.3) | 1939757.5 (1694113.5-2237574.6) | 5262.6 (4619.2-6036.8) | -0.00016 (-0.00118 to 0.00087) |
| Philippines | 3112739.8 (2697149.8-3650276.2) | 5967.5 (5127.2-7046.7) | 6499848.6 (5595092-7648194.8) | 5968.8 (5129.7-7047.9) | 0.00111 (0.00092 to 0.00131) |
| Poland | 2087731.6 (1775720.4-2471065.9) | 5162.4 (4404.6-6084.9) | 2453937.2 (2058960.2-2998925.1) | 5145.7 (4391.1-6065.6) | -0.01182 (-0.01256 to -0.01108) |
| Portugal | 345029.2 (315254.5-379451.2) | 3351.2 (3068.5-3672.5) | 360733.9 (327843.2-400893.9) | 3350.5 (3068-3671.4) | 0.00102 (-4e-04 to 0.00244) |
| Puerto Rico | 174629.9 (153179.1-200972.8) | 4805.4 (4222.7-5543.5) | 191167.3 (164783.6-226493.2) | 4804.9 (4222.4-5543) | -0.00082 (-0.00115 to -0.00049) |
| Qatar | 17691.4 (15140.1-21028.1) | 4272.6 (3774.6-4894.9) | 131543.7 (111692.5-158788.2) | 4264.8 (3768.1-4886.5) | -0.00751 (-0.0103 to -0.00471) |
| Republic of Korea | 1287526.9 (1183109.1-1397885.1) | 2865 (2648-3088.9) | 1524339 (1396384.4-1674362.4) | 2857.7 (2648.9-3081.4) | -0.00619 (-0.00845 to -0.00392) |
| Republic of Moldova | 198014.9 (170958.4-233092.8) | 4407.5 (3825.1-5168.3) | 185728.3 (158424.9-222199.2) | 4373.6 (3794.7-5125.2) | -0.02415 (-0.02513 to -0.02316) |
| Romania | 1160062.7 (994662-1374211.7) | 4619 (3973.4-5450) | 1071573.3 (905419-1313937.7) | 4596.8 (3957.6-5418.6) | -0.01212 (-0.01421 to -0.01002) |
| Russian Federation | 7042145.1 (6129661.4-8137745) | 4373.7 (3825.3-5003.2) | 7140264.9 (6243454.7-8254080.4) | 4300.5 (3773-4903.9) | -0.02295 (-0.03206 to -0.01384) |
| Rwanda | 281218.2 (252512-314211.6) | 4859.5 (4330.8-5499.1) | 580042.8 (518311.3-650212.7) | 4862.6 (4333.1-5503.8) | 0.00016 (-0.00147 to 0.00179) |
| Saint Kitts and Nevis | 1847.4 (1623-2139.7) | 4805.1 (4222.4-5542.8) | 3184.8 (2774.4-3742.8) | 4798.1 (4216.9-5532.2) | -0.00461 (-0.00547 to -0.00375) |
| Saint Lucia | 5749.1 (5073.3-6595.9) | 4807.3 (4223.8-5545.5) | 9708.2 (8474.1-11352.5) | 4795.6 (4215.2-5529.3) | -0.00832 (-0.00857 to -0.00807) |
| Saint Vincent and the Grenadines | 4605.7 (4061.4-5280.1) | 4802.2 (4220.4-5539.7) | 5925.1 (5196.1-6896.7) | 4789.4 (4209.7-5522.2) | -0.00847 (-0.00916 to -0.00779) |
| Samoa | 6938.4 (6126.1-7913.6) | 4986.6 (4385.3-5815.5) | 9524.1 (8422-10955.2) | 4987.3 (4385.3-5814.8) | 0.00068 (0.00053 to 0.00084) |
| San Marino | 818.4 (747-900.4) | 3344.5 (3062.1-3665.2) | 1111.6 (1010.4-1236.2) | 3353.6 (3071.7-3674.1) | 0.00931 (0.00675 to 0.01187) |
| Sao Tome and Principe | 5113.8 (4615.1-5690.8) | 5074.7 (4556.4-5664.2) | 10221.3 (9225.3-11414.7) | 5066.8 (4548.4-5654.4) | -0.00592 (-0.00639 to -0.00545) |
| Saudi Arabia | 574756.5 (510037.4-654824.6) | 4300.9 (3791.9-4934.3) | 1664125.5 (1436694.2-1943619.4) | 4308.9 (3799.2-4944.5) | 0.01087 (0.00882 to 0.01292) |
| Senegal | 316008.6 (284821.8-351579.2) | 5071 (4554-5660.9) | 713702.5 (643510.9-795140.2) | 5068.9 (4550-5657.3) | -0.00172 (-0.00181 to -0.00163) |
| Serbia | 486201.4 (414887.3-578097.6) | 4670.7 (4036.5-5484.8) | 503840.9 (427330.5-612332.8) | 4649.2 (4019.7-5462.8) | -0.01011 (-0.01609 to -0.00413) |
| Seychelles | 3681.2 (3189.4-4285.1) | 5428 (4718.5-6317.9) | 6212.2 (5372.4-7276.6) | 5409.4 (4702.4-6296.8) | -0.01398 (-0.01543 to -0.01253) |
| Sierra Leone | 178797.5 (161230.6-199063.5) | 5066.9 (4550.3-5656.1) | 396182.9 (357460.1-441847) | 5065.2 (4548.6-5653.9) | -0.0037 (-0.00555 to -0.00186) |
| Singapore | 89377.8 (81981.2-97419.2) | 2821.8 (2602.5-3044.5) | 170419.8 (156433.4-187512) | 2816.6 (2607.2-3034) | -0.01243 (-0.01833 to -0.00653) |
| Slovakia | 259078.8 (222693.4-306309.8) | 4685.4 (4048.1-5505.8) | 305111.9 (258329.6-373024.1) | 4661.3 (4027.3-5475.4) | -0.01921 (-0.02044 to -0.01798) |
| Slovenia | 101078.9 (86599.4-119427.8) | 4678.1 (4040-5499.3) | 119326.4 (100670.9-146514.2) | 4643.9 (4013.2-5452.3) | -0.02619 (-0.02844 to -0.02395) |
| Solomon Islands | 13304.9 (11777.6-15167.4) | 4975.9 (4375.3-5801) | 29881.1 (26382.3-34262.5) | 4989.9 (4387.6-5820.5) | 0.00964 (0.00946 to 0.00982) |
| Somalia | 308695.7 (278620.1-343844.7) | 4879.5 (4351-5521.9) | 842931.4 (761090.2-943087.5) | 4889.8 (4359.7-5533.5) | 0.00514 (0.00417 to 0.00611) |
| South Africa | 1745748.8 (1558759.8-1963114.6) | 5289 (4709.9-5999.1) | 3045987.6 (2697066.2-3453663.4) | 5286.3 (4708.5-5995.2) | -0.00284 (-0.00416 to -0.00151) |
| South Sudan | 237294.1 (212992.8-264879.3) | 4858.5 (4334.1-5502.6) | 396435.8 (356496-440788.7) | 4879.6 (4349.3-5524.7) | 0.01367 (0.0122 to 0.01513) |
| Spain | 1314184.6 (1201380.9-1443408.5) | 3332.8 (3057.2-3657.6) | 1548332.6 (1407772.4-1725930.9) | 3330.8 (3055.5-3655.2) | -0.00356 (-0.00558 to -0.00154) |
| Sri Lanka | 927130.8 (796949-1093618.1) | 5742.5 (4946.1-6703.5) | 1376346.1 (1183138.2-1620542.5) | 5755.8 (4959.6-6720.4) | 0.00752 (0.0065 to 0.00854) |
| Sudan | 718047.2 (640731.3-811253.3) | 4335 (3824.7-4985.3) | 1646714 (1464169.3-1868718.2) | 4334 (3823-4983.3) | 0.00305 (0.00111 to 0.00498) |
| Suriname | 16966.7 (14958.4-19480.9) | 4795.9 (4214.9-5531.6) | 29133 (25543.9-33833.5) | 4801.8 (4220-5538) | 0.00428 (0.00371 to 0.00486) |
| Sweden | 312312.4 (281678.4-345444.1) | 3525.5 (3205.9-3896.4) | 372740.7 (336780.5-414498.2) | 3521.4 (3203-3892.4) | -0.00313 (-0.00385 to -0.00241) |
| Switzerland | 237385.8 (216361.7-262020.8) | 3336.5 (3055.3-3657.8) | 303498.1 (275914.8-337016.1) | 3335.9 (3054.8-3656.3) | -0.00131 (-0.00354 to 0.00092) |
| Syrian Arab Republic | 446248.4 (398007.5-504420.8) | 4330.7 (3820.4-4979.1) | 621719.3 (545776-711977.6) | 4341.3 (3828.3-4996.8) | 0.00325 (0.00113 to 0.00536) |
| Taiwan (Province of China) | 1020251.1 (888819.4-1188909) | 5027.4 (4424.2-5846) | 1447137.7 (1246541.8-1738282.7) | 5042.7 (4436.9-5871.9) | 0.01553 (0.01338 to 0.01769) |
| Tajikistan | 217939.6 (191023.7-251655.9) | 4832.7 (4190.7-5659) | 444635.4 (386881.8-518238.9) | 4825.7 (4188.7-5648.9) | -0.00183 (-0.00323 to -0.00043) |
| Thailand | 2953339.8 (2552738.9-3470352.8) | 5430.6 (4716-6326.5) | 4315354 (3718008.9-5083774.8) | 5432.3 (4717.8-6329.1) | 0.00074 (0.00047 to 0.001) |
| Timor-Leste | 34639 (30046.2-40408.3) | 5414.8 (4701.9-6307.1) | 66268.4 (57692-76700.9) | 5420.9 (4706.8-6315.4) | 0.00383 (0.00344 to 0.00422) |
| Togo | 150137 (135013.3-167606.1) | 5074.2 (4554.4-5667.1) | 380981.2 (343200.6-424814) | 5077.6 (4554.4-5670.1) | 0.00246 (0.0019 to 0.00302) |
| Tokelau | 71.5 (62.9-82.3) | 4999.9 (4393.6-5835.6) | 69 (60.5-80.6) | 4991 (4388.2-5821.1) | -0.00746 (-0.00915 to -0.00578) |
| Tonga | 4103.7 (3624.5-4678.4) | 4997.8 (4393-5832.8) | 4804.9 (4232.4-5507.4) | 5000.6 (4395.3-5834.1) | 0.0022 (0.0012 to 0.0032) |
| Trinidad and Tobago | 53621.3 (47170.8-61843.2) | 4797.4 (4216.5-5531.2) | 74969.6 (65214.5-87940.8) | 4795.2 (4214.9-5528.8) | -0.00159 (-0.00182 to -0.00136) |
| Tunisia | 323697.4 (287834.6-368105.4) | 4334.9 (3824.5-4984.9) | 546225.5 (478823.9-629210.8) | 4346.6 (3834.9-4997.8) | 0.00864 (0.00846 to 0.00882) |
| Turkey | 2301548.9 (2048486.4-2608872.9) | 4405 (3886.3-5051.9) | 3914094.3 (3434112.9-4502906.6) | 4406.3 (3891.2-5050.6) | -0.00089 (-0.0017 to -8e-05) |
| Turkmenistan | 153474.6 (133809.8-177635.9) | 4847.1 (4204.1-5678) | 240022.7 (208355.9-280790) | 4798.1 (4162.4-5611.4) | -0.03393 (-0.0343 to -0.03356) |
| Tuvalu | 432.1 (379.1-501.6) | 5016.4 (4406-5854.1) | 598.1 (524.3-694.7) | 4987.8 (4385.2-5816.9) | -0.01923 (-0.01957 to -0.0189) |
| Uganda | 672691.2 (602987.8-750835.5) | 4912.4 (4383.3-5554.7) | 1753529.7 (1574061.2-1957021.9) | 4921.7 (4389.9-5567.3) | 0.00509 (0.00395 to 0.00624) |
| Ukraine | 2811657.7 (2391390.1-3349055.1) | 4766 (4093.5-5662.8) | 2533830.1 (2145946.6-3073344.3) | 4745.6 (4075.5-5632.4) | -0.00982 (-0.01178 to -0.00785) |
| United Arab Emirates | 72305 (62131.4-85666.6) | 4276.7 (3777.3-4895) | 452623.7 (378315.3-546655.7) | 4241.8 (3746.9-4865.1) | -0.02608 (-0.03301 to -0.01914) |
| United Kingdom | 2079659.8 (1883269.7-2297700.2) | 3544.6 (3237.7-3905.1) | 2456496.4 (2219985.5-2721142.6) | 3543.9 (3228.9-3907.6) | -0.0046 (-0.00985 to 0.00065) |
| United Republic of Tanzania | 1040045.6 (935326.7-1162448.5) | 4927.7 (4394.6-5572) | 2494746.4 (2231351.4-2775910.4) | 4927.4 (4395.8-5567.8) | -0.00164 (-0.00227 to -0.00102) |
| United States Virgin Islands | 4997.2 (4408.3-5755.9) | 4806.7 (4223.3-5545.2) | 4984.2 (4270.3-5978.3) | 4801.2 (4219.2-5536.9) | -0.00313 (-0.00442 to -0.00185) |
| United States of America | 19649783 (17070502.7-22884442.2) | 7245.3 (6330.9-8394.4) | 21370988.2 (18804608.9-24535304.5) | 5763.2 (5105.5-6543.6) | -0.30811 (-0.47174 to -0.14422) |
| Uruguay | 88538.8 (81835.6-95884.2) | 2826 (2615.4-3048.7) | 97978 (90386.3-106582.2) | 2826.2 (2615.5-3049.1) | 0.00096 (0.00058 to 0.00134) |
| Uzbekistan | 871065 (761125.3-1007188.6) | 4841.8 (4197.4-5671.5) | 1614394.8 (1394911-1895977.1) | 4834.2 (4194.6-5660) | -0.00256 (-0.00339 to -0.00172) |
| Vanuatu | 6111.1 (5399.3-6998.2) | 4980.5 (4379.7-5808.2) | 13865.2 (12224.1-15911.6) | 4993.7 (4390.8-5824.5) | 0.00784 (0.00755 to 0.00813) |
| Venezuela (Bolivarian Republic of) | 795858.6 (703135.2-917285.4) | 4836.2 (4254.6-5575) | 1358610.6 (1188618.1-1579641.2) | 4841.7 (4264-5582.8) | -0.00017 (-0.00126 to 0.00092) |
| Viet Nam | 3195623.4 (2774570.4-3721606.4) | 5444.1 (4729.1-6344.4) | 5752398.6 (4972037.7-6766548.8) | 5430.1 (4718-6324.1) | -0.00835 (-0.00852 to -0.00819) |
| Yemen | 458559.8 (411391.8-517538.2) | 4349.2 (3835.9-4997.5) | 1249385.1 (1113799-1425925.7) | 4340.1 (3827.7-4991.1) | -0.00748 (-0.00946 to -0.0055) |
| Zambia | 310761.2 (278912.6-347372.1) | 4879 (4350.1-5520.3) | 815774.8 (730182.2-910885.8) | 4883.5 (4355-5525.8) | 0.00348 (0.00315 to 0.00381) |
| Zimbabwe | 414164.1 (371693.5-463338.8) | 4894.3 (4366-5538.7) | 672181.1 (601934.7-751088) | 4905.9 (4376.5-5549.5) | 0.00738 (0.0068 to 0.00796) |
|  |  |  |  |  |  |

DALYs

| **location** | **1990** | | **2021** | | **EAPC_**  **95%CI** |
| --- | --- | --- | --- | --- | --- |
|  | **Number_95% UI** | **ASR** | **Number_95% UI** | **ASR** |  |
| Afghanistan | 9671.2 (5569.8-15688.1) | 85.9 (50.9-137.7) | 30914 (17309-50536.3) | 85.8 (50.5-135.4) | 0.00318 (0.00012 to 0.00623) |
| Albania | 3947.3 (2310.9-6422) | 112 (66.9-178.4) | 2579.5 (1582.6-3963.2) | 112.5 (66.2-178.2) | 0.01738 (0.0098 to 0.02495) |
| Algeria | 24747.2 (14040-39365.5) | 86.5 (51.2-135.1) | 38556.1 (22731.7-61310.8) | 86.5 (51.3-136.8) | -0.01285 (-0.02083 to -0.00487) |
| American Samoa | 56.9 (32.9-92) | 107.8 (63-170.6) | 53.5 (31.3-85.6) | 107.5 (63.2-172.6) | -0.00043 (-0.00387 to 0.00301) |
| Andorra | 72.2 (40.1-117.2) | 162.3 (88.9-267.8) | 98.2 (54.9-157.9) | 162.5 (88.5-263.7) | 0.007 (0.00326 to 0.01074) |
| Angola | 7827.8 (4538.9-12318.6) | 69.5 (41.9-106.5) | 25613.2 (14538.3-40662.4) | 70.1 (42.2-107.9) | 0.03039 (0.02681 to 0.03398) |
| Antigua and Barbuda | 70.2 (40.7-112.3) | 115 (66.6-183.1) | 93.3 (55.7-145.4) | 114.6 (66.3-183.4) | -0.01271 (-0.01639 to -0.00902) |
| Argentina | 51467.8 (27187.1-85594.4) | 150.8 (79.9-250.3) | 61618.6 (33087.6-100855.5) | 150.7 (79.9-247.7) | -0.00294 (-0.00606 to 0.00019) |
| Armenia | 8020.8 (4433.4-12946.6) | 226 (126.1-362.8) | 5723.2 (3270.9-9213.2) | 225.9 (125.9-367.2) | -0.00155 (-0.00579 to 0.0027) |
| Australia | 17015 (9245.6-27570.9) | 113.6 (61.2-185.3) | 23780.3 (13158.7-38859.1) | 113.8 (61.9-188.1) | 0.00819 (-0.00207 to 0.01846) |
| Austria | 10386.5 (5835-16787.5) | 162.8 (89.7-266.3) | 11141.7 (6188.1-17970.6) | 162.8 (88.6-265.4) | 0.00184 (-0.00096 to 0.00465) |
| Azerbaijan | 18042.6 (9961.5-29536.4) | 226.4 (126.9-368.8) | 20949.3 (11780.6-33959.5) | 225.1 (125.2-367.4) | -0.01869 (-0.02124 to -0.01613) |
| Bahamas | 299.7 (172.7-482.7) | 115 (66.9-183.7) | 411.8 (244.9-642.4) | 114.9 (66.8-182.6) | -0.00224 (-0.00436 to -0.00012) |
| Bahrain | 441.2 (255.1-710.5) | 86.1 (50.7-137.3) | 1170 (702.2-1846.5) | 85.7 (50.6-138) | -0.0133 (-0.01697 to -0.00963) |
| Bangladesh | 118041.5 (66872.1-188226.2) | 91.3 (53.8-145.3) | 149709.5 (86931.6-240534) | 92 (53.5-147.3) | 0.03504 (0.03117 to 0.03892) |
| Barbados | 281.5 (165.2-448.6) | 115.2 (66.6-184.4) | 307.9 (186.6-464.2) | 114.8 (66-182.4) | -0.01014 (-0.01209 to -0.00818) |
| Belarus | 11306.7 (6757.7-17973.3) | 116.6 (68-187.6) | 9118.7 (5537.3-14128.6) | 116.2 (67.5-184.7) | -0.00197 (-0.00528 to 0.00133) |
| Belgium | 13413.2 (7467.2-21533.8) | 162.8 (89-264.3) | 14851.7 (8345.7-23873.1) | 162.8 (89.9-265.4) | -0.00187 (-0.00455 to 0.00081) |
| Belize | 243.1 (135.9-400.9) | 114.8 (66.1-184.8) | 488.4 (282-783.8) | 114.6 (66.1-183.7) | 0.00085 (-0.00145 to 0.00314) |
| Benin | 3937.8 (2274.2-6323.9) | 72.9 (44.4-113.2) | 10730.9 (6181.1-17255.5) | 73.3 (43.8-112.3) | 0.01597 (0.01365 to 0.01828) |
| Bermuda | 62.8 (36.8-99.1) | 115.3 (66.5-184.2) | 64.6 (39.4-97.5) | 115.1 (66-182.9) | -0.0048 (-0.00766 to -0.00194) |
| Bhutan | 656.2 (373.5-1054) | 91.3 (53.9-144.2) | 656.6 (389.3-1032.3) | 91.7 (54-144.6) | 0.0203 (0.01729 to 0.02331) |
| Bolivia (Plurinational State of) | 7413.3 (4255.9-12080.7) | 107.1 (63.9-172.9) | 12653.8 (7543.8-20215.5) | 107.1 (63.8-169.8) | 0.00893 (0.00555 to 0.01231) |
| Bosnia and Herzegovina | 4747.9 (2823.5-7473.5) | 112.6 (66-178.7) | 3113.1 (1929.1-4733.4) | 112.6 (66.3-177.7) | -0.00254 (-0.00687 to 0.00179) |
| Botswana | 1055.2 (604.2-1706.2) | 72.5 (43.5-112.7) | 1698.8 (997.5-2650.7) | 72 (42.7-112.5) | -0.01138 (-0.01436 to -0.0084) |
| Brazil | 209200.3 (120153.7-335221) | 135.1 (78.4-216.3) | 282228.4 (167551.9-445686) | 135.5 (79.1-217.3) | 0.00963 (0.00427 to 0.01499) |
| Brunei Darussalam | 519.6 (273.3-859.4) | 177.9 (94.3-292.9) | 694 (371.2-1144.2) | 178.1 (93.9-295) | -0.00177 (-0.00778 to 0.00424) |
| Bulgaria | 8746.4 (5336.7-13531.3) | 112.7 (65.9-181.6) | 6369.7 (3979-9687.8) | 112.5 (65.9-178) | -0.0039 (-0.00632 to -0.00148) |
| Burkina Faso | 7697.5 (4464.6-12523.2) | 73 (44.6-113.8) | 18085.3 (10426.1-29084.4) | 73.3 (44.1-113.6) | 0.02895 (0.02447 to 0.03343) |
| Burundi | 4197.7 (2446-6669.3) | 69.9 (42.7-106.9) | 9920.3 (5800.8-15881.6) | 70 (42.7-107) | 0.01179 (0.00699 to 0.01659) |
| Cabo Verde | 285.4 (166.2-452.3) | 73.8 (44.9-113.1) | 398.9 (241.9-617.8) | 73.5 (44.4-113.8) | -0.01356 (-0.0167 to -0.01042) |
| Cambodia | 12451.9 (7136.4-20157.6) | 109.2 (65.4-170.8) | 18727.6 (11202-29451.4) | 108.8 (65.4-170.9) | 0.00017 (-0.00381 to 0.00416) |
| Cameroon | 9522.7 (5437.5-15273) | 82.1 (49-128.8) | 28412.7 (16300.8-45635.9) | 82.4 (49.3-128.2) | 0.03964 (0.03 to 0.04928) |
| Canada | 40165.7 (23353-64245) | 156.1 (89.4-251.3) | 53872.9 (31752.8-85886.7) | 156 (89.5-250.4) | 0.00096 (-0.00122 to 0.00313) |
| Central African Republic | 2069.1 (1187.7-3254.1) | 69.4 (41.7-105.4) | 4106.9 (2397.8-6528.7) | 69.5 (41.8-106.9) | 0.02297 (0.01739 to 0.02855) |
| Chad | 4848.1 (2820.8-7849.9) | 73 (44.3-113.5) | 14501.6 (8394.3-23676.2) | 73 (44.2-113.6) | -0.00237 (-0.00656 to 0.00182) |
| Chile | 20582.1 (10991.2-34143.2) | 150.6 (80.8-249.5) | 23852.1 (12914.5-39649.5) | 150.5 (80.1-252.1) | -0.00298 (-0.00539 to -0.00057) |
| China | 1102173 (663049.9-1730700.1) | 95.7 (57.8-149.7) | 1311104.5 (819799.9-1974814.9) | 95.4 (57.6-148.3) | 0.00839 (-0.00579 to 0.02256) |
| Colombia | 38665.1 (22039.5-61613.2) | 112.1 (64.8-176.3) | 51338.7 (30397.3-80697.8) | 112.3 (65-177.7) | 0.00646 (0.00365 to 0.00927) |
| Comoros | 350.2 (203.3-564.5) | 70 (42.5-109.4) | 527.6 (315.6-823.4) | 70.1 (42.4-107.8) | 0.01577 (0.01177 to 0.01978) |
| Congo | 1576.6 (927.7-2498.4) | 61.3 (37.6-91.9) | 3382.8 (2020.8-5238.9) | 61.2 (37.4-92) | -0.00612 (-0.01169 to -0.00054) |
| Cook Islands | 21.6 (12.3-34.7) | 108 (62.7-171.7) | 18.1 (10.9-28.2) | 108.4 (63.2-172) | 0.01757 (0.01394 to 0.0212) |
| Costa Rica | 3673.1 (2079.2-5985.4) | 112.2 (64.4-180.6) | 4957.2 (2910.5-7894.7) | 112.1 (64.5-179.3) | 0.0027 (4e-05 to 0.00536) |
| Croatia | 4892.9 (3003.8-7582.5) | 113 (67-180.4) | 3940.5 (2488.8-6052) | 112.8 (66.4-179.7) | -8e-04 (-0.00356 to 0.00196) |
| Cuba | 11850.2 (6998.4-18830.7) | 114.7 (67.5-183.4) | 11615.8 (7006.7-17724) | 114.6 (65.8-182.2) | 0.006 (0.00288 to 0.00912) |
| Cyprus | 1087.2 (590.3-1758.5) | 148.2 (79.9-240) | 1574.4 (878.8-2519.2) | 148.2 (79.9-240.7) | -0.00787 (-0.01487 to -0.00087) |
| Czechia | 10453.1 (6357.2-16338.5) | 112.7 (66.5-178) | 10196.8 (6295.6-15413.5) | 112.5 (67-179.3) | -0.00524 (-0.00729 to -0.00318) |
| Côte d'Ivoire | 9673.6 (5539.2-15736.3) | 72.5 (43.3-113.4) | 21656.9 (12760.3-35096.2) | 73.1 (44.4-114.9) | 0.0232 (0.01948 to 0.02692) |
| Democratic People's Republic of Korea | 22492.5 (13116.8-36224.7) | 109.6 (64-175.5) | 26455 (15971-40718.6) | 109.1 (64.4-173.7) | -0.01209 (-0.01473 to -0.00945) |
| Democratic Republic of the Congo | 29139.3 (16511.3-46680.2) | 69.1 (41.6-105.2) | 68062.7 (39326.2-107053.8) | 69.7 (42.2-107.4) | 0.02968 (0.02448 to 0.03488) |
| Denmark | 8156.2 (4397-13232.1) | 195.8 (104.5-322.1) | 9058.4 (4900.9-14865.9) | 196.2 (104.7-324.4) | 0.04829 (0.02884 to 0.06774) |
| Djibouti | 304.6 (176.9-485.6) | 69.7 (42-107.8) | 882.6 (525.4-1378.4) | 69.9 (42.1-106.8) | -0.00064 (-0.00417 to 0.0029) |
| Dominica | 87.2 (49.2-142.2) | 114.8 (65.8-185) | 71.3 (42.4-113.3) | 114.4 (66-184.1) | -0.01476 (-0.01758 to -0.01194) |
| Dominican Republic | 8803.5 (4999.8-14187.5) | 114.9 (66.7-183.8) | 12363.2 (7237.5-19649.8) | 114.7 (66.8-182.9) | -0.00726 (-0.00993 to -0.00458) |
| Ecuador | 11504.3 (6644-18462.4) | 108.7 (65.1-171.6) | 19532.9 (11509.5-30973.2) | 108.8 (64.4-172.5) | 0.01007 (0.0047 to 0.01545) |
| Egypt | 38205.7 (22714.1-60335.4) | 64.8 (39.7-98.8) | 70249.2 (43038.5-109440.6) | 64.4 (40.5-97.9) | -0.04763 (-0.06439 to -0.03087) |
| El Salvador | 6567.8 (3693.4-10540.5) | 111.7 (64.8-177) | 7226.5 (4178.8-11485.5) | 112.2 (65.2-178.7) | 0.01637 (0.0123 to 0.02044) |
| Equatorial Guinea | 322.9 (185.9-502.3) | 69.1 (41.5-105) | 1111.4 (644.8-1783.2) | 69.4 (41.6-106.7) | 0.01666 (0.00934 to 0.02397) |
| Eritrea | 2550.2 (1471.7-4071.8) | 69.5 (42.3-107.3) | 4782.2 (2854.6-7470.8) | 69.9 (42.6-106.2) | 0.01887 (0.01584 to 0.02189) |
| Estonia | 2982.4 (1687.7-4838) | 212.4 (119-344.3) | 2175.8 (1255.2-3522.4) | 211.7 (117.8-341.4) | -0.02711 (-0.03869 to -0.01553) |
| Eswatini | 658.8 (373.1-1059.8) | 72.7 (43.5-113) | 863.5 (504.5-1379.4) | 72 (43.2-112.4) | -0.03343 (-0.03687 to -0.03) |
| Ethiopia | 36491.3 (21612.6-57795.5) | 68.4 (42.5-104.6) | 76953 (45987.8-122412.7) | 68.9 (42.7-105.6) | 0.06911 (0.05473 to 0.08348) |
| Fiji | 878.6 (502.5-1407.2) | 107.8 (62.6-169.8) | 1001.3 (584.4-1585.4) | 107.8 (63-170.1) | 0.00517 (0.0018 to 0.00855) |
| Finland | 6837.9 (3793.4-11181.5) | 162.7 (88.9-268.7) | 6971.8 (3908-11233) | 162.8 (88.3-266.9) | 0.00077 (-0.00198 to 0.00352) |
| France | 120799.5 (67846.2-195382.1) | 234.9 (129.9-383.9) | 130651.4 (73382.8-209413.2) | 234.9 (128.5-384.7) | -0.03047 (-0.04885 to -0.01208) |
| Gabon | 913.9 (525.5-1471.6) | 83.9 (49.4-132.8) | 1620.2 (943-2566.7) | 84.3 (49.5-132.4) | -0.00104 (-0.021 to 0.01893) |
| Gambia | 783.1 (458.1-1253.7) | 73.1 (44.5-113.3) | 1864 (1084.3-3005.1) | 73.3 (44.1-115.2) | 0.01054 (0.00755 to 0.01353) |
| Georgia | 11684.8 (6538.9-18802.6) | 224.6 (124.8-361.6) | 6989.9 (3971.1-11346) | 224.8 (125.2-368.2) | 0.02262 (0.01043 to 0.03481) |
| Germany | 112679 (63243.6-179591) | 174.1 (96-280.5) | 114161.8 (64536.7-181552.4) | 173.4 (95.4-280.8) | -0.01813 (-0.06958 to 0.03335) |
| Ghana | 10402.8 (6191.5-16560) | 65.4 (40.2-99.5) | 23152.3 (13759.4-36473.1) | 65.8 (40.1-101.1) | 0.06326 (0.00261 to 0.12396) |
| Greece | 12599.1 (6978.5-20103.2) | 144.3 (78.7-233.2) | 10859.8 (6131.4-17100.8) | 145.2 (79.5-233) | 0.03468 (0.02678 to 0.04259) |
| Greenland | 82.9 (47.4-133.6) | 153.9 (89.1-248.5) | 81.5 (46.9-131.2) | 154.5 (88.3-251) | 0.024 (0.02048 to 0.02752) |
| Grenada | 109 (61.5-176.4) | 114.7 (66.6-184.1) | 109.5 (64.4-171.8) | 114.2 (66.4-183.1) | -0.01393 (-0.01842 to -0.00945) |
| Guam | 148.5 (86.4-240.9) | 107.9 (62.3-173.5) | 163.7 (97.7-257.8) | 108.1 (63.6-174.6) | 0.00578 (0.00134 to 0.01022) |
| Guatemala | 11071.5 (6135.6-18140.2) | 111.4 (63.7-177.1) | 18148.9 (10418.3-29207.7) | 111.9 (64.9-178.5) | 0.01587 (0.01384 to 0.0179) |
| Guinea | 4770.2 (2810-7657.4) | 73.1 (45-114.4) | 10689.9 (6255.2-17171.7) | 73.4 (44.8-112.9) | 0.00991 (0.00642 to 0.01341) |
| Guinea-Bissau | 806.4 (471.8-1309.9) | 73 (44.3-113.8) | 1624.6 (950.8-2636.6) | 73.3 (44.4-114.6) | 0.01815 (0.01518 to 0.02113) |
| Guyana | 948.7 (537.1-1537.1) | 114.1 (66.7-183.3) | 863.9 (503.7-1360.4) | 113.9 (66.4-179.3) | 0.0035 (-0.00011 to 0.00711) |
| Haiti | 8073.1 (4532.1-13146.2) | 113.7 (66.3-181.3) | 15142.5 (8598.9-24208.1) | 113.7 (65.7-181.1) | 0.00045 (-0.00319 to 0.00409) |
| Honduras | 6169.7 (3403.7-9986.5) | 111.8 (64.4-179.2) | 11682.8 (6654.6-18909.4) | 111.9 (64.3-178.7) | 0.00838 (0.0055 to 0.01125) |
| Hungary | 13495.3 (7890-21355.5) | 148.6 (84.4-240.2) | 11386.8 (6865.5-17402.9) | 148.4 (84.9-236) | -0.03902 (-0.06297 to -0.01506) |
| Iceland | 435.5 (237.9-704.1) | 181.9 (98.9-295.3) | 531.4 (292.8-853.4) | 180.7 (97.6-294.2) | -0.03085 (-0.04048 to -0.02122) |
| India | 874229.5 (507470-1397894.5) | 93.5 (55.7-147.7) | 1284991.9 (772882.4-2038272.1) | 94.1 (56.1-149.4) | 0.00533 (-0.0062 to 0.01686) |
| Indonesia | 217288.3 (128426.4-347017) | 112.6 (67.4-175.8) | 307069.5 (187020.8-472663.5) | 113.4 (68.4-177) | 0.03483 (0.02897 to 0.04069) |
| Iran (Islamic Republic of) | 63298.3 (36189.7-102814.9) | 96.7 (57.6-153.2) | 79271.4 (46961.8-123997.3) | 97.7 (57.7-154.9) | 0.05951 (0.04875 to 0.07028) |
| Iraq | 18221.2 (10490.4-29745.5) | 86.2 (51-136.3) | 36674.4 (21159.9-58192.8) | 86.1 (50.6-135.4) | 0.00102 (-0.00233 to 0.00437) |
| Ireland | 5743.1 (3155.3-9476) | 162.7 (89.1-268.4) | 6845.7 (3793.3-11194.3) | 162.7 (88.7-267.3) | 0.00396 (0.00093 to 0.00698) |
| Israel | 8414.1 (4603.6-13528.8) | 163.1 (89.3-262.2) | 15316.2 (8424.5-25108.1) | 163.1 (89.7-267.8) | 0.00127 (-0.00079 to 0.00333) |
| Italy | 92673.1 (52153.9-149537.2) | 200.8 (109.8-327.9) | 89283.9 (50125.8-143290.2) | 200.7 (109.1-328.4) | -0.00896 (-0.01594 to -0.00197) |
| Jamaica | 2885.6 (1642.1-4745) | 115.1 (66.7-184.9) | 2979.7 (1770.1-4660.8) | 114.7 (66.9-182.9) | -0.00572 (-0.00807 to -0.00337) |
| Japan | 232220.1 (124105.6-377886.8) | 225.6 (119.2-370.9) | 201309.7 (109559.7-325307.3) | 226.2 (119-371.8) | -0.02232 (-0.05942 to 0.01479) |
| Jordan | 3634.3 (2056.4-5741.3) | 85.5 (50.9-131.4) | 10522.2 (6209-16556.3) | 85.4 (50.3-133.6) | -0.0072 (-0.01313 to -0.00127) |
| Kazakhstan | 39018 (21504.3-63284.6) | 225.9 (125.7-365.1) | 42430.5 (23458.5-69025.7) | 226.3 (125.1-367.6) | 0.01161 (0.00931 to 0.0139) |
| Kenya | 15881.3 (9394-25474.4) | 66 (41.1-100.4) | 34633.6 (20946.3-55155.7) | 68.2 (42.3-104.5) | 0.15752 (0.13512 to 0.17992) |
| Kiribati | 87.6 (49.9-140.9) | 107.6 (63.2-169.8) | 137.2 (78.4-220.3) | 107.9 (62.9-171.5) | 0.01404 (0.01109 to 0.01699) |
| Kuwait | 1588.4 (912.9-2518.6) | 90.1 (53.2-140.5) | 3650 (2185.9-5492.7) | 89.8 (52.1-138.6) | -0.03078 (-0.04056 to -0.02099) |
| Kyrgyzstan | 11722.7 (6414.8-19124.7) | 226.3 (125.7-368.1) | 16648 (9148.6-27091.2) | 226.1 (125.5-367.2) | 0.00614 (0.00335 to 0.00894) |
| Lao People's Democratic Republic | 5011.6 (2870.2-8065.8) | 109.1 (64-170.2) | 8188.7 (4855.2-12984.2) | 109.1 (64.8-171.2) | 0.00581 (0.00303 to 0.00858) |
| Latvia | 1770.4 (1134.7-2683.1) | 69.8 (43.6-107.9) | 1205.5 (761.4-1805.9) | 69.5 (43.4-106.1) | -0.01091 (-0.01574 to -0.00608) |
| Lebanon | 2724.9 (1590.4-4363.8) | 86.4 (51.2-136.3) | 4505.8 (2671.5-7072.7) | 86.4 (50.3-137.1) | 0.00056 (-0.00367 to 0.00478) |
| Lesotho | 1229.5 (708.5-1958.4) | 72.8 (43.8-113) | 1387.4 (815-2215) | 72 (43.1-112.5) | -0.03344 (-0.0359 to -0.03098) |
| Liberia | 1942.8 (1125.6-3098.7) | 72.2 (43.6-112.5) | 4172.9 (2445.8-6621.9) | 72.5 (43.9-111) | 0.01453 (0.01031 to 0.01874) |
| Libya | 4140.3 (2342.9-6832.4) | 86.7 (50.8-137.8) | 5475 (3243.3-8576.3) | 86.4 (50.4-137.6) | -0.0108 (-0.01354 to -0.00805) |
| Lithuania | 3951.6 (2351.5-6292.7) | 116.6 (68.6-186.8) | 2622 (1617.1-4000.5) | 116.1 (68.4-187.4) | -0.00904 (-0.01235 to -0.00573) |
| Luxembourg | 503.7 (281.3-804.4) | 162.7 (89-264.3) | 815.8 (453.9-1317.4) | 162.6 (88.8-266.1) | 0.00141 (-0.00071 to 0.00353) |
| Madagascar | 8929.1 (5187-13943) | 69.6 (42.3-106.7) | 21179.7 (12387.5-33984.7) | 70.1 (42.7-109.7) | 0.0294 (0.02563 to 0.03318) |
| Malawi | 7281.5 (4217.7-11506.2) | 69.4 (42.2-105.3) | 14486.4 (8406.7-23286.8) | 70 (42.3-108.8) | 0.0423 (0.03707 to 0.04754) |
| Malaysia | 20361.9 (11875.2-32325.2) | 108.6 (64.3-167.9) | 33425.8 (19974.1-52245) | 108.5 (63.9-171.7) | -0.00796 (-0.01035 to -0.00557) |
| Maldives | 272.2 (157.2-443.5) | 107.9 (64.9-171.3) | 502.3 (304-760.3) | 106.5 (63.2-167.9) | -0.05604 (-0.07437 to -0.03772) |
| Mali | 7398.8 (4235.2-11981.3) | 77.5 (46.8-119.6) | 20721.1 (12110-33187.7) | 77.4 (46.9-120.4) | -0.00271 (-0.00792 to 0.0025) |
| Malta | 550.8 (304.6-885.1) | 162.9 (89.3-263.6) | 548.7 (309.1-889) | 162.6 (89.3-267.5) | -0.00676 (-0.00879 to -0.00472) |
| Marshall Islands | 57.7 (31.8-93.9) | 107.6 (63-172.3) | 61.5 (35.8-98.4) | 107.5 (63-170.6) | -0.00412 (-0.006 to -0.00223) |
| Mauritania | 1631.1 (937.9-2584.8) | 73.2 (44.4-112.3) | 3471 (2027.9-5499.1) | 73.6 (44.4-113.6) | 0.01531 (0.01236 to 0.01826) |
| Mauritius | 1198.4 (710.9-1905.1) | 108.6 (64.6-171.5) | 1263.9 (786.3-1913.4) | 108.6 (65.2-168.4) | -0.00229 (-0.00528 to 7e-04) |
| Mexico | 94533.5 (54620.6-152354.9) | 103.6 (61.9-164.9) | 128083.6 (77256.6-200189.1) | 102.5 (61.4-162) | -0.05201 (-0.05916 to -0.04486) |
| Micronesia (Federated States of) | 127.2 (71.5-204.1) | 107.6 (62.9-170.9) | 111.8 (65.1-179.5) | 107.8 (63.4-171.3) | 0.00037 (-0.00256 to 0.0033) |
| Monaco | 35.5 (19.9-56.9) | 162.8 (88.2-265.5) | 45.6 (25.8-73.5) | 163.1 (89.9-266) | 0.01184 (0.00857 to 0.01511) |
| Mongolia | 5970.1 (3239.8-9818.3) | 225.3 (126.2-367.9) | 8007 (4433.1-13089.6) | 226.1 (127-368.1) | 0.01398 (0.01209 to 0.01588) |
| Montenegro | 678.9 (407.4-1069.5) | 112.9 (67.3-178.7) | 607.8 (372.8-938.2) | 112.6 (66.2-178.9) | -0.00302 (-0.00561 to -0.00043) |
| Morocco | 23898.1 (13806.6-38263) | 86.7 (51.4-137.1) | 31357.9 (18742.2-49813.5) | 86.5 (51.2-137.7) | -0.00491 (-0.00657 to -0.00326) |
| Mozambique | 10004.2 (5804.9-16011.7) | 69.4 (42.4-106.4) | 23505.2 (13678.2-37631.4) | 69.5 (42.4-106.8) | 0.02283 (0.01571 to 0.02996) |
| Myanmar | 46481.6 (27123.3-73662) | 108.7 (64.5-168.4) | 61323.9 (36436.1-96224.6) | 109.3 (64.6-171.2) | 0.02421 (0.02076 to 0.02766) |
| Namibia | 1106.2 (635.3-1766.6) | 72.4 (43.6-112.3) | 1806.6 (1065.3-2851.7) | 72.4 (43.3-113.4) | 0.00551 (0.00256 to 0.00846) |
| Nauru | 12.2 (6.9-19.9) | 107.8 (62.8-173.4) | 12.7 (7.3-20.4) | 107.9 (62.8-172) | 0.00666 (0.0042 to 0.00911) |
| Nepal | 20656.6 (11703.9-33183.6) | 92 (54.5-144.6) | 29204.6 (17149.8-46275.1) | 92.9 (55-146.4) | 0.03309 (0.03009 to 0.03609) |
| Netherlands | 20386.5 (11328.7-33080.8) | 163.5 (89.5-268.2) | 21618.4 (12095.3-34854.4) | 161.7 (88.2-266.6) | -0.02173 (-0.03205 to -0.0114) |
| New Zealand | 4883.3 (2651.7-7943) | 157.2 (84.4-257.6) | 6773.2 (3734.1-11004.7) | 158.6 (85.6-260.1) | 0.0435 (0.03384 to 0.05316) |
| Nicaragua | 5102.5 (2830.8-8228.3) | 111.9 (65-178) | 7512.4 (4257.8-12009) | 112 (64-178) | 0.0054 (0.00247 to 0.00832) |
| Niger | 6473.2 (3744.9-10399.7) | 72.8 (44.2-112.6) | 20573.4 (11890.3-33610.4) | 73.4 (44.7-114.4) | 0.02578 (0.02237 to 0.0292) |
| Nigeria | 72890.3 (43300.9-116180.2) | 77.1 (47.6-119.3) | 189098.6 (112112.6-301513.7) | 77.6 (47.9-119.2) | 0.03202 (0.02725 to 0.03679) |
| Niue | 2.7 (1.5-4.3) | 108.1 (63.4-172.9) | 1.7 (1-2.7) | 107.8 (63.6-172.5) | -0.00369 (-0.00657 to -0.00081) |
| North Macedonia | 2165.1 (1296.7-3381.7) | 112.6 (66.9-176.1) | 2019.1 (1245.4-3069.9) | 112.1 (65.9-178.2) | -0.00893 (-0.01217 to -0.00569) |
| Northern Mariana Islands | 47.1 (27.6-75.2) | 108.2 (63-172) | 49.5 (29.6-77.2) | 107.8 (63-171.4) | -0.01057 (-0.02075 to -0.00038) |
| Norway | 6497.5 (3614.1-10508.7) | 180 (98.1-292.3) | 7974.4 (4438.3-12763.3) | 181.1 (98.5-294.9) | 0.0322 (0.00824 to 0.05616) |
| Oman | 1868.9 (1077.2-2987.3) | 84.7 (50.7-132.2) | 3777.9 (2223.1-5855.1) | 84.7 (50.4-132.9) | -0.01919 (-0.03348 to -0.00489) |
| Pakistan | 120663.1 (68522.5-191993) | 94 (55.2-148.3) | 237554.9 (138105.6-381552.1) | 94.3 (56-149.6) | 0.013 (0.01116 to 0.01485) |
| Palau | 16.5 (9.6-26.6) | 107.9 (63-172.4) | 17.4 (10.6-26.3) | 107 (62.6-169.5) | -0.02681 (-0.0326 to -0.02103) |
| Palestine | 2167.4 (1213.9-3504.8) | 90 (53.3-142.1) | 4882.9 (2820.2-7775.8) | 88.8 (52.5-139.9) | -0.02272 (-0.03521 to -0.01023) |
| Panama | 2830.9 (1603.8-4554.6) | 112.1 (64.7-177.6) | 4736.2 (2732.3-7487.8) | 111.9 (64.4-177.2) | 0.00166 (-0.00081 to 0.00414) |
| Papua New Guinea | 4870.1 (2793.4-7815.3) | 106.9 (63.7-170.5) | 12029.4 (6961.8-19403.7) | 107.3 (63.4-170.8) | 0.00958 (0.00683 to 0.01234) |
| Paraguay | 6034.4 (3335.1-9728.1) | 134.6 (77.7-214.4) | 9528.7 (5442.4-15396.7) | 134.5 (76.8-217.2) | 0.00013 (-0.00218 to 0.00244) |
| Peru | 24886.6 (14489.8-39977.8) | 108.9 (64.9-170.8) | 38767.7 (23203.7-59484.9) | 109 (64.9-168.5) | 0.0186 (0.00425 to 0.03295) |
| Philippines | 75514.8 (44680.1-121858.1) | 112.8 (68.4-176.3) | 128742.5 (77602.2-202344.3) | 113 (68.4-175.1) | 0.0076 (0.0056 to 0.00959) |
| Poland | 42728.3 (25961.8-66901.2) | 117 (70.3-187.1) | 38486.9 (24243-58822.3) | 117 (70.1-187.4) | 0.00021 (-0.00186 to 0.00227) |
| Portugal | 14301.2 (7889-23068.8) | 162.6 (88.3-263.3) | 12689 (7145.8-20344.4) | 163 (89.3-267) | 0.00807 (0.00533 to 0.01082) |
| Puerto Rico | 4104.7 (2409.2-6553.8) | 115 (67.3-184.2) | 3349.9 (2052.1-5131.8) | 114.7 (66.6-182.8) | -0.00133 (-0.00373 to 0.00106) |
| Qatar | 365 (210.2-586.1) | 85.4 (50.4-134.1) | 2131.8 (1279.4-3275.7) | 85.4 (50.4-135.4) | -0.01012 (-0.01428 to -0.00597) |
| Republic of Korea | 80114.1 (42585.3-132120.5) | 188.5 (100.1-309) | 66718.8 (36505.4-109054.4) | 188.8 (100.3-314.1) | 0.02664 (0.00991 to 0.04337) |
| Republic of Moldova | 5140.1 (3017.6-8217.1) | 116.7 (68.2-186.8) | 3346.3 (2059-5182) | 116.1 (67.9-180.9) | -0.01245 (-0.01556 to -0.00934) |
| Romania | 19305.6 (12110.6-29968.6) | 86.3 (53.4-133.7) | 14827.2 (9478.5-22278.2) | 87 (53.1-133.1) | 0.04588 (0.03712 to 0.05464) |
| Russian Federation | 238962.8 (136726.5-381560.6) | 174.7 (98.1-279.8) | 206924.8 (120076.6-330937.9) | 178.4 (99.7-287) | 0.11946 (0.08041 to 0.15852) |
| Rwanda | 4290.5 (2515.1-6701) | 56.9 (35.1-84.7) | 7664.5 (4635.8-11862.1) | 57.1 (35.8-85.2) | 0.00925 (-0.01443 to 0.03294) |
| Saint Kitts and Nevis | 50.2 (28.7-81.8) | 114.7 (66.3-185.4) | 60 (36-93.5) | 114.6 (66.7-185.3) | 0.00067 (-0.00143 to 0.00277) |
| Saint Lucia | 169.5 (96.2-274.7) | 114.8 (66.7-184.1) | 181.7 (108.4-280.7) | 114.4 (66-182.1) | -0.01053 (-0.01316 to -0.00791) |
| Saint Vincent and the Grenadines | 135.7 (76.9-216.3) | 114.7 (66.3-182.1) | 123.2 (73.3-192) | 114.3 (66.8-183.1) | -0.00833 (-0.01127 to -0.0054) |
| Samoa | 204 (114.2-330.6) | 107.7 (62.4-171.4) | 248.6 (144.2-397.6) | 107.8 (63.8-170.2) | 0.00598 (0.0033 to 0.00866) |
| San Marino | 31.6 (17.6-51.4) | 163.3 (88.8-267.9) | 39.5 (22.1-63) | 162.9 (88.3-267.1) | -0.00922 (-0.01109 to -0.00736) |
| Sao Tome and Principe | 98.1 (56.2-158.3) | 73.6 (44.6-114.6) | 164.2 (98.3-260) | 73.5 (44.6-114.2) | 0.00392 (0.00035 to 0.00749) |
| Saudi Arabia | 15205.9 (8653.4-24675.8) | 85.9 (50.3-135.6) | 28938.8 (17417.3-44471.2) | 85.8 (51.3-134.8) | -0.00177 (-0.00621 to 0.00268) |
| Senegal | 6125.9 (3526-9754.4) | 73 (44.5-112.6) | 12256.3 (7197.6-19612.7) | 73.1 (44.4-113.1) | 0.01226 (0.00893 to 0.01559) |
| Serbia | 9997.9 (6020.4-15547.6) | 113.3 (66.9-179.6) | 8314.8 (5115.7-12666) | 112.4 (66.1-178.7) | -0.02533 (-0.02934 to -0.02131) |
| Seychelles | 82.2 (48.7-130) | 109.1 (65.5-169.5) | 108.4 (66-165.3) | 108.3 (64.4-170) | -0.03401 (-0.03857 to -0.02944) |
| Sierra Leone | 3239.4 (1906.5-5221.5) | 72.8 (44.3-114.3) | 6848.2 (4054.5-10948.3) | 73.1 (44.6-114.4) | 0.01522 (0.01036 to 0.02008) |
| Singapore | 4061.3 (2215.4-6598.2) | 154.8 (84-253.9) | 6321.9 (3435.7-10237) | 153.5 (81.1-252) | -0.03815 (-0.04741 to -0.02889) |
| Slovakia | 5659.3 (3392.2-8992) | 113 (66.8-179.6) | 5137.7 (3195.9-7777.1) | 112.5 (66-178.2) | -0.00595 (-0.00905 to -0.00285) |
| Slovenia | 1995.2 (1214-3126.1) | 112.8 (66.5-180.8) | 1952.8 (1211.3-2957.4) | 112.3 (66.1-177.7) | -0.01296 (-0.01714 to -0.00879) |
| Solomon Islands | 419.9 (235.1-677.4) | 107.6 (63.1-170.7) | 795.3 (452-1288.6) | 107.7 (63.1-171.7) | 0.01018 (0.00773 to 0.01263) |
| Somalia | 5969.9 (3418.3-9406.4) | 69.4 (42.4-106.4) | 16350 (9451.4-26064.2) | 69.7 (42.3-106.8) | 0.0186 (0.01505 to 0.02215) |
| South Africa | 28739.2 (17117.3-45559.2) | 75.1 (46-115.6) | 41500.7 (25354.9-64150.9) | 74.8 (45.9-115.8) | -0.01033 (-0.01214 to -0.00852) |
| South Sudan | 4358.6 (2564-6978.1) | 69 (42.5-106.2) | 7195.5 (4215-11580.5) | 69.4 (42.4-107.1) | 0.02144 (0.018 to 0.02489) |
| Spain | 51194.4 (28173.7-81423.8) | 154.4 (83.1-248.2) | 52264.9 (29493.9-83785.9) | 154 (83.4-251.7) | -0.00086 (-0.00553 to 0.00381) |
| Sri Lanka | 20175.8 (12088.7-31615.9) | 114.3 (69.5-176.5) | 24431.5 (15146.8-37593.8) | 113.9 (69-176.9) | -0.02232 (-0.03023 to -0.01441) |
| Sudan | 19548.1 (11155.1-31276.4) | 86.2 (51.3-134.3) | 40568.8 (23380.1-65130.8) | 86.2 (50.9-136.5) | 0.00765 (0.0051 to 0.01019) |
| Suriname | 462.4 (263.1-751.4) | 114.6 (65.9-183.5) | 637.7 (373.6-1007.1) | 114.2 (65.9-182.4) | -0.00466 (-0.00737 to -0.00196) |
| Sweden | 11711.6 (6476.3-18904.2) | 164.7 (89.2-267.3) | 13969.5 (7739.8-22568.8) | 164.7 (89.3-266.8) | -0.00306 (-0.00569 to -0.00044) |
| Switzerland | 9006.4 (5034.3-14635.3) | 162.5 (88.7-267) | 11123 (6206.7-17994.3) | 162.5 (89.2-265.8) | 0.00232 (-0.001 to 0.00565) |
| Syrian Arab Republic | 12799.8 (7215.4-20860.3) | 86.4 (50.8-136.4) | 11859.8 (6958-18702.9) | 86.6 (50.5-137.4) | 0.0043 (0.00155 to 0.00704) |
| Taiwan (Province of China) | 26983.2 (15702.2-43198.9) | 134.2 (77.5-214.5) | 27759.1 (16713.6-42768.9) | 136.4 (78.6-216.5) | 0.13625 (0.09607 to 0.17645) |
| Tajikistan | 15258.7 (8277.5-25321.9) | 225.7 (125.7-367.9) | 25454.9 (14038.4-41861.5) | 225.4 (125.6-367.8) | 0.00165 (-0.00075 to 0.00405) |
| Thailand | 62639.2 (37418.9-98310.7) | 109.1 (65.4-170.4) | 65744.9 (40801-101341.9) | 109.4 (65.5-172.5) | 0.01299 (0.01053 to 0.01545) |
| Timor-Leste | 916.2 (531.3-1488.4) | 107.7 (64.7-169.1) | 1620.2 (946.5-2595.6) | 108.7 (64.7-170.2) | 0.04186 (0.03658 to 0.04714) |
| Togo | 2868.3 (1646.3-4614.1) | 71.5 (43.4-111.5) | 6311.6 (3721.7-10057.8) | 71.8 (43.7-110.1) | 0.00351 (-0.00058 to 0.0076) |
| Tokelau | 1.9 (1.1-3) | 108.2 (62.9-170.8) | 1.5 (0.9-2.3) | 107.7 (62.5-169.2) | -0.01259 (-0.01779 to -0.00738) |
| Tonga | 120.1 (68.2-195.6) | 108.2 (64.4-174) | 123.6 (71.1-198.2) | 108.3 (63.2-170.4) | -0.00015 (-0.00375 to 0.00346) |
| Trinidad and Tobago | 1447.9 (829.7-2339.6) | 114.6 (66.7-183) | 1468 (867.9-2291.4) | 114.3 (66-183.1) | -0.00405 (-0.00695 to -0.00115) |
| Tunisia | 7804.1 (4541-12506.6) | 86.6 (51.4-137.9) | 9724.8 (5848.5-14967.5) | 86.5 (51.4-135) | 0.00287 (-0.00116 to 0.0069) |
| Turkey | 75716.4 (42563.1-123408.5) | 121.5 (69.6-196.4) | 94683.8 (54732.4-151146.3) | 122.1 (69.7-196.2) | 0.00407 (-0.00895 to 0.01708) |
| Turkmenistan | 10111.9 (5514.6-16743.6) | 225.8 (126.7-366.2) | 11754.2 (6501.9-19278.8) | 224.7 (124.3-368.7) | -0.0116 (-0.0137 to -0.00949) |
| Tuvalu | 11 (6.2-17.8) | 108.6 (63.3-174.1) | 13.5 (7.9-21.6) | 107.7 (62.9-171.9) | -0.01228 (-0.01731 to -0.00726) |
| Uganda | 15752.7 (8790.1-25652.9) | 82.5 (48.5-129.3) | 39773.5 (22656-63981) | 83.2 (49.8-129.3) | 0.02938 (0.01905 to 0.0397) |
| Ukraine | 58563.6 (35547.4-91716.5) | 120.7 (70.4-193.2) | 42688.8 (26563.9-65408.4) | 120.3 (70.6-188.7) | -0.00259 (-0.00593 to 0.00075) |
| United Arab Emirates | 1602.6 (912.2-2558.6) | 85.5 (50.5-134.7) | 6672.5 (4001.4-10081.2) | 85.3 (50.1-134.6) | -0.00897 (-0.01434 to -0.00361) |
| United Kingdom | 89783 (49302.2-145488.7) | 185.4 (100.1-302.5) | 100296.8 (56038.5-162486.1) | 182.3 (99.5-298.2) | -0.07813 (-0.11977 to -0.03647) |
| United Republic of Tanzania | 25645.8 (14684.2-40861.7) | 88.7 (51.9-138.4) | 57146.4 (32041.3-91535.6) | 89.2 (52.3-141.8) | -0.00276 (-0.02247 to 0.01696) |
| United States Virgin Islands | 122.4 (70.4-198.4) | 115.1 (66.7-186.7) | 89.4 (54.3-135.3) | 114.5 (67.3-182.4) | -0.01436 (-0.01676 to -0.01196) |
| United States of America | 471089.7 (284811.6-741458.5) | 192.4 (114.4-306.4) | 539952.3 (317330.8-858834.4) | 174.9 (101.3-279.9) | -0.15908 (-0.20684 to -0.1113) |
| Uruguay | 4521.9 (2405.5-7435.4) | 150.9 (79.7-248.9) | 4357.9 (2354.4-7098.1) | 150.7 (80.4-249.6) | 0.00033 (-0.00248 to 0.00314) |
| Uzbekistan | 57541.1 (31003.9-94683.5) | 225.6 (126-364.1) | 78137.7 (43386.7-127831.4) | 225.3 (125.3-366.9) | 0.00141 (-0.00076 to 0.00359) |
| Vanuatu | 186.7 (106.3-299.9) | 107.7 (63.5-171.2) | 364.2 (208.4-585.9) | 108.1 (62.9-172.6) | 0.00953 (0.00728 to 0.01178) |
| Venezuela (Bolivarian Republic of) | 27308.3 (15337.6-44219.6) | 132.9 (76.4-213.3) | 33775.5 (19606.7-54263.9) | 132.7 (76.1-213.4) | -0.01149 (-0.0162 to -0.00677) |
| Viet Nam | 80287 (46453.6-129091) | 109.3 (64.6-171.7) | 106098.5 (63819.1-165584.9) | 109.2 (65-172.4) | -0.00363 (-0.00611 to -0.00115) |
| Yemen | 14067.4 (7869.7-23178.2) | 85.7 (50.6-134.9) | 31866.4 (18445.7-52457.2) | 85.7 (51-136.5) | 0.00629 (0.00281 to 0.00977) |
| Zambia | 5982.9 (3465.2-9506.7) | 69.7 (42.4-106.7) | 14490.8 (8507.7-23375.5) | 69.7 (42.8-107.4) | 0.02018 (0.01461 to 0.02576) |
| Zimbabwe | 8371.4 (4697.8-13609) | 72.4 (42.9-113.2) | 12088 (6984.7-19221.2) | 72.3 (43.5-110.7) | 0.00262 (-3e-05 to 0.00526) |
|  |  |  |  |  |  |
|  |  |  |  |  |  |
