# Supplementary material for: Trends and projections of dermatitis burden (1990–2040): a 2021 global burden of disease analysis
Source: Front Med (Lausanne). 2026 Jan 28;13:1696683. doi: 10.3389/fmed.2026.1696683 (PMC12891110; doi:10.3389/fmed.2026.1696683)
Supplement: Supplementary file 4 [file Table_4.DOCX]

Supplementary Table S4. SII and CIX Values for Dermatitis Metrics (1990–2021).

| Metric | Year | SII | CIX | |
| --- | --- | --- | --- | --- |
| ASPR | 1990 | 1213.98 | -0.02(-0.286-0.186) |  |
| ASPR | 2021 | 930.66 | 0(-0.181-0.181) |  |
| ASIR | 1990 | 241.79 | 0.05(-0.212-0.245) |  |
| ASIR | 2021 | 450.13 | 0.05(-0.12-0.237) |  |
| ASDR | 1990 | 50.70 | -0.03(-0.296-0.179) |  |
| ASDR | 2021 | 34.58 | -0.01(-0.186-0.174) |  |
