# Supplementary material for: Trends and projections of dermatitis burden (1990–2040): a 2021 global burden of disease analysis
Source: Front Med (Lausanne). 2026 Jan 28;13:1696683. doi: 10.3389/fmed.2026.1696683 (PMC12891110; doi:10.3389/fmed.2026.1696683)
Supplement: Supplementary file 5 [file Table_5.DOCX]

Supplementary Table S5. Exploratory projections of global dermatitis burden using the BAPC model (2021–2040).

| Sex | Year | ASR | Lower | Upper |
| --- | --- | --- | --- | --- |
| ASIR | | | | |
| Male | 1990 | 4593.120296 | 4592.25425 | 4593.986 |
| Male | 1991 | 4576.430672 | 4575.57716 | 4577.284 |
| Male | 1992 | 4561.225001 | 4560.382149 | 4562.068 |
| Male | 1993 | 4549.376029 | 4548.542961 | 4550.209 |
| Male | 1994 | 4542.740128 | 4541.915842 | 4543.564 |
| Male | 1995 | 4542.550387 | 4541.733776 | 4543.367 |
| Male | 1996 | 4546.477376 | 4545.667942 | 4547.287 |
| Male | 1997 | 4550.10304 | 4549.30081 | 4550.905 |
| Male | 1998 | 4553.652529 | 4552.857337 | 4554.448 |
| Male | 1999 | 4557.353264 | 4556.56502 | 4558.142 |
| Male | 2000 | 4560.701803 | 4559.920381 | 4561.483 |
| Male | 2001 | 4564.197756 | 4563.42313 | 4564.972 |
| Male | 2002 | 4567.661592 | 4566.893784 | 4568.429 |
| Male | 2003 | 4571.284981 | 4570.523833 | 4572.046 |
| Male | 2004 | 4575.028902 | 4574.274345 | 4575.783 |
| Male | 2005 | 4578.601016 | 4577.852954 | 4579.349 |
| Male | 2006 | 4582.385575 | 4581.644008 | 4583.127 |
| Male | 2007 | 4586.146202 | 4585.411155 | 4586.881 |
| Male | 2008 | 4590.063336 | 4589.334698 | 4590.792 |
| Male | 2009 | 4594.298644 | 4593.576267 | 4595.021 |
| Male | 2010 | 4598.569733 | 4597.85322 | 4599.286 |
| Male | 2011 | 4602.601708 | 4601.89079 | 4603.313 |
| Male | 2012 | 4606.043926 | 4605.338625 | 4606.749 |
| Male | 2013 | 4609.391678 | 4608.691856 | 4610.092 |
| Male | 2014 | 4612.81212 | 4612.117585 | 4613.507 |
| Male | 2015 | 4616.239471 | 4615.549993 | 4616.929 |
| Male | 2016 | 4619.963694 | 4619.279116 | 4620.648 |
| Male | 2017 | 4623.980949 | 4623.301127 | 4624.661 |
| Male | 2018 | 4628.083425 | 4627.40814 | 4628.759 |
| Male | 2019 | 4631.936687 | 4631.265735 | 4632.608 |
| Male | 2020 | 4635.163539 | 4634.49647 | 4635.831 |
| Male | 2021 | 4637.913851 | 4637.249791 | 4638.578 |
| Male | 2022 | 4637.025076 | 4602.959469 | 4671.091 |
| Male | 2023 | 4637.761607 | 4577.566424 | 4697.957 |
| Male | 2024 | 4638.352179 | 4545.527427 | 4731.177 |
| Male | 2025 | 4638.790573 | 4508.370918 | 4769.21 |
| Male | 2026 | 4639.288877 | 4466.948187 | 4811.63 |
| Male | 2027 | 4639.715419 | 4421.397195 | 4858.034 |
| Male | 2028 | 4640.086697 | 4372.207318 | 4907.966 |
| Male | 2029 | 4640.354479 | 4319.662989 | 4961.046 |
| Male | 2030 | 4640.479583 | 4263.959233 | 5017 |
| Male | 2031 | 4640.621567 | 4205.351032 | 5075.892 |
| Male | 2032 | 4640.667209 | 4143.724484 | 5137.61 |
| Male | 2033 | 4640.700435 | 4079.371014 | 5202.03 |
| Male | 2034 | 4640.679635 | 4012.421813 | 5268.937 |
| Male | 2035 | 4640.530654 | 3942.935556 | 5338.126 |
| Male | 2036 | 4640.335271 | 3871.024466 | 5409.646 |
| Male | 2037 | 4639.988684 | 3796.555355 | 5483.422 |
| Male | 2038 | 4639.653886 | 3719.802099 | 5559.506 |
| Male | 2039 | 4639.313806 | 3640.860276 | 5637.767 |
| Male | 2040 | 4638.876353 | 3559.736802 | 5718.016 |
| Female | 1990 | 5272.609959 | 5271.701753 | 5273.518 |
| Female | 1991 | 5245.940124 | 5245.043701 | 5246.837 |
| Female | 1992 | 5221.862547 | 5220.976857 | 5222.748 |
| Female | 1993 | 5202.548416 | 5201.672679 | 5203.424 |
| Female | 1994 | 5190.40261 | 5189.535883 | 5191.269 |
| Female | 1995 | 5187.182891 | 5186.32398 | 5188.042 |
| Female | 1996 | 5189.53893 | 5188.687394 | 5190.39 |
| Female | 1997 | 5191.88414 | 5191.04005 | 5192.728 |
| Female | 1998 | 5194.262814 | 5193.426024 | 5195.1 |
| Female | 1999 | 5196.797422 | 5195.967841 | 5197.627 |
| Female | 2000 | 5199.131624 | 5198.309066 | 5199.954 |
| Female | 2001 | 5201.707529 | 5200.891985 | 5202.523 |
| Female | 2002 | 5204.133065 | 5203.324577 | 5204.942 |
| Female | 2003 | 5206.611195 | 5205.809643 | 5207.413 |
| Female | 2004 | 5209.294038 | 5208.499337 | 5210.089 |
| Female | 2005 | 5211.804534 | 5211.01652 | 5212.593 |
| Female | 2006 | 5214.669843 | 5213.888429 | 5215.451 |
| Female | 2007 | 5217.579655 | 5216.804817 | 5218.354 |
| Female | 2008 | 5220.740887 | 5219.972497 | 5221.509 |
| Female | 2009 | 5224.230976 | 5223.468887 | 5224.993 |
| Female | 2010 | 5227.685958 | 5226.929831 | 5228.442 |
| Female | 2011 | 5231.081496 | 5230.331054 | 5231.832 |
| Female | 2012 | 5233.798529 | 5233.053733 | 5234.543 |
| Female | 2013 | 5236.349132 | 5235.609816 | 5237.088 |
| Female | 2014 | 5238.950638 | 5238.216589 | 5239.685 |
| Female | 2015 | 5241.331624 | 5240.602593 | 5242.061 |
| Female | 2016 | 5243.936842 | 5243.212616 | 5244.661 |
| Female | 2017 | 5246.562832 | 5245.843245 | 5247.282 |
| Female | 2018 | 5249.114832 | 5248.399664 | 5249.83 |
| Female | 2019 | 5251.407626 | 5250.696659 | 5252.119 |
| Female | 2020 | 5252.998857 | 5252.291779 | 5253.706 |
| Female | 2021 | 5254.321685 | 5253.617906 | 5255.025 |
| Female | 2022 | 5263.031977 | 5221.524753 | 5304.539 |
| Female | 2023 | 5264.235785 | 5191.227356 | 5337.244 |
| Female | 2024 | 5264.947543 | 5152.533719 | 5377.361 |
| Female | 2025 | 5265.335166 | 5107.498355 | 5423.172 |
| Female | 2026 | 5266.147597 | 5057.641731 | 5474.653 |
| Female | 2027 | 5267.226296 | 5003.129511 | 5531.323 |
| Female | 2028 | 5267.993859 | 4943.980302 | 5592.007 |
| Female | 2029 | 5268.279361 | 4880.441422 | 5656.117 |
| Female | 2030 | 5268.244479 | 4812.952716 | 5723.536 |
| Female | 2031 | 5268.595775 | 4742.291093 | 5794.9 |
| Female | 2032 | 5269.210199 | 4668.319584 | 5870.101 |
| Female | 2033 | 5269.551045 | 4590.816732 | 5948.285 |
| Female | 2034 | 5269.461661 | 4509.861509 | 6029.062 |
| Female | 2035 | 5269.080025 | 4425.733823 | 6112.426 |
| Female | 2036 | 5269.014387 | 4338.995662 | 6199.033 |
| Female | 2037 | 5269.164958 | 4249.492121 | 6288.838 |
| Female | 2038 | 5269.085819 | 4157.023843 | 6381.148 |
| Female | 2039 | 5268.656475 | 4061.642083 | 6475.671 |
| Female | 2040 | 5267.995556 | 3963.548502 | 6572.443 |
| Both | 1990 | 4931.694475 | 4931.069041 | 4932.32 |
| Both | 1991 | 4909.868363 | 4909.251044 | 4910.486 |
| Both | 1992 | 4890.144745 | 4889.534903 | 4890.755 |
| Both | 1993 | 4874.541251 | 4873.93831 | 4875.144 |
| Both | 1994 | 4865.186701 | 4864.589987 | 4865.783 |
| Both | 1995 | 4863.560165 | 4862.968866 | 4864.151 |
| Both | 1996 | 4866.812686 | 4866.226476 | 4867.399 |
| Both | 1997 | 4869.939609 | 4869.358523 | 4870.521 |
| Both | 1998 | 4873.048153 | 4872.472085 | 4873.624 |
| Both | 1999 | 4876.306395 | 4875.735284 | 4876.878 |
| Both | 2000 | 4879.289874 | 4878.723604 | 4879.856 |
| Both | 2001 | 4882.462419 | 4881.900982 | 4883.024 |
| Both | 2002 | 4885.544914 | 4884.988332 | 4886.101 |
| Both | 2003 | 4888.731159 | 4888.179338 | 4889.283 |
| Both | 2004 | 4892.065431 | 4891.51832 | 4892.613 |
| Both | 2005 | 4895.224288 | 4894.68179 | 4895.767 |
| Both | 2006 | 4898.645959 | 4898.108041 | 4899.184 |
| Both | 2007 | 4902.063861 | 4901.530524 | 4902.597 |
| Both | 2008 | 4905.664187 | 4905.135352 | 4906.193 |
| Both | 2009 | 4909.587536 | 4909.063101 | 4910.112 |
| Both | 2010 | 4913.548102 | 4913.027807 | 4914.068 |
| Both | 2011 | 4917.361851 | 4916.845505 | 4917.878 |
| Both | 2012 | 4920.52617 | 4920.013767 | 4921.039 |
| Both | 2013 | 4923.533987 | 4923.025425 | 4924.043 |
| Both | 2014 | 4926.578483 | 4926.073629 | 4927.083 |
| Both | 2015 | 4929.489984 | 4928.988674 | 4929.991 |
| Both | 2016 | 4932.643522 | 4932.145627 | 4933.141 |
| Both | 2017 | 4935.942189 | 4935.447598 | 4936.437 |
| Both | 2018 | 4939.238756 | 4938.747319 | 4939.73 |
| Both | 2019 | 4942.280453 | 4941.792023 | 4942.769 |
| Both | 2020 | 4944.699887 | 4944.214199 | 4945.186 |
| Both | 2021 | 4946.815777 | 4946.33243 | 4947.299 |
| Both | 2022 | 4952.641696 | 4914.853276 | 4990.43 |
| Both | 2023 | 4953.933103 | 4887.252798 | 5020.613 |
| Both | 2024 | 4954.870936 | 4852.075615 | 5057.666 |
| Both | 2025 | 4955.560894 | 4811.136902 | 5099.985 |
| Both | 2026 | 4956.516466 | 4765.657812 | 5147.375 |
| Both | 2027 | 4957.598268 | 4715.804316 | 5199.392 |
| Both | 2028 | 4958.465025 | 4661.764521 | 5255.166 |
| Both | 2029 | 4959.003154 | 4603.797983 | 5314.208 |
| Both | 2030 | 4959.29837 | 4542.245073 | 5376.352 |
| Both | 2031 | 4959.815497 | 4477.654547 | 5441.976 |
| Both | 2032 | 4960.442344 | 4409.913317 | 5510.971 |
| Both | 2033 | 4960.894328 | 4338.998558 | 5582.79 |
| Both | 2034 | 4961.068233 | 4265.010001 | 5657.126 |
| Both | 2035 | 4961.019007 | 4188.137304 | 5733.901 |
| Both | 2036 | 4961.122036 | 4108.746796 | 5813.497 |
| Both | 2037 | 4961.281203 | 4026.705224 | 5895.857 |
| Both | 2038 | 4961.301172 | 3942.00062 | 5980.602 |
| Both | 2039 | 4961.109516 | 3854.702379 | 6067.517 |
| Both | 2040 | 4960.740927 | 3764.932625 | 6156.549 |
| **ASDR** |  |  |  |  |
| Male | 1990 | 101.1603 | 101.0413 | 101.2794 |
| Male | 1991 | 100.7912 | 100.6771 | 100.9054 |
| Male | 1992 | 100.4243 | 100.3113 | 100.5373 |
| Male | 1993 | 100.0802 | 99.96827 | 100.1922 |
| Male | 1994 | 99.78871 | 99.67763 | 99.8998 |
| Male | 1995 | 99.55712 | 99.44677 | 99.66746 |
| Male | 1996 | 99.3716 | 99.26195 | 99.48125 |
| Male | 1997 | 99.17737 | 99.06838 | 99.28636 |
| Male | 1998 | 99.01121 | 98.90283 | 99.11959 |
| Male | 1999 | 98.83571 | 98.72792 | 98.9435 |
| Male | 2000 | 98.66415 | 98.55692 | 98.77137 |
| Male | 2001 | 98.50578 | 98.39913 | 98.61243 |
| Male | 2002 | 98.36254 | 98.25647 | 98.46861 |
| Male | 2003 | 98.24851 | 98.143 | 98.35401 |
| Male | 2004 | 98.14445 | 98.03952 | 98.24939 |
| Male | 2005 | 98.0304 | 97.92604 | 98.13476 |
| Male | 2006 | 97.87934 | 97.7756 | 97.98309 |
| Male | 2007 | 97.7551 | 97.65198 | 97.85821 |
| Male | 2008 | 97.6415 | 97.53902 | 97.74397 |
| Male | 2009 | 97.56774 | 97.46589 | 97.66958 |
| Male | 2010 | 97.49785 | 97.3966 | 97.59911 |
| Male | 2011 | 97.40248 | 97.30181 | 97.50316 |
| Male | 2012 | 97.23644 | 97.1364 | 97.33647 |
| Male | 2013 | 97.02587 | 96.9265 | 97.12523 |
| Male | 2014 | 96.83155 | 96.73284 | 96.93026 |
| Male | 2015 | 96.69572 | 96.59762 | 96.79383 |
| Male | 2016 | 96.63068 | 96.53313 | 96.72822 |
| Male | 2017 | 96.62082 | 96.52378 | 96.71787 |
| Male | 2018 | 96.635 | 96.53838 | 96.73162 |
| Male | 2019 | 96.57199 | 96.47573 | 96.66825 |
| Male | 2020 | 96.35258 | 96.25673 | 96.44844 |
| Male | 2021 | 96.1671 | 96.06978 | 96.26443 |
| Male | 2022 | 95.98238 | 95.18375 | 96.78102 |
| Male | 2023 | 95.82988 | 94.431 | 97.22875 |
| Male | 2024 | 95.68387 | 93.53235 | 97.83538 |
| Male | 2025 | 95.54256 | 92.52216 | 98.56297 |
| Male | 2026 | 95.40738 | 91.4171 | 99.39767 |
| Male | 2027 | 95.27839 | 90.2243 | 100.3325 |
| Male | 2028 | 95.15536 | 88.95367 | 101.3571 |
| Male | 2029 | 95.03639 | 87.61041 | 102.4624 |
| Male | 2030 | 94.92021 | 86.19822 | 103.6422 |
| Male | 2031 | 94.80774 | 84.72066 | 104.8948 |
| Male | 2032 | 94.69871 | 83.17846 | 106.219 |
| Male | 2033 | 94.59404 | 81.57655 | 107.6115 |
| Male | 2034 | 94.49273 | 79.91726 | 109.0682 |
| Male | 2035 | 94.39383 | 78.20218 | 110.5855 |
| Male | 2036 | 94.29788 | 76.43303 | 112.1627 |
| Male | 2037 | 94.20421 | 74.60952 | 113.7989 |
| Male | 2038 | 94.11421 | 72.73516 | 115.4933 |
| Male | 2039 | 94.02729 | 70.81143 | 117.2432 |
| Male | 2040 | 93.94276 | 68.83928 | 119.0462 |
| Female | 1990 | 125.413 | 125.2803 | 125.5457 |
| Female | 1991 | 124.8706 | 124.7425 | 124.9986 |
| Female | 1992 | 124.3631 | 124.2362 | 124.49 |
| Female | 1993 | 123.8839 | 123.7581 | 124.0097 |
| Female | 1994 | 123.4633 | 123.3384 | 123.5882 |
| Female | 1995 | 123.1349 | 123.0108 | 123.259 |
| Female | 1996 | 122.8654 | 122.742 | 122.9887 |
| Female | 1997 | 122.61 | 122.4874 | 122.7327 |
| Female | 1998 | 122.3678 | 122.2458 | 122.4897 |
| Female | 1999 | 122.1338 | 122.0125 | 122.2551 |
| Female | 2000 | 121.8813 | 121.7606 | 122.0019 |
| Female | 2001 | 121.6457 | 121.5257 | 121.7657 |
| Female | 2002 | 121.4167 | 121.2974 | 121.536 |
| Female | 2003 | 121.1952 | 121.0766 | 121.3139 |
| Female | 2004 | 120.9931 | 120.8752 | 121.1111 |
| Female | 2005 | 120.7453 | 120.628 | 120.8626 |
| Female | 2006 | 120.5037 | 120.3871 | 120.6203 |
| Female | 2007 | 120.2629 | 120.147 | 120.3787 |
| Female | 2008 | 120.0489 | 119.9338 | 120.164 |
| Female | 2009 | 119.8692 | 119.7549 | 119.9836 |
| Female | 2010 | 119.6968 | 119.5832 | 119.8105 |
| Female | 2011 | 119.501 | 119.388 | 119.614 |
| Female | 2012 | 119.1951 | 119.0829 | 119.3074 |
| Female | 2013 | 118.8566 | 118.7452 | 118.9681 |
| Female | 2014 | 118.5256 | 118.415 | 118.6363 |
| Female | 2015 | 118.2713 | 118.1614 | 118.3813 |
| Female | 2016 | 118.0958 | 117.9865 | 118.2051 |
| Female | 2017 | 117.9876 | 117.8789 | 118.0964 |
| Female | 2018 | 117.9112 | 117.8029 | 118.0194 |
| Female | 2019 | 117.741 | 117.6331 | 117.8488 |
| Female | 2020 | 117.3608 | 117.2534 | 117.4681 |
| Female | 2021 | 117.0257 | 116.917 | 117.1344 |
| Female | 2022 | 116.7579 | 115.8366 | 117.6792 |
| Female | 2023 | 116.459 | 114.8206 | 118.0974 |
| Female | 2024 | 116.1676 | 113.6336 | 118.7016 |
| Female | 2025 | 115.8836 | 112.3181 | 119.4492 |
| Female | 2026 | 115.6128 | 110.8979 | 120.3278 |
| Female | 2027 | 115.3538 | 109.3798 | 121.3278 |
| Female | 2028 | 115.1026 | 107.7724 | 122.4328 |
| Female | 2029 | 114.8559 | 106.081 | 123.6308 |
| Female | 2030 | 114.614 | 104.312 | 124.9161 |
| Female | 2031 | 114.3824 | 102.4735 | 126.2912 |
| Female | 2032 | 114.1595 | 100.5655 | 127.7535 |
| Female | 2033 | 113.9424 | 98.59006 | 129.2947 |
| Female | 2034 | 113.7288 | 96.54924 | 130.9085 |
| Female | 2035 | 113.5192 | 94.44635 | 132.5921 |
| Female | 2036 | 113.3185 | 92.28713 | 134.3498 |
| Female | 2037 | 113.1251 | 90.07037 | 136.1799 |
| Female | 2038 | 112.9366 | 87.79683 | 138.0765 |
| Female | 2039 | 112.751 | 85.46738 | 140.0347 |
| Female | 2040 | 112.5689 | 83.08434 | 142.0535 |
| Both | 1990 | 113.1568 | 113.0673 | 113.2463 |
| Both | 1991 | 112.6919 | 112.6049 | 112.779 |
| Both | 1992 | 112.2471 | 112.1608 | 112.3333 |
| Both | 1993 | 111.83 | 111.7445 | 111.9154 |
| Both | 1994 | 111.4698 | 111.3849 | 111.5546 |
| Both | 1995 | 111.1887 | 111.1044 | 111.2729 |
| Both | 1996 | 110.961 | 110.8772 | 111.0447 |
| Both | 1997 | 110.7359 | 110.6527 | 110.8191 |
| Both | 1998 | 110.5325 | 110.4498 | 110.6152 |
| Both | 1999 | 110.3279 | 110.2457 | 110.4102 |
| Both | 2000 | 110.1156 | 110.0338 | 110.1973 |
| Both | 2001 | 109.9181 | 109.8368 | 109.9994 |
| Both | 2002 | 109.7322 | 109.6513 | 109.813 |
| Both | 2003 | 109.5646 | 109.4843 | 109.645 |
| Both | 2004 | 109.4123 | 109.3324 | 109.4922 |
| Both | 2005 | 109.2328 | 109.1534 | 109.3123 |
| Both | 2006 | 109.0367 | 108.9578 | 109.1156 |
| Both | 2007 | 108.8551 | 108.7767 | 108.9335 |
| Both | 2008 | 108.6911 | 108.6132 | 108.769 |
| Both | 2009 | 108.5652 | 108.4878 | 108.6426 |
| Both | 2010 | 108.4459 | 108.369 | 108.5228 |
| Both | 2011 | 108.3025 | 108.226 | 108.3789 |
| Both | 2012 | 108.0677 | 107.9918 | 108.1437 |
| Both | 2013 | 107.7937 | 107.7183 | 107.8691 |
| Both | 2014 | 107.5309 | 107.456 | 107.6058 |
| Both | 2015 | 107.3351 | 107.2607 | 107.4095 |
| Both | 2016 | 107.2134 | 107.1395 | 107.2874 |
| Both | 2017 | 107.1529 | 107.0794 | 107.2265 |
| Both | 2018 | 107.121 | 107.0478 | 107.1942 |
| Both | 2019 | 107.0044 | 106.9315 | 107.0773 |
| Both | 2020 | 106.7013 | 106.6287 | 106.7739 |
| Both | 2021 | 106.4412 | 106.3682 | 106.5142 |
| Both | 2022 | 106.1983 | 105.384 | 107.0126 |
| Both | 2023 | 105.9707 | 104.5144 | 107.4269 |
| Both | 2024 | 105.7501 | 103.4907 | 108.0096 |
| Both | 2025 | 105.5356 | 102.35 | 108.7211 |
| Both | 2026 | 105.3305 | 101.1121 | 109.549 |
| Both | 2027 | 105.1344 | 99.78373 | 110.485 |
| Both | 2028 | 104.9453 | 98.37403 | 111.5165 |
| Both | 2029 | 104.7606 | 96.88806 | 112.6332 |
| Both | 2030 | 104.5796 | 95.33049 | 113.8288 |
| Both | 2031 | 104.4056 | 93.70738 | 115.1037 |
| Both | 2032 | 104.2374 | 92.0188 | 116.4561 |
| Both | 2033 | 104.0746 | 90.26854 | 117.8806 |
| Both | 2034 | 103.9152 | 88.4585 | 119.3719 |
| Both | 2035 | 103.7589 | 86.59101 | 120.9268 |
| Both | 2036 | 103.6085 | 84.66994 | 122.5471 |
| Both | 2037 | 103.4629 | 82.69437 | 124.2313 |
| Both | 2038 | 103.3216 | 80.6666 | 125.9765 |
| Both | 2039 | 103.1833 | 78.58764 | 127.7789 |
| Both | 2040 | 103.0479 | 76.45903 | 129.6367 |
