# Supplementary material for: Trends and projections of dermatitis burden (1990–2040): a 2021 global burden of disease analysis
Source: Front Med (Lausanne). 2026 Jan 28;13:1696683. doi: 10.3389/fmed.2026.1696683 (PMC12891110; doi:10.3389/fmed.2026.1696683)
Supplement: Supplementary file 6 [file Image_1.pdf]

Supplementary Figure **S1**. The global disease burden of Dermatitis for both sexes in 204 countries and territories. (A) EAPC for prevalence. (B) EAPC for incidence. (C) EAPC for DALYs.

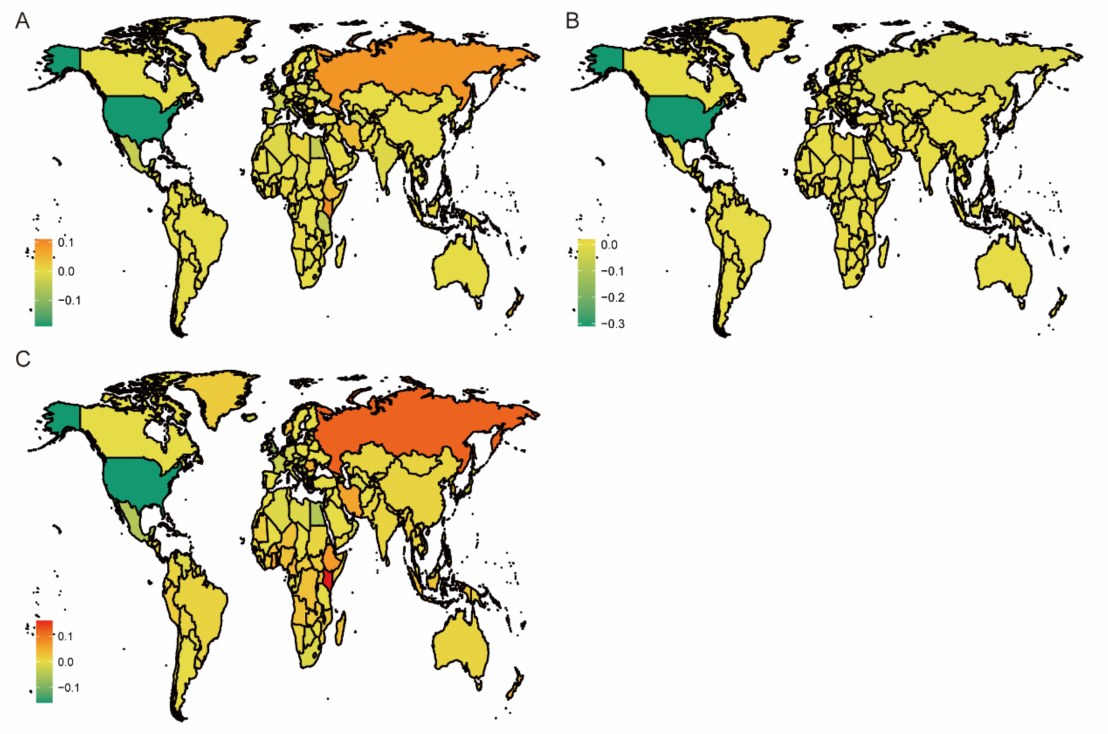

Supplementary Figure S2: The age-specific numbers and ASDRs of Dermatitis by SDI regions in 1990.

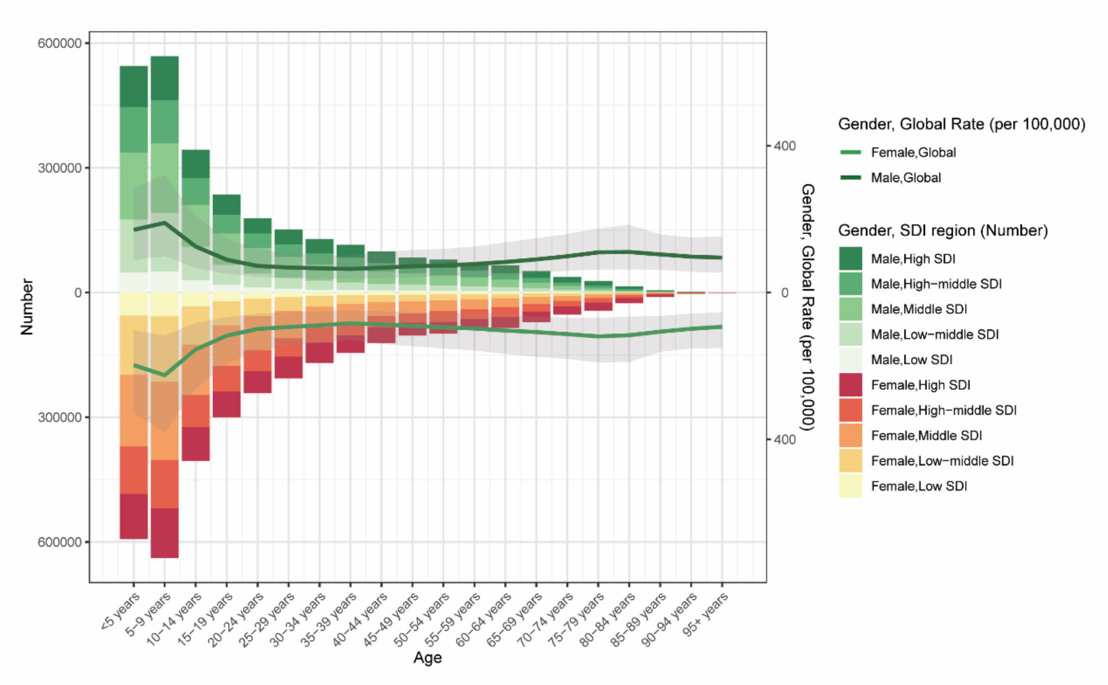

Supplementary Figure **S3**. Association between age-standardized prevalence rate, incidence rate, and DALYs rate of Dermatitis and socio-demographic index in 204 countries.

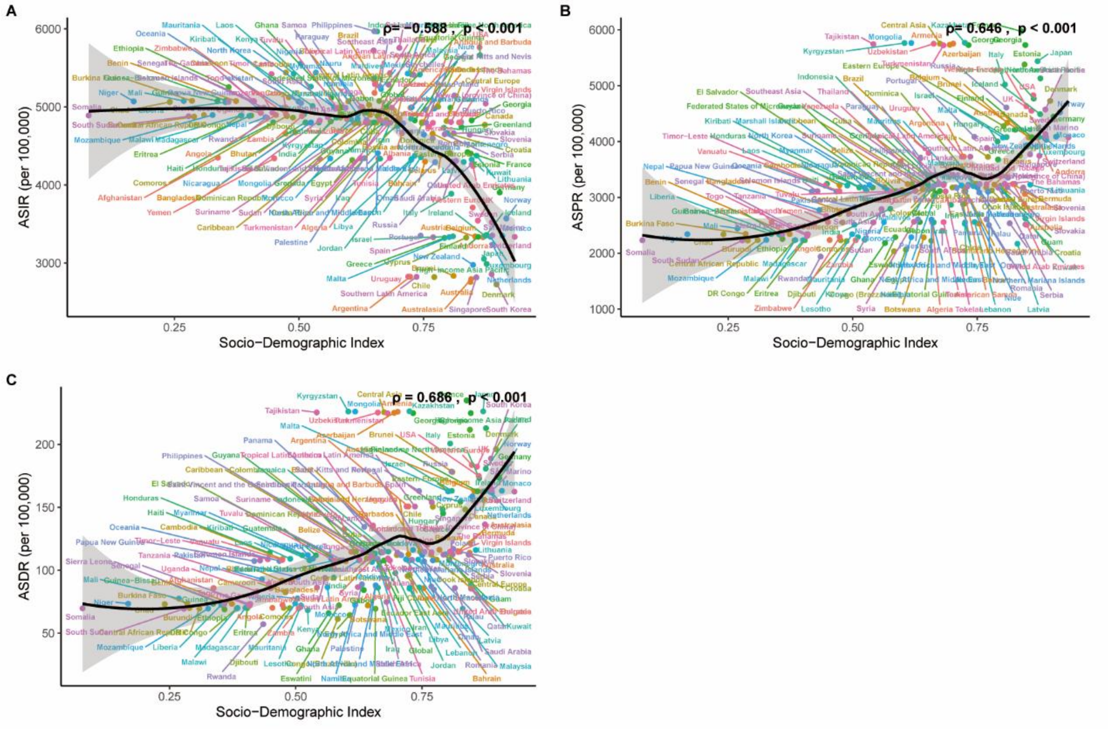

Supplementary Figure S4 ASPR, ASIR, ASDR of Dermatitis by sex, age group, and socio-demographic index, 1990 and 2021.

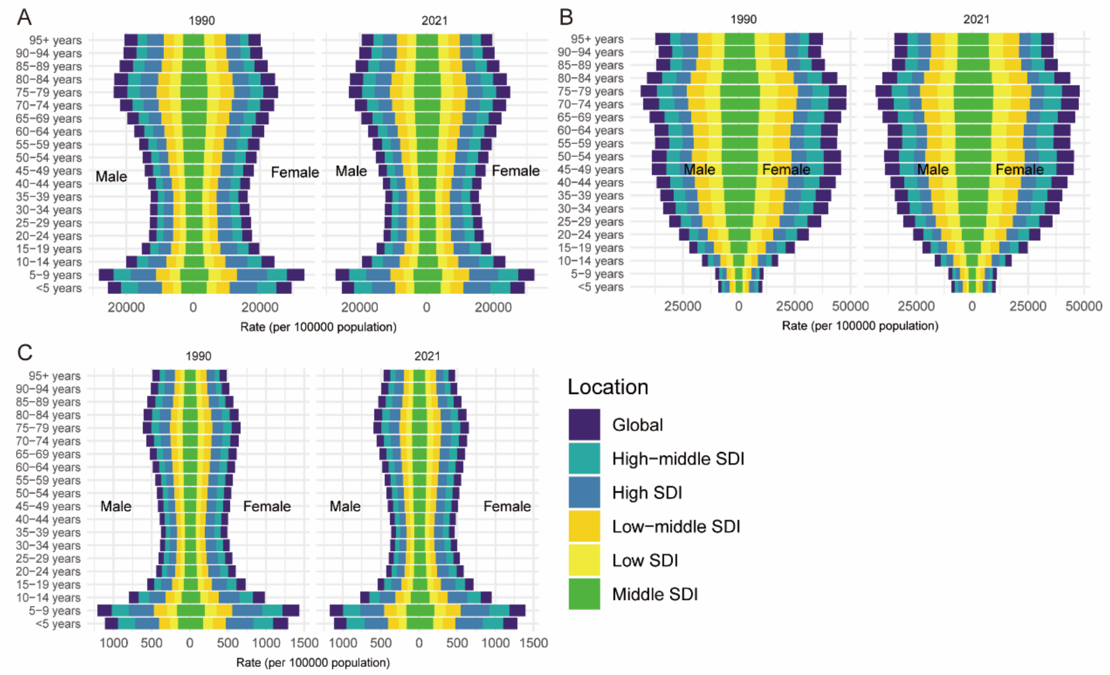

15

16
